# Dissecting microregulation of a master regulatory network

**DOI:** 10.1186/1471-2164-9-88

**Published:** 2008-02-23

**Authors:** Amit U Sinha, Vivek Kaimal, Jing Chen, Anil G Jegga

**Affiliations:** 1 Department of Computer Science, University of Cincinnati, Ohio, USA; 2 Department of Biomedical Engineering, University of Cincinnati, Ohio, USA; 3 Department of Pediatrics, University of Cincinnati College of Medicine, Ohio, USA; 4 Division of Biomedical Informatics, Cincinnati Children's Hospital Medical Center, Ohio, USA

## Abstract

**Background:**

The master regulator p53 tumor-suppressor protein through coordination of several downstream target genes and upstream transcription factors controls many pathways important for tumor suppression. While it has been reported that some of the p53's functions are microRNA-mediated, it is not known as to how many other microRNAs might contribute to the p53-mediated tumorigenesis.

**Results:**

Here, we use bioinformatics-based integrative approach to identify and prioritize putative p53-regulated miRNAs, and unravel the miRNA-based microregulation of the p53 master regulatory network. Specifically, we identify putative microRNA regulators of a) transcription factors that are upstream or downstream to p53 and b) p53 interactants. The putative *p53-miRs *and their targets are prioritized using current knowledge of cancer biology and literature-reported cancer-miRNAs.

**Conclusion:**

Our predicted p53-miRNA-gene networks strongly suggest that coordinated transcriptional and *p53-miR *mediated networks could be integral to tumorigenesis and the underlying processes and pathways.

## Background

The p53 tumor suppressor gene has been implicated as a master regulator of genomic stability, cell cycle, apoptosis, and DNA repair [1,2]. p53 is known to act as both transcriptional activator and repressor of expression of specific genes [3]. Studies of p53-mediated repression have shown that both genes that modulate apoptotic responses and genes that promote cell cycle progression can be repressed by p53 [4].

MicroRNAs (miRNAs or miRs) are small non-coding RNAs typically of 21–25 nucleotides in length and reported to be involved in the regulation of a variety of biological processes, including development, cell death, cell proliferation, hematopoiesis and nervous systems patterning [5]. miRNAs enforce posttranscriptional silencing through the RNA interference pathway [6]. Currently more than 450 human miRNAs have been identified and deposited in the miRBase [7]. Computational prediction of miRNA targets reveal that each human miRNA can potentially target several genes [8,9], underscoring the importance of these tiny noncoding RNAs in gene regulation.

Several previous studies have identified ten positive or negative feedback loops in the p53 pathway (reviewed in [10]). All of these networks or circuits are autoregulatory in that they are either induced by p53 activity at the transcriptional level, transcriptionally repressed by p53 or are regulated by p53-induced proteins [10]. Recently, five independent studies have shown that p53 upregulates miR-34a in response to DNA damage leading to cell cycle arrest and apoptosis [11-15]. In light of these reports and based on the growing evidence that microRNAs themselves act to either promote cancer or to stop cancer spread [16-19], it is reasonable to conceive that the tumor suppressor p53 might be involved in this microRNA-related network in cancer cells. In support of this, two miRNAs (miR-372 and miR-373) were found to function as oncogenes in human testicular germ cell tumors, probably by numbing the p53 pathway and thereby allowing tumorigenesis [20]. Yang et al recently suggested that analysis of microRNA regulation in the transcriptional network of the p53 gene can reveal relationships between oncogenic microRNAs and p53 [21]. We therefore sought to explore if there are other microRNAs that are components of the p53 regulatory network. In an earlier study (Kaimal and Sinha et al., unpublished), scanning the 474 human miRNAs for putative p53 sites, we identified 143 miRNAs that have at least one p53 site (using p53MH algorithm [22]) and are predicted to target at least one known gene. We termed these 143 miRNAs as *p53-miRs *(Kaimal and Sinha et al., unpublished). In the current study, apart from correlating these putative *p53-miRs *with the upstream regulators and downstream targets of p53, we prioritize our predictions using (a) literature reported differentially expressed miRNAs in cancer; (b) miRNAs reported as induced or repressed following p53 activation [11]; and (c) miRNA targets that are functionally enriched with biological processes like apoptosis, cell cycle.

## Results and discussion

### Prediction of *p53-miRs *and the target genes

A significant fraction of human intergenic miRNAs are reported to possess TSS sites within 2 kb of the pre-miRNA [23]. Although it is accepted that intronic miRNAs are generally transcribed along with their host genes [24], TSS predictions have shown them to lie predominantly in the region from -2 kb to -6 kb [23]. Hence, we limited our search space to 10 kb flanking regions of the miRNAs. Determination of exact pri-miRNA transcript lengths however is possible only through experimental work.

We used p53MH [22], an algorithm that identifies p53-responsive genes in the human and mouse genome, to identify putative p53-binding site within miRNAs (see Methods). Out of 474 human miRNAs analyzed, 227 had at least one putative p53 site (and a total of 319 p53 sites; some miRNAs have multiple p53 sites) within their flanking regions of 10 kb (Additional File 1). Of the 319 p53 sites, 5 sites were overlapping with exonic region and we did not consider these for further analysis. The 314 p53 sites (Additional File 2) occur within 10 kb flanking sequences of 223 miRNAs. We identified two p53 binding sites in mir-34a; however neither of these corresponds to the experimental validated p53 site [12] in mir-34a. This is because in the current analysis we limit the miRNA flanking sequence search space to 10 kb only while the experimentally validated p53 site in mir-34a occurs at approximately 30 kb upstream to mir-34a [12].

To predict target genes of the 223 miRNAs, we used MAMI server [25], which has compilation of five [8,9,26-28] target prediction algorithms. A recent study showed that about 82% of unique miRNA-target pairs are predicted by only a single algorithm [19]. Therefore, a combination of predictions from all algorithms might provide a much more comprehensive list of putative miRNA-target pairs than do any single prediction. 154/223 miRNAs are predicted to target at least one gene while 69/223 have no predicted targets. The 154 miRNAs mapped to 143 miRNAs. This is because some of the miRNAs have more than one predicted hairpin precursor sequences. For example mir-135a has two predicted hairpin precursors (mir-135a-1 on chromosome 3 and mir-135a-2 on chromosome 12). The targets are however based on mir-135a only. Thus, our final set consists of 143 miRNAs; each of which has at least one p53 site and is predicted to target at least one known gene. We call these 143 miRNAs as *p53-miRs*. There were 12497 genes predicted as targets of these 143 *p53-miRs*! Assuming that most of these predicted targets are purely speculative (false positive rate of at least 4 of these algorithms was estimated to be 20–30% [29]), we sought to integrate multiple types of data resources and explore as to what number of *p53-miRs *could be targeting cancer-related genes.

### MicroRNAs associated with p53 upstream and downstream genes

**Figure 1:**
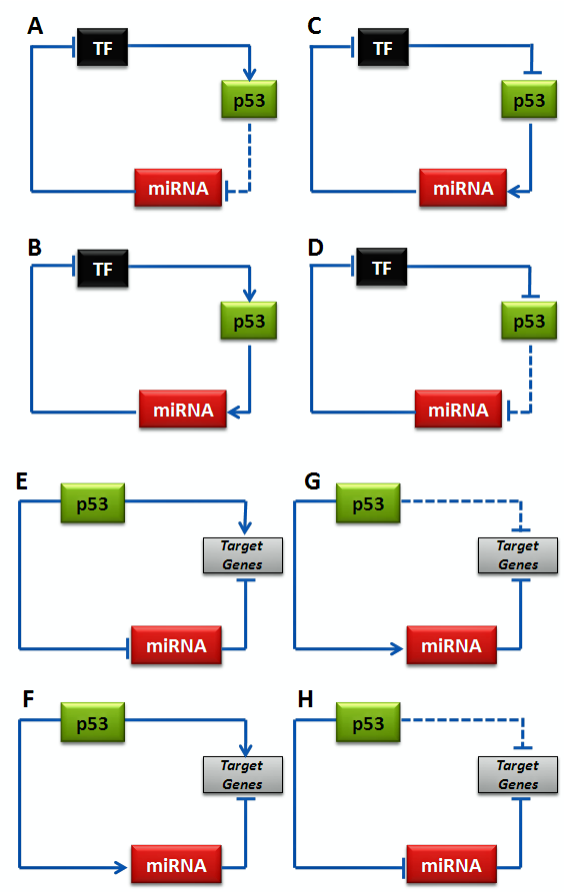
Four possible scenarios for the upstream regulators (A-D) and downstream targets (E-H) of p53. For the downstream coherent models, the p53 action on downstream target gene and miRNA is opposite (F and H). In case of downstream incoherent models (E and G), the p53 action on downstream target gene and miRNA is same. There may be a sequential gap in the activation time of the target gene and miRNA facilitating maintenance of steady states.

Transcriptional networks commonly contain positive- and negative-feedback loops, which provide robustness and fine-tuning to gene programs [30,31]. Recent studies have suggested a possible coordinated transcriptional and miRNA-mediated regulation as a recurrent motif to enhance the robustness of gene regulation in mammalian genomes [32-35]. It was also reported that miRNAs predominantly target positive regulatory motifs, highly connected scaffolds and most downstream network components such as signaling transcription factors, but less frequently target negative regulatory motifs, common components of basic cellular machines and most upstream network components such as ligands [32]. Using the p53 master regulatory network as a case study, we investigated the potential crosstalk between the miRNAs and the p53 transcriptional network itself involving transcription factors upstream and downstream to p53. At least four possible scenarios can be envisaged for the upstream regulators (Figures 1A–D) and downstream targets (Figures 1E–H) of p53.

We downloaded the known upstream transcription factor (TF) regulators and downstream target TFs of p53 from the p53 KnowledgeBase [36]. For the 23 upstream regulators and 48 downstream TFs of p53, we then extracted the putative miRNA regulators using MAMI server [25], which has compilation of five target prediction algorithms [8,9,26-28]. Since each of these five miRNA target prediction approaches generates an unpredictable number of false positives (an estimate puts it at 20–30% [29]), we intersected the results to identify the genes commonly predicted by at least two of the five algorithms [8,9,26-28] (see Table 1; for a complete list, see Additional file 3). Although it is important to confirm that a miRNA is not a potential regulatory miRNA of a specific gene by using different bioinformatics algorithms [37], there are several experimentally validated microRNA target genes that are not predicted by more than one of the current prediction algorithms. For instance the experimentally validated mir-21-*PTEN *miRNA-target pair [38] is not predicted by any of the five algorithms (listed in Methods and used in MAMI compilation). To overcome this, we also checked the TarBase [39] which has experimentally validated microRNA targets irrespective of the computational predictions.

**Table 1:** *p53-miRs *(microRNAs which have a conserved p53 binding site in their flanking 10 kb genomic sequence) which putatively target known transcription factors that are either upstream or downstream to p53 network (based on p53 knowledge base [36]) (A: activator; R: Repressor; U: uncharacterized). Only those targets are shown that are predicted as miRNA targets by 2 or more than two algorithms. miRNAs that are in bold represent miRNAs induced more than 1.5 fold (p < 0.05) after p53 activation [11]. The last column indicates whether a miRNA has been reported in literature as differentially expressed in cancer or cancer cell lines.

**miRNA**	**TF**	**TargetScanS**	**miRanda**	**microT**	**miRtarget**	**picTar**	**Relation to p53 (Interaction Type) [20]**	**Differentially expressed in cancer**
**hsa-miR-183**	*EGR1*	+	+	-	+	-	Upstream (A)	Yes
**hsa-miR-26b**	*HOXA5*	+	-	-	+	-	Upstream (A)	Yes
hsa-miR-96	*HOXA5*	+	-	-	+	-	Upstream (A)	Yes
hsa-miR-376a	*HOXA5*	-	+	+	-	-	Upstream (A)	No
hsa-miR-376b	*HOXA5*	-	+	+	-	-	Upstream (A)	No
hsa-miR-181c	*FOS*	+	+	-	+	-	Upstream (A/R)	Yes
hsa-miR-222	*FOS*	+	+	-	-	-	Upstream (A/R)	Yes
hsa-miR-101	*FOS*	+	+	-	+	-	Upstream (A/R)	No
hsa-miR-10a	*BCL6*	+	+	-	-	-	Upstream (R)	Yes
hsa-miR-124a	*KLF4*	+	-	-	+	-	Upstream (R)	Yes
hsa-miR-148a	*KLF4*	+	+	+	+	-	Upstream (R)	Yes
hsa-miR-32	*KLF4*	+	+	-	+	-	Upstream (R)	Yes
**hsa-miR-34a**	*KLF4*	+	-	-	+	-	Upstream (R)	Yes
hsa-miR-7	*KLF4*	+	-	-	+	-	Upstream (R)	Yes
hsa-miR-508	*KLF4*	-	+	-	+	-	Upstream (R)	No
**hsa-miR-155**	*CEBPB*	+	+	+	-	-	Upstream (U)	Yes
**hsa-miR-27a**	*BTG2*	+	+	+	-	-	Downstream (A)	Yes
hsa-miR-32	*BTG2*	+	+	-	-	-	Downstream (A)	Yes
**hsa-miR-27a**	*CCNG1*	+	-	-	+	-	Downstream (A)	Yes
hsa-miR-122a	*CCNG1*	+	+	-	+	-	Downstream (A)	Yes
hsa-miR-33	*EEF1A1*	+	+	-	-	-	Downstream (A)	No
hsa-miR-502	*GADD45A*	-	+	-	+	-	Downstream (A)	No
hsa-miR-19b	*IGFBP3*	+	+	-	-	-	Downstream (A)	Yes
**hsa-miR-29a**	*MMP2*	-	+	-	+	-	Downstream (A)	Yes
hsa-miR-29b	*MMP2*	-	+	-	+	-	Downstream (A)	Yes
hsa-miR-29c	*MMP2*	-	+	-	+	-	Downstream (A)	Yes
hsa-miR-128b	*PLK2*	+	+	-	+	+	Downstream (A)	Yes
hsa-miR-200b	*PLK2*	+	+	-	+	-	Downstream (A)	Yes
hsa-miR-200c	*PLK2*	+	+	-	+	-	Downstream (A)	Yes
**hsa-miR-27a**	*PLK2*	+	+	-	+	+	Downstream (A)	Yes
hsa-miR-30b	*SERPINE1*	+	+	+	+	-	Downstream (A)	Yes
hsa-miR-30c	*SERPINE1*	+	+	+	+	-	Downstream (A)	Yes
**hsa-miR-34a**	*SERPINE1*	+	-	-	+	-	Downstream (A)	Yes
hsa-miR-192	*RB1*	+	+	-	-	-	Downstream (A/R)	Yes
hsa-miR-200b	*ANLN*	+	+	-	-	-	Downstream (R)	Yes
hsa-miR-200c	*ANLN*	+	+	-	-	-	Downstream (R)	Yes
hsa-miR-142-3p	*IER3*	-	+	-	+	-	Downstream (R)	Yes
hsa-miR-124a	*SCD*	+	-	-	+	-	Downstream (R)	Yes

**Figure 2:**
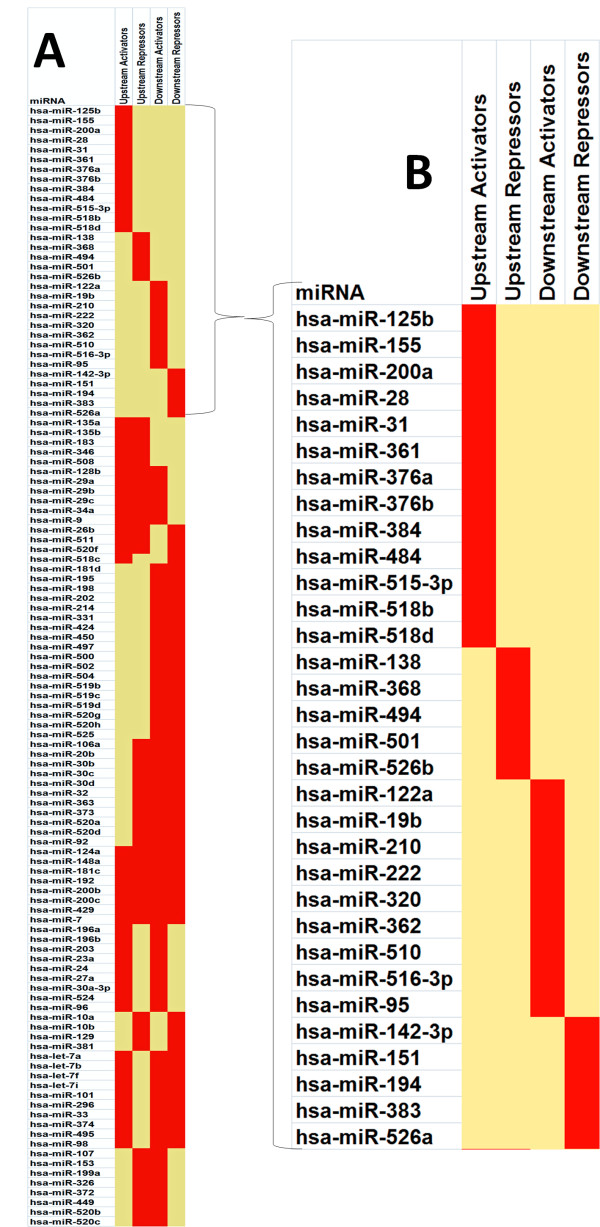
Heat map representation of *p53-miRs *and their target genes relative to the p53 network. 115 *p53-miRs *are predicted to target transcription factors that are either p53 regulators (upstream) or p53 targets (downstream) (A).

**Figure 3:**
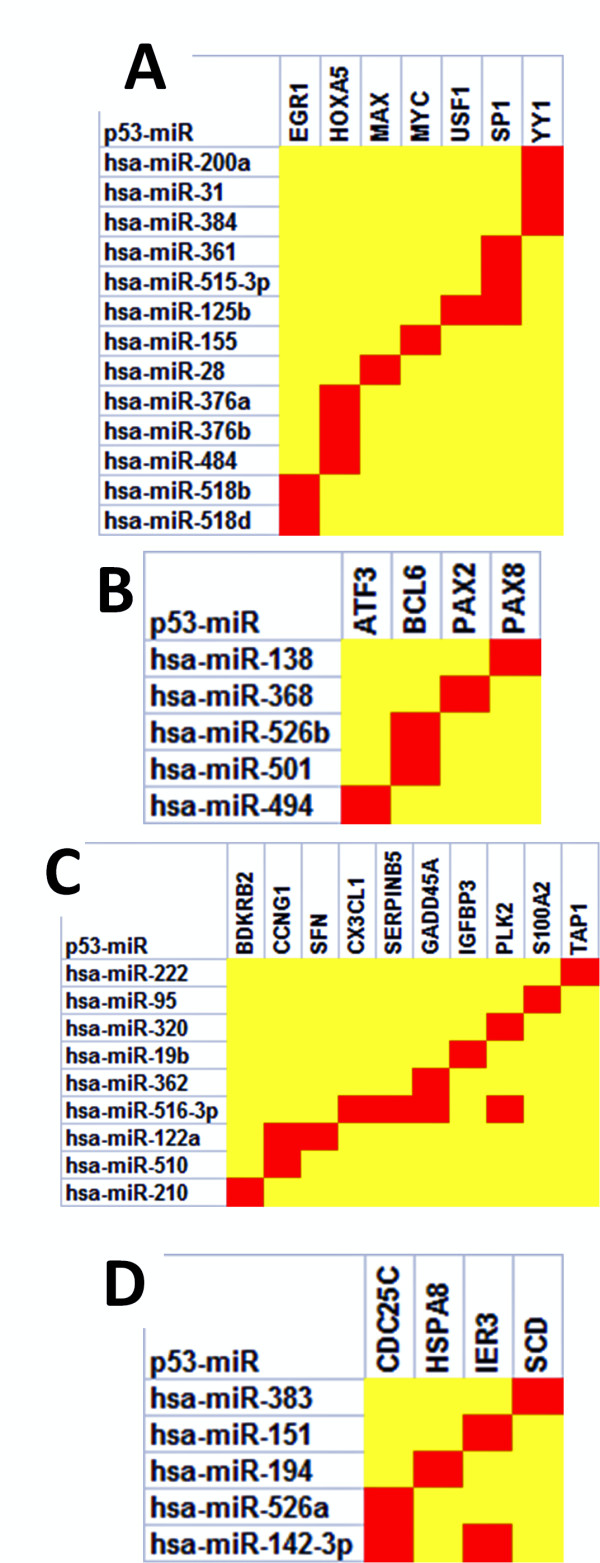
Heat map representation of *p53-miRs *and their target genes relative to the p53 network. Panels A, B, C and D depict *p53-miRs *exclusively targeting p53 upstream activators, upstream repressors, downstream activators and downstream repressors respectively along with their predicted target transcription factors.

Based on MAMI predictions, a total of 242 different miRNAs suppress p53 regulators or targets. Of these, 115 (~48%) are *p53-miRs *(Figure 2A). Some of the *p53-miRs *(32/143; Figure 2B) are found to exclusively target p53 upstream activators (13/143 *p53-miRs*; Figure 3A) or repressors (5/143; Figure 3B) or downstream activators (9/143; Figure 3C) or repressors (5/143; Figure 3D). Interestingly, many of these miRNAs have been reported as differentially expressed in various cancerous tissues or cell lines (see last column in Table 1).

### miRNAs upstream and downstream to p53: coherent and incoherent circuits

If there is a common upstream TF that regulates both the miRNA and its target gene, ideally, the transcription of the miRNAs and their targets should be oppositely regulated by this common upstream TF. For example, p53 which is known to repress the transcription of *IER3 *[40] may simultaneously activate the transcription of mir-142 (predicted to target *IER3*) that may further inhibit *IER3 *posttranscriptionally. This is a coherent model for all downstream targets that are repressed by p53. Previous genome-scale studies have in fact shown that predicted target transcripts of several tissue-specific miRNAs tend to be expressed at a lower level in tissues where the miRNAs are expressed [41,42].

**Figure 4:**
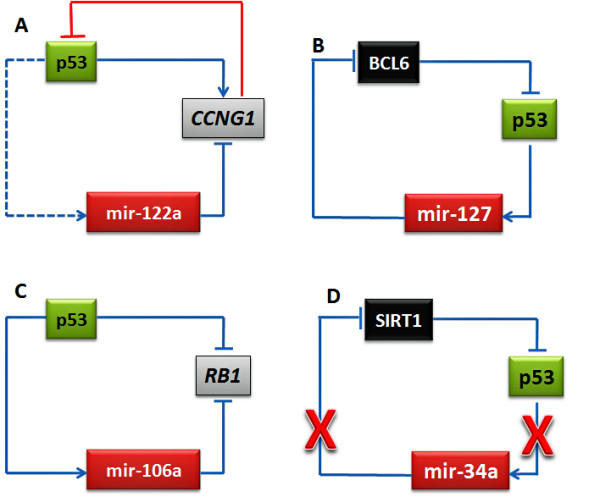
Schematic representation of *p53-miR*-mediated models downstream (A and C) and upstream (B and D) to p53.

Alternately, a common TF (p53 in this case) can activate a downstream target gene and also a miRNA which then suppresses the downstream target gene. A well known example is the experimentally confirmed microregulatory network comprising miR-17-5p and *E2F1 *– both of which are transcriptionally activated by c-Myc in human cells [43]. We report a similar example of mir-122a, a putative *p53-miR *suppressing *CCNG1*, a downstream target of p53. *CCNG1 *is a known transcriptional target (activator) of the p53 tumor suppressor protein [44] and it was recently reported that mir-122a (predicted as *p53-miR *in our analysis) has an inverse correlation with cyclin G1 expression in primary liver carcinomas [45]. In other words, p53 activates *CCNG1 *and also mir-122a which in turn suppresses *CCNG1*. This is an incoherent model. Adding to this complexity further, it is also reported that *CCNG1 *negatively regulates the stabilization of p53 in a possible negative feedback loop [46] (Figure 4A). Thus, the regulatory network wherein p53 activates a downstream target and a miR (which target the downstream target) simultaneously appears "inefficient". However, feedforward loops have the potential to provide temporal control, because expression of the ultimate target may depend on the accumulation of adequate levels of master regulator and the secondary regulators [47]. Therefore, if we consider a sequential gap in the activation time of the target genes and the miRNA, then this downstream p53-gene and *p53-miR *regulation appears coherent. Feedforward loops may provide a form of multistep ultrasensitivity [48], as any deviation from the p53's steady state would drive the downstream targets and miRNA (*p53-miR*) away from their steady states in the same direction. *p53-miRs *could therefore tune the production rate of the downstream p53 target gene opposite to the direction of p53's fluctuation. Such noise buffering probably helps to maintain target protein homeostasis and ensures more uniform expression [49] of the p53 downstream target genes within a cell population. Additionally, since the level of *p53-miR *defines the p53 downstream target's translation rate, their coexpression may allow *p53-miR *to fine-tune the downstream target's steady state. Thus, *p53-miRs *acting on p53 downstream targets could significantly shorten the response delay, leading to more effective noise buffering, as well as precise definition and maintenance of steady states.

We found two entries in TarBase (database of experimentally validated microRNA target genes) [39] which are relevant and could be interesting to p53 network (a) mir-127 targeting *BCL6 *[50], an upstream repressor of p53 [51] (Figure 4B) and (b) mir-106a reported to suppress *RB1 *[52]. A previous study has shown that mir-106a is induced more than 1.5 fold (p < 0.05) after p53 activation [11]. p53 is known to suppress *RB1 *transcription through inhibition of the basal promoter activity [53]. Additionally, posttranscriptionally, p53 might suppress *RB1 *further by inducing mir-106a, a known suppressor of *RB1 *(Figure 4C).

As a master transcriptional regulator, p53 is also known to trans-repress genes including those affecting signaling pathways such as cell proliferation, apoptosis and cytoskeleton organization [54,55]. Although, the mechanism(s) of repression is often unclear and may not be dependent on site-specific DNA-binding activity [54-57], the responsiveness of some genes to p53 can be indirect in that target genes (transactivated or transrepressed) might be other transcription factors [55,58] or microRNAs, which then modulate downstream responses to p53 signaling. We, therefore, hypothesize that in case of p53 downstream targets, the foremost activated positive feedback in a coupled feedback circuit can rapidly induce the "on" state transition of the signaling system (p53 activating downstream target genes), and that then another delayed positive feedback (p53 suppressing miRNAs that target p53 downstream target genes) robustly maintains this "on" state. Finally, the most delayed negative feedback reinstates the system in the original "off " state, preventing any further excessive response specific to the applied stimulus.

Loss of p53 expression in some tumors has also been shown to occur through inhibition of transcription of the p53 gene itself [51,59,60]. For example, mir-31, one of the *p53-miRs *predicted to suppress the upstream activator (*YY1*) of p53, is reported to be upregulated in colorectal cancer [61]. Shalgi et al recently reported that the extent of miR mediated regulation varies extensively among different genes, some of which, especially those who serve as regulators themselves, are subject to enhanced miRNA-based silencing [34].

### *p53-miR *target genes – functional over-representation analysis

Functional over-representation analysis was performed to objectively identify biological processes potentially affected by *p53-miR *target genes. Specifically, the percentage of *p53-miR *target genes with a given gene ontology (GO) annotation was compared with the percentage of whole miRNA-target genes genome-wide with the same annotation. A significant p value (p < 0.05) indicates that the observed percentage of *p53-miR *target genes with a given annotation could not likely occur by chance given the frequency of whole miRNA-target genes genome-wide with the same annotation. MAMI predicted target genes for the 143 *p53-miRs *were 12497. We used DAVID [62], and our in-house developed ToppGene server [63] for the functional enrichment analysis. Since apoptosis and growth arrest are common known consequences of p53 activation, we also tested whether *p53-miRs *tend to target apoptotic and cell proliferation related genes. Interestingly, the enrichment analysis revealed that while cell cycle regulation (p = 8.41E-4) was among the top in the *p53-miR *targets functional enrichment (p < 0.05), apoptosis was not significant. The other significantly enriched GO terms include transcription, vesicle-mediated transport, intracellular signaling cascade and cellular metabolism (Additional File 4). Interestingly, vesicle-mediated transport (p = 3.9 E-6) and endocytosis (p = 5.1 E-4) were specifically enriched in cancer-associated *p53-miR *target genes. Abnormal expression or mutation of endocytosis proteins has been reported in human cancers [64-66]. The possibility of harnessing the endocytic machinery to cancer through miRNAs could offer viable and promising leads in cancer therapy.

We extended *p53-miR *target gene functional enrichment analysis further to identify specific *p53-miRs *that significantly target apoptotic and cell proliferation genes (see Tables 2 and 3 for a list of top 25 *p53-miRs *and their target apoptotic and cell proliferation genes respectively). There was an overlap of 10 *p53-miRs *(mir-181c, mir-200a, mir-200b, mir-19b, mir-101, mir-106a, mir-142-3p, mir-195, mir-124a and mir-24) between these two groups. Earlier studies have shown that mir-23 is indeed critical for cell proliferation [67]. A recent study also implicated mir-23a, mir-24 and mir-195 in cardiac hypertrophy and reported that these miRNAs were regulated in response to stress signaling in the heart [68]. The Additional File 4 enlists the other functional categories significantly enriched within the putative *p53-miR *targets. It is to be noted that 18/25 (72%) apoptotic *p53-miRs *and 22/25 (88%) cell proliferation *p53-miRs *have been reported in literature as differentially regulated in various cancerous tissues or cancer cell lines (see last columns in Tables 2 and 3).

**Table 2:** Top 25 most significant *p53-miRs *that target known apoptotic genes (n = 685). 18/25 apoptotic *p53-miRs *have been reported in literature as differentially regulated in various cancerous tissues or cancer cell lines (see last column). The *p*-values represent the probability of a *p53-miR *targeting an apoptotic gene purely by chance.

**miRNA**	**Total Number of Predicted Targets**	**Apoptosis Genes (n = 685)**	**Genes**	**p-value**	**Cancer miRNA**
hsa-miR-30b	1054	64	*ABL1, ACTN1, ACVR1, ARHGEF6, ATG5, BCL2, BCL2L11, BCL6, BDNF, BECN1, BIRC6, BNIP3L, CASP3, CLCF1, CUL2, EBAG9, ELMO1, FOXO3A, FXR1, GAS2, GCLC, HSPA5, IFI16, IFNG, IHPK3, IL1A, JAG2, MAGI3, MAL, MAP3K5, MNT, MYBL2, NOTCH1, PAK1, PAK7, PAWR, PAX3, PDCD10, PDCD5, PIK3R2, PLAGL2, PPP2R1B, PRLR, PSEN2, RARG, RASA1, RHOB, RNF7, SGK3, SIAH2, SIRT1, SMNDC1, SNCA, SOCS3, SON, SOX9, TESK2, TIA1, TIMP3, TNFSF9, TP53INP1, UNC5C, UNC5D, VCP*	1.50E-12	Yes
hsa-miR-30c	1040	61	*ABL1, ACTN1, ACVR1, ARHGEF6, ATG5, BCL2, BCL2L11, BCL6, BDNF, BECN1, BNIP3L, CASP3, CLCF1, CUL2, EBAG9, ELMO1, FOXO3A, FXR1, GAS2, GCLC, HSPA5, IFI16, IHPK3, IL1A, JAG2, MAGI3, MAL, MAP3K5, MNT, MYBL2, NOTCH1, PAK1, PAK7, PAWR, PAX3, PDCD10, PDCD5, PIK3R2, PLAGL2, PPP2R1B, PRLR, PSEN2, RARG, RASA1, RHOB, RNF7, SGK3, SIAH2, SIRT1, SMNDC1, SNCA, SOCS3, SON, SOX9, TIA1, TIMP3, TNFSF9, TP53INP1, UNC5C, UNC5D, VCP*	2.16E-11	Yes
hsa-miR-30d	1034	57	*ABL1, ACTN1, ACVR1, ARHGEF6, ATG5, BCL2, BCL2L11, BCL6, BDNF, BECN1, BNIP3L, CARD8, CASP3, CLCF1, CUL2, EBAG9, ELMO1, FOXO3A, FXR1, GAS2, HSPA5, IFI16, IHPK3, IL1A, IL31RA, JAG2, MAL, MAP3K5, MNT, MYBL2, NOTCH1, PAK1, PAK7, PAWR, PAX3, PDCD10, PIK3R2, PLAGL2, PPP2R1B, PRLR, PSEN2, RARG, RASA1, RHOB, SGK3, SIAH2, SIRT1, SMNDC1, SOCS3, SON, SOX9, TIMP3, TNFSF9, TP53INP1, UNC5C, UNC5D, VCP*	1.03E-09	Yes
hsa-miR-495	389	31	*ACTN2, AKT1, ATOH1, AVEN, BAG4, CARD6, CASP2, CTNNAL1, DDX41, DIDO1, EEF1E1, EIF2AK2, FOXO3A, IAPP, IGFBP3, MTL5, NOTCH2, OPA1, PAWR, PDCD10, PHLPP, PIM2, RTN4, SGK, SMNDC1, SOX9, STK17A, TGFB2, TMEM23, TNFRSF9, TXNDC5*	2.17E-09	No
hsa-miR-106a	751	44	*ACIN1, ACVR1B, APBB2, APP, BCL2L11, BCL2L2, BCL6, BIRC4, BNIP2, BTG1, CASP6, CASP8, CDKN1A, CFLAR, COL4A3, DAPK2, DEDD, DNASE2, DNM2, E2F1, EGLN3, EP300, FASTK, FOXL2, HIF1A, INHBA, LALBA, MAP3K5, PAK7, PIK3R1, PLAGL2, PPARD, PPP2CA, PTEN, PURB, SQSTM1, STK17B, TAOK2, TAX1BP1, TIMP3, TMEM23, TNFRSF21, TOPORS, TP53INP1*	1.54E-08	Yes
hsa-miR-124a	1333	62	*ACTN4, AHR, AKT1S1, ANXA5, APBB2, APPBP1, ARHGDIA, AXIN1, BAG3, BCL2L11, BCL6, BECN1, CCL2, CDKN1A, CUL5, DHCR24, DNM2, DPF2, ERN1, FXR1, GAS2, GLO1, HIP1, HTATIP2, IHPK2, JAG2, LITAF, MITF, NR4A1, PDCD10, PDCD6, PEA15, PEG3, PIK3CA, PIM1, PLAGL2, PPM1F, PPP1R13L, PROC, PURB, RAF1, RARG, RASSF5, RFFL, RHOT1, RIPK2, RP6-213H19.1, SEMA6A, SFRP5, SGK, SGPL1, SGPP1, SH3KBP1, SIRT1, SLK, SOX9, SPHK1, STK4, TMEM23, TP53INP1, TRIB3, ZDHHC16*	1.06E-07	Yes
hsa-miR-20b	751	42	*ACIN1, ACVR1B, APBB2, APP, BCL2L11, BCL2L2, BCL6, BIRC4, BNIP2, BTG1, CASP6, CASP8, CASP8AP2, CDKN1A, COL4A3, DAPK2, DEDD, DNASE2, DNM2, E2F1, EGLN3, EP300, FASTK, FOXL2, HIF1A, IKIP, LALBA, MAP3K5, PAK7, PIK3R1, PLAGL2, PPP1R13L, PPP2CA, PTEN, PURB, SQSTM1, TAOK2, TIMP3, TMEM23, TNFRSF21, TOPORS, TP53INP1*,	1.18E-07	No
hsa-miR-24	445	30	*ACVR1B, ADAMTSL4, ADORA1, BCL2L11, BCL2L12, BCL2L2, CARD10, CD28, CDK5, CIDEA, CLCF1, CSF2, FASLG, IHPK2, LALBA, MNT, PAK7, PCSK9, PEA15, PIM1, PIM2, PURB, RARG, RASA1, SPHK1, TMEM23, TNFRSF19, TNFRSF1B, TP53INP1, TRAF7*,	1.66E-07	Yes
hsa-miR-27a	579	32	*ANXA5, AXUD1, BAG2, BIRC4, BNIP3L, BTG1, CDK5R1, CFDP1, EI24, FAIM, FOXO1A, HIP1, IFNG, IGF1, IL10, JMY, MITF, MLH1, NEK6, NGFR, NGFRAP1, PHB, PLAGL2, PRKRA, RASSF5, RYBP, SAP30BP, SEMA6A, SERINC3, SFRP1, TMEM23, ZNF443*	4.86E-06	Yes
hsa-miR-195	766	38	*ALOX12, APP, ARHGDIA, BAG5, BCAP29, BCL2, BCL2L2, BDNF, BFAR, CARD10, CBX4, CD28, CD40, CDK5R1, CUL2, DEDD, MNT, PAK7, PCBP4, PDCD4, PIM1, PLAGL1, PPP2R1A, PTH, PURB, RAD9A, RAF1, RASSF5, RIPK2, RYBP, SEMA6A, SGK, SIAH1, SNCA, SNRK, SON, UBE4B, ZDHHC16*,	8.00E-06	Yes
hsa-miR-182	485	28	*ACVR1, ARHGDIA, BAG4, BCL2, BCL2L12, BDNF, CFL1, CUL5, ELMO1, EP300, FOXO3A, FXR1, MITF, NCKAP1, NPM1, PIK3R1, PRKCE, RARG, RASA1, RNF130, RRAGA, RTN4, SERPINB2, TMEM23, TOPORS, TP53INP1, UNC5D, ZAK*	8.50E-06	No
hsa-miR-153	464	27	*ACTN4, ACVR1B, APBB2, APP, ATF5, ATG5, BCL2, CIB1, FOXO1A, GRIK2, IGF1R, LCK, MAP3K7, MITF, POU4F1, PRKAA1, RASA1, RTN4, SGK3, SH3KBP1, SIRT1, SNCA, SOCS2, SPHK2, TESK2, TIAL1, TP53INP1*	1.06E-05	Yes
hsa-miR-200a	634	33	*APBB2, ATF5, BAG2, CROP, CUL3, FOXO3A, HGF, IL1A, MAL, MAP3K7, MSH2, NME1, PAX3, PDCD4, PERP, PHLPP, PLAGL2, POU4F1, PPP2CA, PRKCE, SEMA6A, SIAH1, SIRT1, SON, STAT5A, STAT5B, TESK2, TGFB2, TIAL1, TP53INP1, TXNL1, UBE4B, UNC5C*	1.20E-05	Yes
hsa-miR-141	413	24	*ACVR1B, ALB, APBB2, BAG2, BCL10, DCC, EBAG9, FOXO3A, HMGB1, IL1A, NME1, PAK1, PDCD4, PERP, PHLPP, PPP2CA, PTK2B, SIAH1, SMNDC1, SNCA, SON, STAT5A, TGFB2, TP53INP1*	3.32E-05	Yes
hsa-miR-19b	715	34	*ACTN1, BAG5, BCL3, CASP8, CSE1L, CUL5, DLX1, FASTK, GULP1, HIF1A, HIP1, IGF1, IGFBP3, MAEA, MAP3K1, NGFRAP1, PIK3CA, PRKAA1, PTK2B, RAF1, RHOB, RYBP, SGK, SGPP1, SLC25A6, SOCS3, SPHK2, SULF1, TESK2, TIA1, TIMP3, TNFRSF12A, TP53INP1, TP73L*	5.53E-05	Yes
hsa-miR-518e	312	19	*ACTN4, ALS2CR2, ANXA5, BNIP3, CD5, CDC2L2, DFFB, FGFR1, FXR1, IFNG, MAPK1, PIM1, PML, PPP1R13B, RRAGC, SON, TRAIP, TRIAP1, VCP*	0.000116642	No
hsa-miR-101	627	30	*APP, BAG1, BUB1B, CD28, CDK5R1, CUL3, EBAG9, EDAR, EIF2AK3, FOXO1A, ING4, MAL, NCR1, NGFRAP1, PAX3, PLAGL1, PPP1R13B, PRKAA1, PRKCE, PURA, PURB, SEMA6A, SFRP1, SGK, SGPL1, SOCS2, SOX9, STAMBP, STK4, TP73L*	0.000134035	No
hsa-miR-183	428	23	*AXUD1, BCL6, BNIP3L, BTG1, CD3E, CDK5R1, CTSB, DAP, EI24, FOXO1A, LCK, NOTCH1, PDCD11, PDCD4, PLAGL2, PPP2CA, PRKCA, PSEN2, PURA, RHOB, TP53BP2, UNC13B, VDAC1*	1.54E-04	Yes
hsa-miR-519c	428	23	*ACVR1, ANXA4, AVEN, BAG3, BECN1, BIRC6, CAMK1D, DEDD, FASLG, FASTK, GLO1, IKIP, INHBA, NFKBIA, PTH, TAX1BP1, TEGT, TMEM23, TNFRSF10D, TNFSF18, TOPORS, TPT1, VCP*	0.000154374	No
hsa-miR-181c	824	36	*AHR, APIP, ASAH2, ATG5, BCL2, BCL2L11, BCL6, BCLAF1, CBX4, CCAR1, CEBPG, CECR2, CUL3, DNAJA3, GRIK2, HMGB1, HSPA5, IL1A, MAEA, MAP3K10, MGMT, NOTCH2, PAK7, PAWR, PDCD4, PHLDA1, PRKCE, PURB, RAD21, RNF34, SIRT1, SPP1, TIMP3, TNFRSF11B, TUBB, UNC5B*	1.82E-04	Yes
hsa-miR-107	436	23	*AATF, ACVR1, APP, BCL2L2, BDNF, CFL1, CIAPIN1, ICEBERG, IHPK3, MAP3K7, NME5, NOTCH2, PDCD10, PIK3R1, PRKCE, PTH, PURB, RAD21, RASSF5, RYBP, SNRK, THY1, VCP*	2.02E-04	Yes
hsa-miR-200b	737	33	*ALS2CR2, APBB2, ARHGDIA, BAG2, BCAP29, BIRC4, CBX4, CROP, EP300, ERN1, FOXO3A, HGF, IL1A, MLH1, MYC, NPM1, NTF3, PAK7, PDCD10, PERP, PPM1F, PPP2CA, PRKCA, PROK2, RHOT1, SIAH1, SLK, STAT5A, SULF1, TIAL1, TNFRSF11B, TUBB, TUBB2C*	2.15E-04	Yes
hsa-miR-29a	530	26	*ANGPTL4, BAK1, BCL2L7P1, CD40, DAXX, DPF1, DUSP22, ELMO2, FEM1B, IFNG, MCL1, MSH6, MYBL2, POLB, PPP1R13B, PPP2CA, PRKRA, PTEN, SGK, TNFRSF1A, TNFRSF9, TRAF4, UACA, UBE4B, UNC13B, ZNF346*	2.51E-04	Yes
hsa-miR-142-3P	364	20	*AKT1S1, BCL2L1, BIRC3, BUB1B, CRTAM, FOXO1A, HDAC1, HMGB1, IER3, IL6, PPT1, PRKDC, PRLR, PURB, RARG, RNF7, SGK, TESK2, TMEM23, TNFSF18*	3.05E-04	Yes
hsa-miR-520h	238	15	*ATF5, BRE, CASP6, CDKN2A, CFLAR, DNASE2, FASTK, HBXIP, HIF1A, IFT57, LALBA, PDIA3, STK17A, TAX1BP1, VHL*	0.000412436	No

**Table 3:** Top 25 most significant *p53-miRs *that target known cell proliferation genes (n = 712). 22/25 of these have been reported in literature as differentially regulated in various cancerous tissues or cancer cell lines (see last column). The *p*-values represent the probability of a *p53-miR *targeting a cell proliferation gene purely by chance.

**miRNA**	**Total Number of Predicted Targets**	**Cell Proliferation Genes (n = 712)**	**Genes**	**p-value**	**Cancer miRNA**
hsa-miR-23a	656	44	*ALDH1A2, BCL11B, CBFA2T2, CBFA2T3, CDC6, CDK5R1, CFDP1, CSE1L, CTNNBIP1, CUL3, CXCL5, ELF4, ELF5, EPS15, GPC4, HDGFRP3, HOXA3, IHH, IL11, IL6R, IRF2, KITLG, LRP5, MAPRE1, MDK, MET, MLLT7, NAP1L1, NOTCH2, NPM1, NR6A1, ODZ1, OPRM1, PRKRIR, PTK2B, RAP1B, SMAD3, SPOCK1, STAT5B, TGFA, TOB2, TRIB1, TUSC2, ZAK*	8.42E-10	Yes
hsa-miR-92	614	41	*ADM, AGGF1, APRIN, BMPR2, BRCA1, BTG2, CBFA2T3, CDC27, CDK6, CDKN1C, CTNNBIP1, CUL3, CXCL5, DERL2, DLG5, EDG1, EPS8, GDF11, HHIP, IGF1R, INSIG1, IRS2, KLF4, LBX1, MITF, NCK2, NF1, NOTCH1, NOX4, PAFAH1B1, PAX3, PCAF, RAP1B, RBM9, SPHK2, TBX3, TCFL5, TOB1, TOB2, UTP20, ZAK*	3.64E-09	Yes
hsa-miR-32	630	39	*ADM, AGGF1, APRIN, BMPR2, BTG2, BTLA, CBFA2T3, CDK6, CDKN1C, CHRM5, CTNNBIP1, CUL3, CXCL5, EDD1, EDG1, EPS8, EVI5, GDF11, HHIP, INSIG1, IRS2, KLF4, LBX1, MITF, NCK2, NOTCH1, NOX4, PAFAH1B1, PAX3, PCAF, RAP1B, RBM9, SPHK2, TBX3, TCFL5, TOB1, TOB2, TTK, ZAK*	6.67E-08	Yes
hsa-miR-200b	737	42	*APRIN, BAP1, BCL11B, BHLHB3, CD274, CDC25B, CNOT8, CNTFR, CTBP2, EDNRA, EPS8, ETS1, EVI5, GAB1, GLI3, HDAC4, HELLS, HGF, IL1A, KHDRBS1, KLF10, KLF4, LAMC1, LRP1, LRPAP1, MAFG, MAPRE1, MXD4, MYC, NCK2, NDN, NPM1, PCAF, POU3F2, PROK2, PTHLH, RAP1B, SESN1, SHC1, STAT5A, SUZ12, TOB1*	2.02E-07	Yes
hsa-miR-200c	751	42	*APRIN, BAP1, BCL11B, BHLHB3, BIN1, CD274, CNOT8, CNTFR, CTBP2, EDNRA, EPS8, ETS1, EVI5, GAB1, GLI3, GNAT1, HDAC4, HELLS, HGF, KHDRBS1, KLF10, KLF4, LAMC1, LRP1, LRPAP1, MAFG, MAPRE1, MXD4, MYC, NCK2, NDN, NPM1, PCAF, POU3F2, PROK2, PTHLH, RAP1B, SCG2, SESN1, SHC1, STAT5A, TOB1*	3.33E-07	Yes
hsa-miR-195	766	42	*ALOX12, AXIN2, BAI1, BDNF, BHLHB3, BIN1, BTG2, CBFA2T3, CD164, CD28, CD40, CDC25A, CDC27, CDK5R1, CHEK1, CUL2, DNAJA2, EFNB1, FGF2, FGF7, FOSL1, HDGF, HOXA3, LAMC1, MAPRE1, MNT, MTCP1, NF2, PAFAH1B1, PDAP1, PIM1, POU3F2, PPAP2A, PPM1D, PRDM4, PRKCD, PTHLH, PURB, RAF1, RARRES1, SESN1, SPEG*	5.60E-07	Yes
hsa-miR-10a	230	20	*BCAR1, BCL6, BDNF, CDK4, CTNNBIP1, FLT1, HDAC4, HOXA3, ID4, IFNAR2, JARID2, KLF11, KLF4, MAPRE1, PAFAH1B1, PURB, SERTAD1, SHC1, TRAIP, ZMYND11*	7.09E-07	Yes
hsa-miR-10b	237	20	*BCAR1, BCL6, BDNF, CDK4, CNOT8, CTNNBIP1, FLT1, HDAC4, HOXA3, ID4, IFNAR2, JARID2, KLF11, KLF4, MAPRE1, PAFAH1B1, PURB, SHC1, TRAIP, ZMYND11*	1.14E-06	Yes
hsa-miR-24	445	29	*ADM, CD164, CD28, CDK5, CDKN1B, CDV3, CNTFR, CSF2, CSK, DCTN2, EDG1, FLT1, GPC4, ING5, INSIG1, MAFG, MNT, MXI1, NUDC, PDGFRA, PIM1, PIM2, PURB, RAP1B, SERTAD1, SESN1, SPHK1, TOB2, ZNF259*	1.16E-06	Yes
hsa-miR-203	505	31	*ALDH1A2, BCL11B, CASP3, CNTFR, DLG5, EDD1, EDN1, EDNRA, FGG, GLI3, HBEGF, ID4, INSIG1, IRS2, KHDRBS1, MAPRE1, NUMBL, PDAP1, PDGFRA, PPM1D, PRKRIR, PURB, RERG, SMAD1, SMAD3, TBX3, TGFB2, TNFRSF8, TUSC2, USP8, ZMYND11*	1.77E-06	Yes
hsa-miR-124a	1333	60	*ANXA7, AR, BCL11B, BCL6, BTG2, CAPN1, CAV1, CD164, CD276, CDK6, CDKN1A, CUL5, DLG5, DLG7, EFNB1, EMP2, EPS8, ERF, ETS1, EVI1, EVI5, FGFR1OP, GAS1, GNAI2, GPC4, HDAC4, IL9R, ING1, JAG1, JAG2, KLF4, LAMC1, MAP3K11, MAPRE1, MDK, MITF, MXD4, NCK2, NRP1, OVOL2, PAFAH1B1, PBEF1, PDZK1, PGF, PIM1, PRKD1, PTHLH, PURB, QSCN6, RAF1, RAP1B, RARRES1, RHOG, SHC1, SOX9, SPHK1, SPOCK1, TAL1, TOB2, UHRF1*	1.82E-06	Yes
hsa-miR-106a	751	40	*BCL11B, BCL6, BHLHB3, BMPR2, BTG1, BTG2, BTG3, CDKN1A, COL4A3, CSF1, DERL2, E2F1, EBI3, EDD1, EDG1, EFNB1, EREG, FLT1, FZD3, GAB1, HDAC4, KLF11, LIF, MAP3K11, MAPRE1, PAFAH1B1, PCAF, PDGFRA, PPARD, PTEN, PTHLH, PURB, RB1, RBBP7, TAL1, TBX3, TGFB1, TOPORS, TSG101, TUSC2*,	2.13E-06	Yes
hsa-miR-142-3P	364	25	*BTG4, BUB1B, CDC14A, CDC25C, CRTAM, EDG3, EHF, ERG, FGFR1OP, FNTB, FOXO1A, FRK, GRN, IL6, IL6ST, KHDRBS1, KLF10, MAP3K11, MLLT7, NUMBL, PCAF, PURB, SKP2, TNFRSF13C, UTP20*,	2.50E-06	Yes
hsa-miR-33	312	22	*APRIN, BCL11B, BMPR2, CD40, CDK6, CKS2, DOCK2, FGA, FGFBP1, HIPK2, HOXC10, IRS2, LGI1, LMO1, NPM1, NPY, PDGFRA, PIM1, PURB, RB1, TSG101, UBE2V2*,	6.53E-06	No
hsa-miR-372	378	24	*BCL6, BTG1, CDCA7, CDK2, CXCR4, DERL2, DNAJA2, E2F1, ERBB4, FGF9, FRK, IRF2, MAP3K11, MNT, PCAF, PRDM4, PURB, RBBP7, TAL1, TOB2, TUSC2, UCHL1, ZFP36L2, ZMYND11*,	1.47E-05	Yes
hsa-miR-101	627	33	*BUB1B, CALCRL, CBFA2T2, CD28, CDC14A, CDC27, CDH5, CDK5R1, CDK6, CSRP2, CUL3, DLG5, ELN, EMP1, FGA, FOXO1A, GLI3, GNB1, ING4, PAX3, PBEF1, PPBP, PPP1R8, PURA, PURB, RAP1B, RBBP7, SOX9, STAMBP, TAL1, TCF19, TGFBR1, TRIB1*,	2.09E-05	No
hsa-miR-19b	715	36	*ANXA7, BMPR2, CD164, CDC2L5, CHERP, CLK1, CNTFR, CSE1L, CUL5, DLG5, DNAJA2, EDG1, EPS15, ERBB4, EREG, FOSL1, GCG, GRN, HDAC4, HHIP, HPRT1, ID4, IGF1, IMPDH1, KLF10, LIF, PCAF, POU3F2, PTK2B, RAF1, RAP1B, SMARCA2, SPHK2, ST8SIA1, SUZ12, ZMYND11*	2.29E-05	Yes
hsa-miR-196a	243	18	*ACSL6, ANXA1, ATP6AP1, CASP3, CBFA2T3, CCL23, CDKN1B, ELF4, ERG, IGFBP7, ING5, MYC, NFKBIA, PA2G4, PDGFRA, SSR1, UHRF2, ZMYND11*	2.30E-05	Yes
hsa-miR-26b	633	33	*ADM, BCL6, BTG1, CDC27, CDC2L5, CDK6, CKS2, DLG5, DNAJA2, ENPEP, EPS15, ERBB4, HGF, HIPK2, HOXD13, IGF1, IL6, JAG1, KLF10, KLF4, LIF, MITF, MXI1, PAWR, PBEF1, PDGFRA, PIM1, PRKCD, PRKCQ, PTEN, SMAD1, SMAD4, TOB1*,	2.52E-05	Yes
hsa-miR-196b	245	18	*ACSL6, ANXA1, ATP6AP1, CBFA2T3, CCL23, CDKN1B, ELF4, ERG, FOXN1, IGFBP7, ING5, MYC, NF2, NFKBIA, PA2G4, PDGFRA, SSR1, ZMYND11*	2.57E-05	Yes
hsa-miR-9	737	36	*ARHGEF2, BCL6, BIN1, BTG2, CBFA2T2, CNTFR, COL18A1, CUL4A, CXCR4, EDD1, ERG, ETS1, FGF5, FOXO1A, HDAC4, HES1, ID4, INSIG1, KITLG, LEPRE1, LIFR, NAP1L1, NOTCH2, NOX4, NRP1, ODZ1, PDGFC, PMP22, POU3F2, PPARD, RAP1B, RBM9, SHC1, TBC1D8, TGFBI, UHRF1*	4.29E-05	Yes
hsa-miR-29b	541	29	*BAX, BCL11B, CAV2, CD40, CDC7, CREG1, DUSP22, EPS15, FRAT2, FSCN1, GAB1, HDAC4, INSIG1, JARID2, LAMC1, MAFG, NASP, PDGFB, PDGFC, PINX1, PMP22, PPM1D, PRKRA, PTEN, SETDB1, SMARCA2, SPEG, TNFRSF9, UHRF2*	4.71E-05	Yes
hsa-miR-200a	634	32	*APRIN, ATP8A2, BAP1, BIN1, CD274, CDC14A, CDC25A, CDC25B, CDC2L5, CDK6, CSF3, CTBP2, CTNNB1, CUL3, FLT1, GAB1, HDAC4, HGF, IL1A, IRS2, JAG1, NME1, NRP1, PAX3, PBEF1, POU3F2, PRKD1, SCG2, STAT4, STAT5A, STAT5B, TGFB2*	6.15E-05	Yes
hsa-miR-34a	666	33	*AREG, ARX, BCL11B, BCL6, CBFA2T3, CD28, CD3E, CDC25A, CSF1R, EFNB1, ENPP7, FOSL1, GFER, GNAI2, IL6R, JAG1, KITLG, KLF4, LGI1, MET, MOV10L1, NOTCH1, NOTCH2, NUMBL, PDGFRA, PGF, PRKD1, PURB, SPEG, TGFBI, TOB2, TOPORS, UHRF2*	6.76E-05	Yes
hsa-miR-181c	824	38	*ABI1, ADAMTS1, ADM, ARHGEF2, BCL6, BMPR2, CALCRL, CBFA2T2, CBFA2T3, CKS1B, CUL3, EDG1, EPS15, ERF, ERG, ETS1, FLT1, IL1A, IRS2, LIF, LMO1, NOTCH2, NR6A1, PAFAH1B1, PAWR, PCAF, PDAP1, PDGFRA, PRDM4, PRKCD, PURB, RAP1B, RBBP7, TGFBI, TGFBR1, TGFBR2, USP8, VIP*	8.89E-05	Yes

Another interesting model is *SIRT1*-mir-34a-p53; *SIRT1 *is known to inhibit p53-mediated apoptosis by attenuation of the transcriptional activation potential of p53 primarily through deacetylation of p53 [69]. Several recent studies have shown that p53 activates mir-34a. A speculative model (Figure 4D) therefore is, in normal state mir-34a, a known *p53-miR*, suppresses *SIRT1 *(which is known to suppress p53) balancing the *SIRT1*-mediated transcriptional repression of p53. However, during tumorigenesis, increased levels of *SIRT1 *will suppress p53 triggering further suppression of p53 downstream mir-34a in a feedback loop. Examples of such models may have potential implications for cancer therapy. For example, combining DNA damage drugs (since p53 is strongly activated in response to DNA damage), *SIRT1*-mediated deacetylase inhibitors, and mir-34a activation (with "agomiR") may have synergistic effects in cancer therapy for maximally activating p53.

### *p53-miR *and p53 protein interaction networks

**Figure 5:**
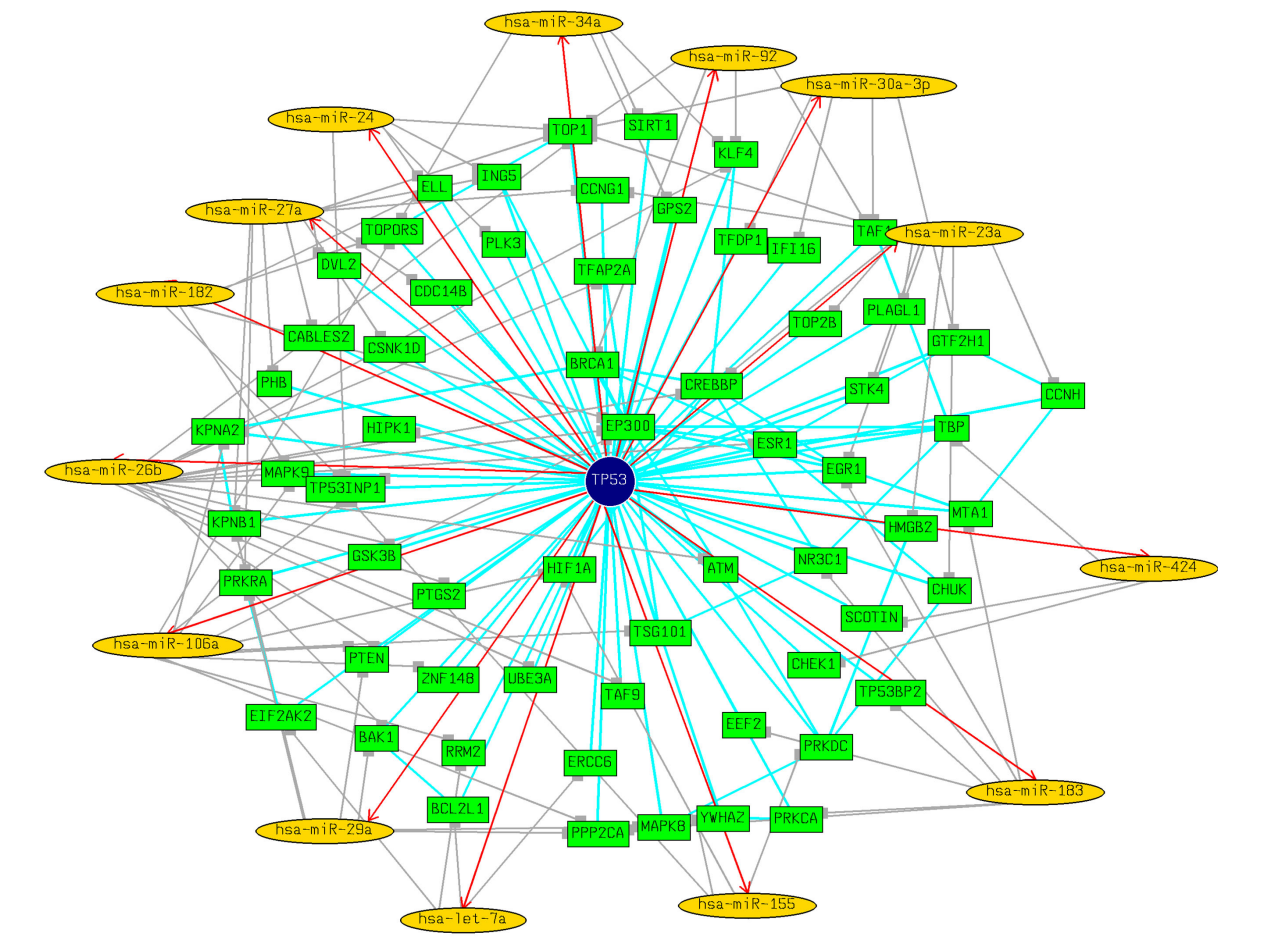
p53 interactome along with the putative miR regulators that are shown to be induced following the activation of p53 [11]. miRNAs are represented by yellow ellipses, target genes are represented by green boxes. p53 is represented in the center as blue circle. Induction of the miRNAs by p53 is represented by directed red lines. Negative regulation of the target genes by miRNAs are represented by dark grey lines. The protein interactions are represented by undirected light blue lines. Networks (using force-directed layouts) were generated using aiSee [76] network visualization software.

**Figure 6:**
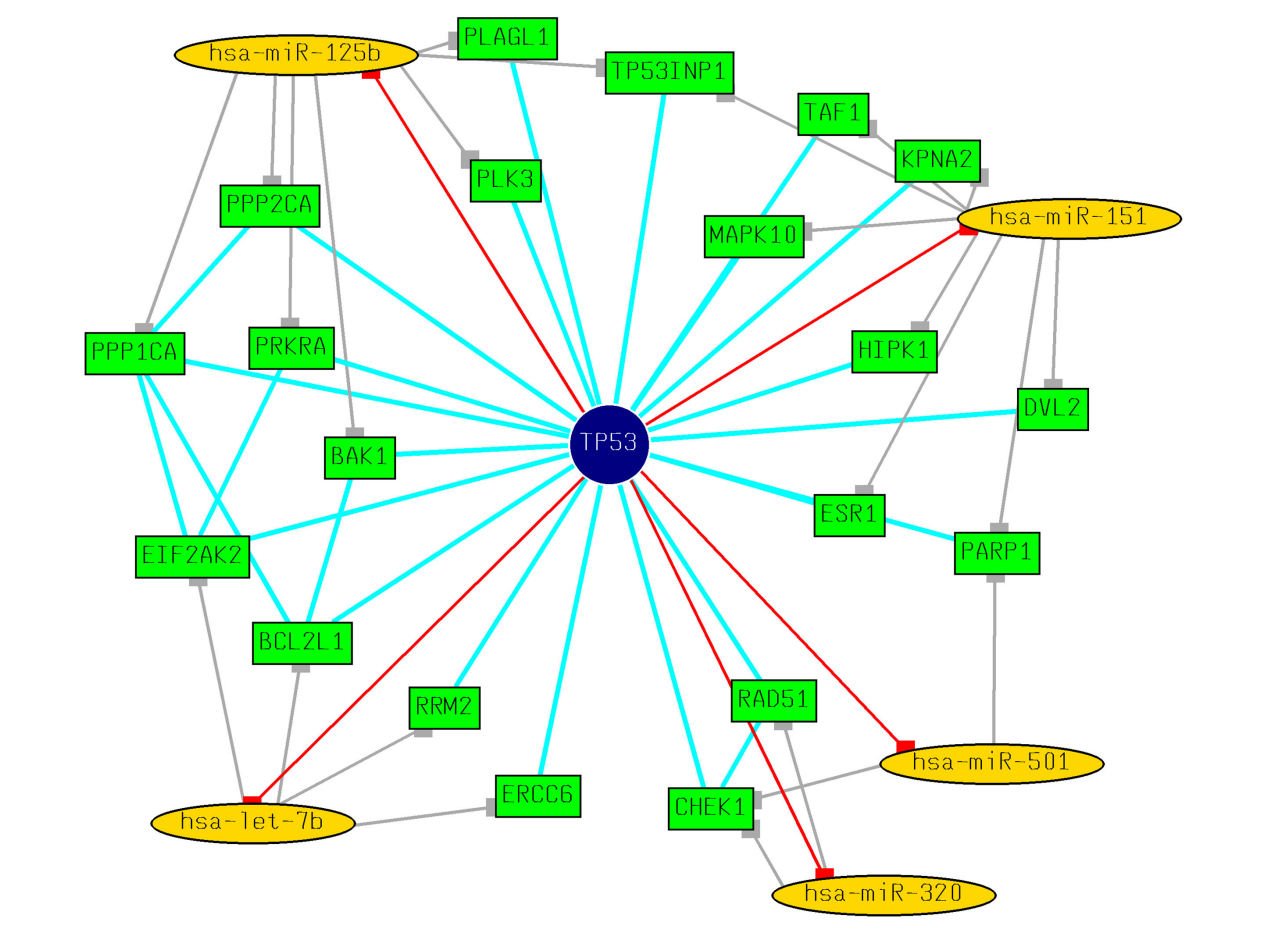
p53 interactome along with the putative miR regulators that are shown to be repressed following the activation of p53 [11]. miRNAs are represented by yellow ellipses, target genes are represented by green boxes. p53 is represented in the center as blue circle. Induction of the miRNAs by p53 is represented by directed red lines. Negative regulation of the target genes by miRNAs are represented by dark grey lines. The protein interactions are represented by undirected light blue lines. Networks (using force-directed layouts) were generated using aiSee [76] network visualization software.

Since the functional state of a protein-protein interaction network depends on gene expression, a fundamental question is what relationships exist between protein interaction network and gene regulation [70]. Liang and Li in their recent study of evidence for global correlation between microRNA repression and protein-protein interactions reported that interacting proteins tend to share more microRNA target-site types than random pairs [70]. In the current study, using the known p53-interactants, we calculated the probability of *p53-miRs *regulating the p53 interactants. We mined the BioGRID database [71], and currently there are 141 known interactants of p53. Surprisingly, 114 of these 141 p53-interactants were predicted to be regulated by *p53-miRs *and were shown to be statistically significant (*p *= 1.41e-21) (see Additional File 5 for details). Using the known data of miRNAs induced or repressed following p53 activation [11], we further filtered the *p53-miRs *that could be associated with the p53 interactome. Figures 5 and 6 show the p53 interactome along with the putative *p53-miR *regulators that are shown to be induced or repressed [11] respectively following the activation of p53.

## Conclusion

In conclusion, our bioinformatic-based analysis yielded several putative regulatory circuits that involve p53 and microRNAs along with their target genes. Although these predicted networks need to be experimentally validated to ascertain a bona fide regulatory relationship, it is still exciting to speculate that these *p53-miRs *may be critical to tumorigenesis through their regulation of the p53 master regulatory network. We strongly believe that our results substantially expand the hitherto little known repertoire of miRNAs in p53 regulatory network. It can be anticipated that further characterization of our predicted *p53-miRs *and their target genes will improve our understanding of the underlying processes of cancer, tumorigenesis, and ultimately impact response to therapy.

## Methods

### Human miRNA genomic analysis

The human miRNAs analyzed in this study (474 in total) were derived from the miRNA registry release 9.2 (May 2007; Genome Assembly NCBI-36 [72]). The genomic sequences, including 10 kb flanking sequences 5' and 3' for each of the miRNAs, were downloaded using Table Browser from the UCSC Golden Path [73].

Out of a total 474 human miRNAs analyzed, 100 microRNAs were part of a cluster (2 or more miRNAs occurring together on the genome). In all, there were 100 miRNAs organized into 37 clusters (see Additional Files 6 and 7 for details). The cluster definitions were based on those defined by Yu et al [74]. The nearest neighboring gene information (gene name, distance from the nearest miRNA, orientation with respect to the host or neighboring gene) for each of the miRNA was extracted from the public database RefFlat provided by the UCSC Golden Path [73].

### Screening miRNA genomic sequences for p53 binding sites

The genomic sequences (10 kb flanking region, 5' and 3') of the 474 human miRNAs were scanned for potential p53-binding motifs using the p53MH algorithm [22]. Briefly, p53-binds to a specific sequence comprised of two decamers (RRRCWWGYYY) separated by 0–13 bp [75]. Nearly all established human response element sequences (REs) have at least one mismatch in this degenerate sequence [55]. The computer algorithm p53MH identifies potential p53-binding sites by a scoring system based on the percentage of similarity to a consensus sequence. We set the parameter conditions to include the following restrictions: (i) a p53MH score ≥ 85%, and (ii) the spacer between the 10-bp motifs is ≤ 4 bp. Although p53 is known to bind to sites with a spacer as high as 13 bp [75], for the current analysis we considered 4 bp spacer as the maximum limit because majority of the validated p53 binding sites are reported to have a spacer less than 4 bp (see Additional Files 1 and 2 for the putative *p53-miR *distribution).

### p53 upstream and downstream activators and repressors

The group of TFs that act upstream to p53 either as activators or repressors and the p53 target genes involved in specific p53 downstream effects (activator or repressor) were downloaded from the compiled and curated dataset of the p53 knowledge-base [36]. We also mined the BioGRID database [71] for all known p53 interactants.

### *p53-miR *targets

We used MAMI [25] to download the predicted targets for the *p53-miRs*. MAMI has a compilation of target prediction tools, DIANA-microT (version 5/05) [26], miRanda (version 4/06) [8], TargetScanS (version 4/05) [9], miRtarget (version 3/06) [28] and PicTar (version 5/05) [27]. The miRNAs are based on miRs registry version 8.1 (5/06).

### Functional annotation and prioritization of *p53-miRs*

To annotate the *p53-miRs *and to identify/prioritize *p53-miRs *that might have potential implications in tumorigenesis we performed functional enrichment analysis and also intersected our predictions with cancer-associated miRNAs. Specifically, we used (a) literature reported differentially expressed miRNAs in cancer; (b) miRNAs reported as induced or repressed following p53 activation; and (c) miRNA targets that were functionally enriched with biological processes like apoptosis, cell cycle, etc.

#### Known tumor suppressor and oncogenic microRNAs

We manually compiled the list of microRNAs that were reported in literature as differentially expressed (up- or down-regulated) in various cancers in human (Additional File 8).

#### miRNAs induced or repressed following p53 activation

We intersected our predicted *p53-miRs *with the data from Tarasov et al [11] to identify potential p53 induced/repressed miRNAs. Following a genome-wide screen for microRNAs regulated by the transcription factor encoded by the p53 tumor suppressor gene, the authors reported that after p53-activation the abundance of thirty-four miRNAs was significantly increased, whereas sixteen miRNAs were suppressed [11].

#### *p53-miR *target genes – Functional enrichment

Functional over-representation analysis was performed to objectively identify biological processes potentially affected by *p53-miR *target genes. Specifically, the percentage of *p53-miR *target genes with a given gene ontology (GO) or pathway annotation was compared with the percentage of all miRNA-target genes genome-wide with the same annotation. A significant p value (p < 0.05) indicates that the observed percentage of *p53-miR *target genes with a given annotation could not likely occur by chance given the frequency of miRNA-target genes genome-wide with the same annotation. For functional enrichment analysis, we used DAVID [62] and ToppGene [63].

### *p53-miR *and p53-interactant gene networks

*p53-miR *and p53-interactant gene networks were generated using aiSee [76] with force-directed layouts. The GDL (Graphics Description Language) files, generated by a locally-implemented *JAVA *GDL library, were used as input to aiSee to create SVG (Scalable Vector Graphics) network graphs. The p53-related interactions were downloaded from BioGRID [71].

### Statistical methods

Fisher's exact test was used to calculate a p-value estimating the probability that a particular category of miRNAs is associated with a particular pattern or cluster of miRs more than would be expected by chance. Fisher's exact test was performed online at the MATFORSK [77]. For testing the functional enrichment of *p53-miR *target genes, DAVID [62] and ToppGene server [63] were used. For statistical analysis, the programming language and statistical environment R [78] were used.

## Authors' contributions

AS, VK and AJ conceived the study design which was coordinated by AJ. AS and VK designed and implemented the *p53-miR *discovery and prioritization approaches and along with AJ participated in the analysis and interpretation of results. JC carried out the p53 interactome data analysis. AJ drafted the manuscript. All the authors have read and approved the final manuscript

## Supplementary Material

Additional File 1 Systematic break-down of the putative p53-regulated miRNAs (*p53-miRs*). Schematic classification of putative p53 sites and *p53-miRs *based on the miRNA types (intragenic or intergenic). Click here for file

Additional File 2 Genomic coordinates of putative p53 sites within 10 kb flanking regions of human miRNAs. Chromosome cooridnates (based on Human Mar. 2006 – hg18 – assembly) of putative p53 sites in the flanking 10 kb regions of human microRNAs. Click here for file

Additional File 3 Putative miRNAs regulating known 23 upstream regulators and 48 downstream TFs of p53. For the known 23 upstream regulators and 48 downstream TFs of p53 (based on p53 Knowledgebase; ), putative miRs regulating them were extracted using MAMI server and the database . Click here for file

Additional File 4 Functional enrichment (based on GO and pathways) in target genes of 143 *p53-miRs*. Results of functional over-representation analysis, performed to objectively identify biological processes potentially affected by *p53-miR *target genes. Click here for file

Additional File 5 Details of Fisher's exact test used to test whether *p53-miRs *tend to target p53-interactants. Fisher's exact test was used to calculate the probability of *p53-miRs *regulating the p53 interactants (downloaded from the BioGRID database). Click here for file

Additional File 6 Systematic break-down of the 474 human miRNAs based on their genomic location. Classification of the known 474 human miRNAs based on their genomic location (intergenic or intragenic/intronic). Click here for file

Additional File 7 Details of the 474 human miRNAs analyzed. Additional details of genomic locations of the 474 human miRNAs analyzed (whether part of a cluster, i.e., 2 or more miRNAs occurring together on the genome; or located in the intergenic or intragenic/intronic regions of known genes). Click here for file

Additional File 8 Manually compiled list of miRNAs that are reported in the literature as either up or down regulated in various human cancers or cancer cell lines. This file has a list of miRNAs that are reported in the literature as either up or down regulated in various human cancers or cancer cell lines. These "cancer-associated" miRNAs were used to prioritize putative *p53-miR *targets. Click here for file
