# Supplementary figures and images for: Dissecting microregulation of a master regulatory network
Source: BMC Genomics. 2008 Feb 23;9:88. doi: 10.1186/1471-2164-9-88 (PMC2289817; doi:10.1186/1471-2164-9-88)

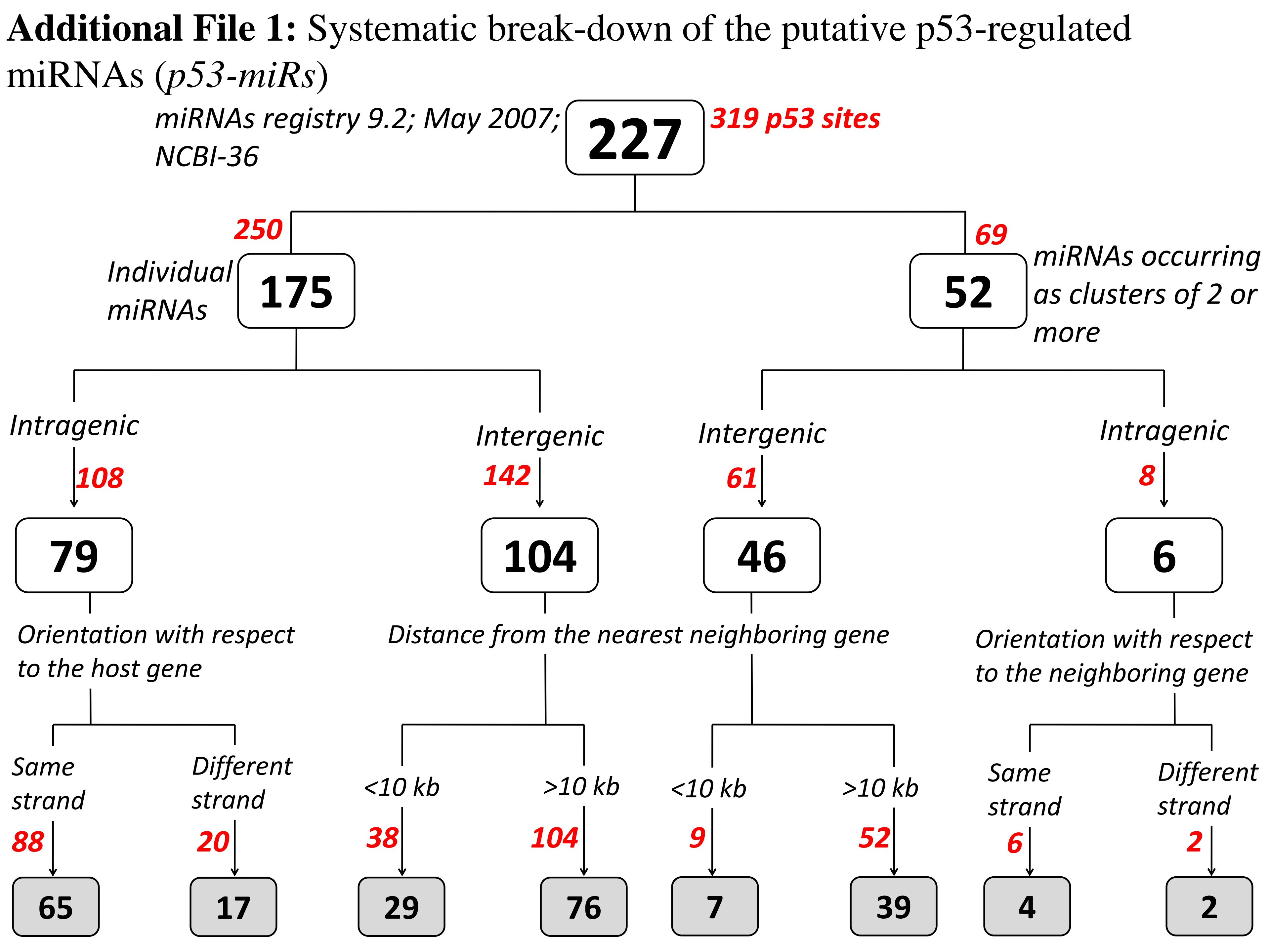

Supplement: Additional File 1 — Systematic break-down of the putative p53-regulated miRNAs (p53-miRs). Schematic classification of putative p53 sites and p53-miRs based on the miRNA types (intragenic or intergenic). [file 1471-2164-9-88-S1.pdf]

# Additional File 6: Systematic break-down of the 474 human miRNAs based on their genomic location

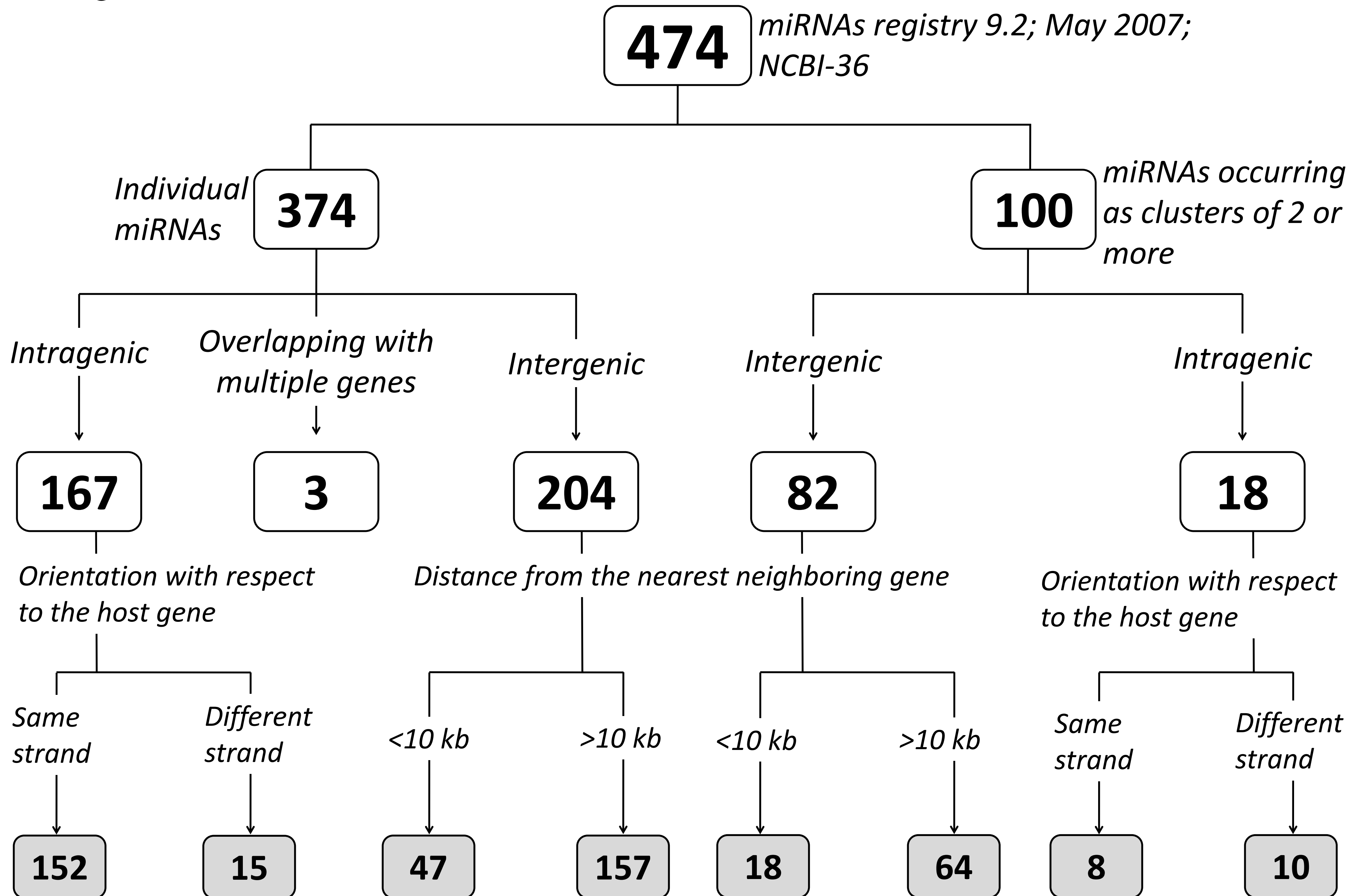

Supplement: Additional File 6 — Systematic break-down of the 474 human miRNAs based on their genomic location. Classification of the known 474 human miRNAs based on their genomic location (intergenic or intragenic/intronic). [file 1471-2164-9-88-S6.pdf]
