# Supplementary material for: Dissecting microregulation of a master regulatory network
Source: BMC Genomics. 2008 Feb 23;9:88. doi: 10.1186/1471-2164-9-88 (PMC2289817; doi:10.1186/1471-2164-9-88)
Supplement: Additional File 2 — Genomic coordinates of putative p53 sites within 10 kb flanking regions of human miRNAs. Chromosome cooridnates (based on Human Mar. 2006 – hg18 – assembly) of putative p53 sites in the flanking 10 kb regions of human microRNAs. [file 1471-2164-9-88-S2.pdf]

Additional File 2: Chromosome cooridnates (based on Human Mar. 2006 - hg18 - assembly) of putative p53 sites in the flanking 10 kb regions of human microRNAs. The start and end positions refer to the start and end of dimer 1 and dimer 2 respectively. The column "Spacer" (set to a maximum of 4 bp) indicates the number of base pairs between the two dimers.

chr	Start	End	miRNA	Strand	Spacer Length	p53 Response Element	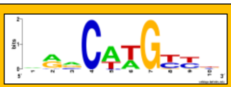 Dimer-1 (10 bp)	0 - 4 bp Spacer	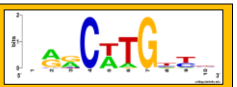 Dimer-2 (10 bp)
chr1	100521828	100521851	hsa-mir-553	+	4	AGTCAAGATTCACAAAACATGTTT	AGTCAAGATT	CACA	AAACATGTTT
chr1	155164697	155164720	hsa-mir-765	-	4	CAGCTTGTAACCTTGGGCATGTTA	CAGCTTGTAA	CCTT	GGGCATGTTA
chr10	76973718	76973741	hsa-mir-606	+	4	TGGCTTGTGCTTTCTGGCATGTTT	TGGCTTGTGC	TTTC	TGGCATGTTT
chr10	91348592	91348615	hsa-mir-107	-	4	AAGCAAGTCACTCCGAGCTAGGCA	AAGCAAGTCA	CTCC	GAGCTAGGCA
chr10	98586117	98586140	hsa-mir-607	-	4	TCACTTGCCTGATAGAACTTGCCA	TCACTTGCCT	GATA	GAACTTGCCA
chr11	121482825	121482848	hsa-mir-125b-1	-	4	CAGCTAGTCTCCTCAAACTAGTTT	CAGCTAGTCT	CCTC	AAACTAGTTT
chr12	12963453	12963476	hsa-mir-614	+	4	TGACAAGCCCATGTAGCCATGCTG	TGACAAGCCC	ATGT	AGCCATGCTG
chr12	63308685	63308708	hsa-mir-548c	+	4	AGGCATGCACCACCACACTTGCCT	AGGCATGCAC	CACC	ACACTTGCCT
chr12	96478916	96478939	hsa-mir-135a-2	+	4	CAGCAAGTGCCAAGGGGCTAGTTG	CAGCAAGTGC	CAAG	GGGCTAGTTG
chr13	98806354	98806377	hsa-mir-623	+	4	AAACAAGACTTGGGACACATGTCT	AAACAAGACT	TGGG	ACACATGTCT
chr17	44018101	44018124	hsa-mir-10a	-	4	ATGCATGCACCTAAGGACAAGCCT	ATGCATGCAC	CTAA	GGACAAGCCT
chr17	62214899	62214922	hsa-mir-634	+	4	GGGCAAGTCGGAATTAACTAGTCG	GGGCAAGTCG	GAAT	TAACTAGTCG
chr17	63930570	63930593	hsa-mir-635	-	4	AGACTTGTACAGGTTGGCTTGTTC	AGACTTGTAC	AGGT	TGGCTTGTTC
chr18	54279298	54279321	hsa-mir-122a	+	4	CAGCTTGCTTATCTAGGCTTGCAG	CAGCTTGCTT	ATCT	AGGCTTGCAG
chr19	19397341	19397364	hsa-mir-640	+	4	GACCAAGCCATAATTAGCATGCTT	GACCAAGCCA	TAAT	TAGCATGCTT
chr19	45489774	45489797	hsa-mir-641	-	4	AGACAAGCATTTGGGAACTTGCTG	AGACAAGCAT	TTGG	GAACTTGCTG
chr2	176717171	176717194	hsa-mir-10b	+	4	AAGCATGTCTCAGTCACCATGCTT	AAGCATGTCT	CAGT	CACCATGCTT
chr20	26127127	26127150	hsa-mir-663	-	4	AGTCAAGTTAAGATGAACATGCTT	AGTCAAGTTA	AGAT	GAACATGCTT
chr20	33034023	33034046	hsa-mir-499	+	4	AGACTTGCCCACTCGGGCATGCTC	AGACTTGCCC	ACTC	GGGCATGCTC
chr21	36016587	36016610	hsa-mir-802	+	4	GAGCTTGTGCAGAGAAACTTGTTT	GAGCTTGTGC	AGAG	AAACTTGTTT
chr22	36561389	36561412	hsa-mir-658	-	4	CAGCAAGCTCAGAAAATCAAGTCA	CAGCAAGCTC	AGAA	AATCAAGTCA
chr22	40619404	40619427	hsa-mir-33	+	4	TAACTTGCTTAAGGTCACAAGCCT	TAACTTGCTT	AAGG	TCACAAGCCT
chr4	83891090	83891113	hsa-mir-575	-	4	CAGCAAGTCAGCCCAGGCTAGGCT	CAGCAAGTCA	GCCC	AGGCTAGGCT
chr5	54511807	54511830	hsa-mir-449	-	4	TAGCATGTCCAGCAAGACATGTAG	TAGCATGTCC	AGCA	AGACATGTAG
chr5	54511807	54511830	hsa-mir-449b	-	4	TAGCATGTCCAGCAAGACATGTAG	TAGCATGTCC	AGCA	AGACATGTAG
chr6	72165126	72165149	hsa-mir-30a	-	4	TTACAAGTCCTTGAGAGCTTGTTT	TTACAAGTCC	TTGA	GAGCTTGTTT
chr6	119426393	119426416	hsa-mir-548b	-	4	TAACATGTAACTGCGAACATGTTT	TAACATGTAA	CTGC	GAACATGTTT
chr7	130210270	130210293	hsa-mir-29a	-	4	AGACAAGTTTAGAAGAGCAGGCTG	AGACAAGTTT	AGAA	GAGCAGGCTG
chr7	130210270	130210293	hsa-mir-29b-1	-	4	AGACAAGTTTAGAAGAGCAGGCTG	AGACAAGTTT	AGAA	GAGCAGGCTG
chr8	14746746	14746769	hsa-mir-383	-	4	ATGCAAGCCTTACAATGCAAGTCT	ATGCAAGCCT	TACA	ATGCAAGTCT
chr9	4848877	4848900	hsa-mir-101-2	+	4	AACCTTGCCCAATGAGACTTGCCA	AACCTTGCCC	AATG	AGACTTGCCA
chr9	21507138	21507161	hsa-mir-31	-	4	AAGCAAGCCAACCCCAACATGTGA	AAGCAAGCCA	ACCC	CAACATGTGA
chr9	130047774	130047797	hsa-mir-199b	-	4	AGGCATGGCTCTGCAAACATGTAG	AGGCATGGCT	CTGC	AAACATGTAG
chr9	130052611	130052634	hsa-mir-199b	-	4	GGGCTTGCCGTGGAGAGCTGGCTG	GGGCTTGCCG	TGGA	GAGCTGGCTG
chrX	49660900	49660923	hsa-mir-188	+	4	GGGCATGTCCATGTGGCCTTGTGC	GGGCATGTCC	ATGT	GGCCTTGTGC
chrX	49660900	49660923	hsa-mir-362	+	4	GGGCATGTCCATGTGGCCTTGTGC	GGGCATGTCC	ATGT	GGCCTTGTGC

chrX	49660900	49660923	hsa-mir-500	+	4	GGGCATGTCCATGTGGCCTTGTGC	GGGCATGTCC	ATGT	GGCCTTGTGC
chrX	49660900	49660923	hsa-mir-501	+	4	GGGCATGTCCATGTGGCCTTGTGC	GGGCATGTCC	ATGT	GGCCTTGTGC
chrX	49660900	49660923	hsa-mir-502	+	4	GGGCATGTCCATGTGGCCTTGTGC	GGGCATGTCC	ATGT	GGCCTTGTGC
chrX	49660900	49660923	hsa-mir-532	+	4	GGGCATGTCCATGTGGCCTTGTGC	GGGCATGTCC	ATGT	GGCCTTGTGC
chrX	49660900	49660923	hsa-mir-660	+	4	GGGCATGTCCATGTGGCCTTGTGC	GGGCATGTCC	ATGT	GGCCTTGTGC
chrX	76056742	76056765	hsa-mir-384	-	4	AGACTGGTTTAGGCCAGCTAGCTT	AGACTGGTTT	AGGC	CAGCTAGCTT
chrX	76056756	76056779	hsa-mir-384	-	4	CAGCTAGCTTGCAAATACATGCCA	CAGCTAGCTT	GCAA	ATACATGCCA
chrX	146130688	146130711	hsa-mir-508	-	4	AGACATGTCTGCCATGACTGGCTA	AGACATGTCT	GCCA	TGACTGGCTA
chr1	9124944	9124966	hsa-mir-34a	-	3	GGGCCTGCTCCAAAGGCTTGCCT	GGGCCTGCTC	CAA	AGGCTTGCCT
chr1	9129139	9129161	hsa-mir-34a	-	3	AGACAAGCCTGGGCAACATGGTG	AGACAAGCCT	GGG	CAACATGGTG
chr1	34911986	34912008	hsa-mir-552	-	3	CAGCATGTTGGCCAGGCTGGTCT	CAGCATGTTG	GCC	AGGCTGGTCT
chr1	100521137	100521159	hsa-mir-553	+	3	CACCATGTTGGCCAGGCTAGTCT	CACCATGTTG	GCC	AGGCTAGTCT
chr1	149781528	149781550	hsa-mir-554	+	3	CACCATGTTGCCCAGGCTAGTCT	CACCATGTTG	CCC	AGGCTAGTCT
chr1	154647881	154647903	hsa-mir-9-1	-	3	AGGCATGTGATCCAGGCTTGGTC	AGGCATGTGA	TCC	AGGCTTGGTC
chr1	160588669	160588691	hsa-mir-556	+	3	CACCATGTTGGCCAGGCTTGTCC	CACCATGTTG	GCC	AGGCTTGTCC
chr1	206037842	206037864	hsa-mir-29b-2	-	3	TCGCATGTTGCCCAAGCTAGTCT	TCGCATGTTG	CCC	AAGCTAGTCT
chr1	206037842	206037864	hsa-mir-29c	-	3	TCGCATGTTGCCCAAGCTAGTCT	TCGCATGTTG	CCC	AAGCTAGTCT
chr1	206040790	206040812	hsa-mir-29b-2	-	3	AGACAAGCCTGGCCAACATGATG	AGACAAGCCT	GGC	CAACATGATG
chr1	206040790	206040812	hsa-mir-29c	-	3	AGACAAGCCTGGCCAACATGATG	AGACAAGCCT	GGC	CAACATGATG
chr10	17936163	17936185	hsa-mir-511-1	+	3	TGCCATGTTGCCCAAGCTAGTCT	TGCCATGTTG	CCC	AAGCTAGTCT
chr10	18183083	18183105	hsa-mir-511-2	+	3	TGCCATGTTGCCCAAGCTAGTCT	TGCCATGTTG	CCC	AAGCTAGTCT
chr10	88018527	88018549	hsa-mir-346	-	3	AGACTAGCCTGGTCAACATGGTG	AGACTAGCCT	GGT	CAACATGGTG
chr10	98574538	98574560	hsa-mir-607	-	3	AGACTAGTCTGGCCAACATGATG	AGACTAGTCT	GGC	CAACATGATG
chr11	64408765	64408787	hsa-mir-192	-	3	AGACTAGCCTGGGTAACATGGCA	AGACTAGCCT	GGG	TAACATGGCA
chr11	64408765	64408787	hsa-mir-194-2	-	3	AGACTAGCCTGGGTAACATGGCA	AGACTAGCCT	GGG	TAACATGGCA
chr12	12804638	12804660	hsa-mir-613	+	3	AGACCAGCCTGCCCAACATGTCA	AGACCAGCCT	GCC	CAACATGTCA
chr14	100573807	100573829	hsa-mir-368	+	3	AGACTTGCTTCCTCAACAAGCTG	AGACTTGCTT	CCT	CAACAAGCTG
chr14	100573807	100573829	hsa-mir-376a-1	+	3	AGACTTGCTTCCTCAACAAGCTG	AGACTTGCTT	CCT	CAACAAGCTG
chr14	100573807	100573829	hsa-mir-376a-2	+	3	AGACTTGCTTCCTCAACAAGCTG	AGACTTGCTT	CCT	CAACAAGCTG
chr14	100573807	100573829	hsa-mir-376b	+	3	AGACTTGCTTCCTCAACAAGCTG	AGACTTGCTT	CCT	CAACAAGCTG
chr14	100573807	100573829	hsa-mir-381	+	3	AGACTTGCTTCCTCAACAAGCTG	AGACTTGCTT	CCT	CAACAAGCTG
chr14	100573807	100573829	hsa-mir-487b	+	3	AGACTTGCTTCCTCAACAAGCTG	AGACTTGCTT	CCT	CAACAAGCTG
chr14	100573807	100573829	hsa-mir-494	+	3	AGACTTGCTTCCTCAACAAGCTG	AGACTTGCTT	CCT	CAACAAGCTG
chr14	100573807	100573829	hsa-mir-495	+	3	AGACTTGCTTCCTCAACAAGCTG	AGACTTGCTT	CCT	CAACAAGCTG
chr14	100573807	100573829	hsa-mir-539	+	3	AGACTTGCTTCCTCAACAAGCTG	AGACTTGCTT	CCT	CAACAAGCTG
chr14	100573807	100573829	hsa-mir-654	+	3	AGACTTGCTTCCTCAACAAGCTG	AGACTTGCTT	CCT	CAACAAGCTG
chr14	103655627	103655649	hsa-mir-203	+	3	AAGCAAGTGACCTTGGCAAGTCC	AAGCAAGTGA	CCT	TGGCAAGTCC
chr15	40282620	40282642	hsa-mir-627	-	3	AGACTAGCCTGGCCAACATGGTG	AGACTAGCCT	GGC	CAACATGGTG
chr15	61956365	61956387	hsa-mir-422a	-	3	GGGCTTGGCCATAGGACTTGCTT	GGGCTTGGCC	ATA	GGACTTGCTT
chr15	73441525	73441547	hsa-mir-631	-	3	GGACTAGGCCATGCAACTTGTCA	GGACTAGGCC	ATG	CAACTTGTCA
chr17	25470088	25470110	hsa-mir-423	+	3	CACCATGTTGGCCGGGCTAGTCT	CACCATGTTG	GCC	GGGCTAGTCT
chr17	44071717	44071739	hsa-mir-196a-1	-	3	AGACAAGCCTGCTGGGCAAGAAC	AGACAAGCCT	GCT	GGGCAAGAAC
chr17	54564606	54564628	hsa-mir-454	-	3	AGGCTAGCCTGGCCAACATGGCA	AGGCTAGCCT	GGC	CAACATGGCA
chr17	54566493	54566515	hsa-mir-454	-	3	ATACAAGTCTTTAAAGCTTGTTT	ATACAAGTCT	TTA	AAGCTTGTTT
chr17	58374950	58374972	hsa-mir-633	+	3	TGCCTTGTTTACTGAACATGTCC	TGCCTTGTTT	ACT	GAACATGTCC
chr17	62220979	62221001	hsa-mir-634	+	3	TAGCTTGTTTAGAGAGCTTGATG	TAGCTTGTTT	AGA	GAGCTTGATG

chr19	3911576	3911598	hsa-mir-637	-	3	AGACAAGCCTGGGCAACATGGTG	AGACAAGCCT	GGG	CAACATGGTG
chr19	4729641	4729663	hsa-mir-7-3	+	3	TACCATGTTGGCCAAGCTAGTCT	TACCATGTTG	GCC	AAGCTAGTCT
chr19	13817218	13817240	hsa-mir-23a	-	3	AGACAAGCCTGGCCAACATGGTG	AGACAAGCCT	GGC	CAACATGGTG
chr19	13817218	13817240	hsa-mir-24-2	-	3	AGACAAGCCTGGCCAACATGGTG	AGACAAGCCT	GGC	CAACATGGTG
chr19	13817218	13817240	hsa-mir-27a	-	3	AGACAAGCCTGGCCAACATGGTG	AGACAAGCCT	GGC	CAACATGGTG
chr19	19407230	19407252	hsa-mir-640	+	3	CACCATGTTGCCCGGGCTTGTCT	CACCATGTTG	CCC	GGGCTTGTCT
chr19	45470625	45470647	hsa-mir-641	-	3	GGGCAAGTCACCAAGTCATGTTC	GGGCAAGTCA	CCA	AGTCATGTTC
chr19	45487364	45487386	hsa-mir-641	-	3	GGACAAGCTGTGGGGACTAGTCA	GGACAAGCTG	TGG	GGACTAGTCA
chr19	51208451	51208473	hsa-mir-769	+	3	TACCTTGTTGCCCAGGCATGTCT	TACCTTGTTG	CCC	AGGCATGTCT
chr19	58885368	58885390	hsa-mir-515-2	+	3	CACCATGTTGCCCAGGCTTGTCT	CACCATGTTG	CCC	AGGCTTGTCT
chr19	58885368	58885390	hsa-mir-518f	+	3	CACCATGTTGCCCAGGCTTGTCT	CACCATGTTG	CCC	AGGCTTGTCT
chr19	58885368	58885390	hsa-mir-519b	+	3	CACCATGTTGCCCAGGCTTGTCT	CACCATGTTG	CCC	AGGCTTGTCT
chr19	58885368	58885390	hsa-mir-519c	+	3	CACCATGTTGCCCAGGCTTGTCT	CACCATGTTG	CCC	AGGCTTGTCT
chr19	58885368	58885390	hsa-mir-520a	+	3	CACCATGTTGCCCAGGCTTGTCT	CACCATGTTG	CCC	AGGCTTGTCT
chr19	58885368	58885390	hsa-mir-520f	+	3	CACCATGTTGCCCAGGCTTGTCT	CACCATGTTG	CCC	AGGCTTGTCT
chr19	58885368	58885390	hsa-mir-523	+	3	CACCATGTTGCCCAGGCTTGTCT	CACCATGTTG	CCC	AGGCTTGTCT
chr19	58885368	58885390	hsa-mir-525	+	3	CACCATGTTGCCCAGGCTTGTCT	CACCATGTTG	CCC	AGGCTTGTCT
chr19	58885368	58885390	hsa-mir-526b	+	3	CACCATGTTGCCCAGGCTTGTCT	CACCATGTTG	CCC	AGGCTTGTCT
chr19	58897055	58897077	hsa-mir-518b	+	3	CACCATGTTGCCCAGGCTAGTCT	CACCATGTTG	CCC	AGGCTAGTCT
chr19	58897055	58897077	hsa-mir-518c	+	3	CACCATGTTGCCCAGGCTAGTCT	CACCATGTTG	CCC	AGGCTAGTCT
chr19	58897055	58897077	hsa-mir-518f	+	3	CACCATGTTGCCCAGGCTAGTCT	CACCATGTTG	CCC	AGGCTAGTCT
chr19	58897055	58897077	hsa-mir-519b	+	3	CACCATGTTGCCCAGGCTAGTCT	CACCATGTTG	CCC	AGGCTAGTCT
chr19	58897055	58897077	hsa-mir-520b	+	3	CACCATGTTGCCCAGGCTAGTCT	CACCATGTTG	CCC	AGGCTAGTCT
chr19	58897055	58897077	hsa-mir-520c	+	3	CACCATGTTGCCCAGGCTAGTCT	CACCATGTTG	CCC	AGGCTAGTCT
chr19	58897055	58897077	hsa-mir-523	+	3	CACCATGTTGCCCAGGCTAGTCT	CACCATGTTG	CCC	AGGCTAGTCT
chr19	58897055	58897077	hsa-mir-524	+	3	CACCATGTTGCCCAGGCTAGTCT	CACCATGTTG	CCC	AGGCTAGTCT
chr19	58897055	58897077	hsa-mir-525	+	3	CACCATGTTGCCCAGGCTAGTCT	CACCATGTTG	CCC	AGGCTAGTCT
chr19	58897055	58897077	hsa-mir-526a-1	+	3	CACCATGTTGCCCAGGCTAGTCT	CACCATGTTG	CCC	AGGCTAGTCT
chr19	58897055	58897077	hsa-mir-526b	+	3	CACCATGTTGCCCAGGCTAGTCT	CACCATGTTG	CCC	AGGCTAGTCT
chr19	58907932	58907954	hsa-mir-517a	+	3	CACCATGTTGGCCAGGCTAGTCT	CACCATGTTG	GCC	AGGCTAGTCT
chr19	58907932	58907954	hsa-mir-517b	+	3	CACCATGTTGGCCAGGCTAGTCT	CACCATGTTG	GCC	AGGCTAGTCT
chr19	58907932	58907954	hsa-mir-518c	+	3	CACCATGTTGGCCAGGCTAGTCT	CACCATGTTG	GCC	AGGCTAGTCT
chr19	58907932	58907954	hsa-mir-519d	+	3	CACCATGTTGGCCAGGCTAGTCT	CACCATGTTG	GCC	AGGCTAGTCT
chr19	58907932	58907954	hsa-mir-520c	+	3	CACCATGTTGGCCAGGCTAGTCT	CACCATGTTG	GCC	AGGCTAGTCT
chr19	58907932	58907954	hsa-mir-520d	+	3	CACCATGTTGGCCAGGCTAGTCT	CACCATGTTG	GCC	AGGCTAGTCT
chr19	58907932	58907954	hsa-mir-520g	+	3	CACCATGTTGGCCAGGCTAGTCT	CACCATGTTG	GCC	AGGCTAGTCT
chr19	58907932	58907954	hsa-mir-521-2	+	3	CACCATGTTGGCCAGGCTAGTCT	CACCATGTTG	GCC	AGGCTAGTCT
chr19	58907932	58907954	hsa-mir-524	+	3	CACCATGTTGGCCAGGCTAGTCT	CACCATGTTG	GCC	AGGCTAGTCT
chr19	58907932	58907954	hsa-mir-526a-1	+	3	CACCATGTTGGCCAGGCTAGTCT	CACCATGTTG	GCC	AGGCTAGTCT
chr19	58909150	58909172	hsa-mir-517a	+	3	CACCATGTTGGCCAGGCTAGTCT	CACCATGTTG	GCC	AGGCTAGTCT
chr19	58909150	58909172	hsa-mir-517b	+	3	CACCATGTTGGCCAGGCTAGTCT	CACCATGTTG	GCC	AGGCTAGTCT
chr19	58909150	58909172	hsa-mir-518c	+	3	CACCATGTTGGCCAGGCTAGTCT	CACCATGTTG	GCC	AGGCTAGTCT
chr19	58909150	58909172	hsa-mir-519d	+	3	CACCATGTTGGCCAGGCTAGTCT	CACCATGTTG	GCC	AGGCTAGTCT
chr19	58909150	58909172	hsa-mir-520c	+	3	CACCATGTTGGCCAGGCTAGTCT	CACCATGTTG	GCC	AGGCTAGTCT
chr19	58909150	58909172	hsa-mir-520d	+	3	CACCATGTTGGCCAGGCTAGTCT	CACCATGTTG	GCC	AGGCTAGTCT
chr19	58909150	58909172	hsa-mir-520g	+	3	CACCATGTTGGCCAGGCTAGTCT	CACCATGTTG	GCC	AGGCTAGTCT

chr19	58909150	58909172	hsa-mir-521-2	+	3	CACCATGTTGGCCAGGCTAGTCT	CACCATGTTG	GCC	AGGCTAGTCT
chr19	58909150	58909172	hsa-mir-524	+	3	CACCATGTTGGCCAGGCTAGTCT	CACCATGTTG	GCC	AGGCTAGTCT
chr19	58909150	58909172	hsa-mir-526a-1	+	3	CACCATGTTGGCCAGGCTAGTCT	CACCATGTTG	GCC	AGGCTAGTCT
chr19	58981616	58981638	hsa-mir-371	+	3	CGCCATGTTGCCCAGGCTAGTCT	CGCCATGTTG	CCC	AGGCTAGTCT
chr19	58981616	58981638	hsa-mir-372	+	3	CGCCATGTTGCCCAGGCTAGTCT	CGCCATGTTG	CCC	AGGCTAGTCT
chr19	58981616	58981638	hsa-mir-373	+	3	CGCCATGTTGCCCAGGCTAGTCT	CGCCATGTTG	CCC	AGGCTAGTCT
chr2	176714186	176714208	hsa-mir-10b	+	3	GAACTAGCCAGATGCGCATGTCT	GAACTAGCCA	GAT	GCGCATGTCT
chr2	188871961	188871983	hsa-mir-561	+	3	CAGCTAGTCCCTACTGCTTGTTT	CAGCTAGTCC	CTA	CTGCTTGTTT
chr2	188876771	188876793	hsa-mir-561	+	3	AAACATGCTTGAGATGCTTGCTT	AAACATGCTT	GAG	ATGCTTGCTT
chr2	218966641	218966663	hsa-mir-26b	+	3	CATCATGTTGGCCAGGCTAGTCT	CATCATGTTG	GCC	AGGCTAGTCT
chr2	218984892	218984914	hsa-mir-26b	+	3	CACCATGTTGGCCAGGCTAGTCT	CACCATGTTG	GCC	AGGCTAGTCT
chr2	232749041	232749063	hsa-mir-562	+	3	CACCATGTTGGCCAGGCTAGTCT	CACCATGTTG	GCC	AGGCTAGTCT
chr20	48638570	48638592	hsa-mir-645	+	3	AAACATGATCATAAGACATGATT	AAACATGATC	ATA	AGACATGATT
chr20	56817091	56817113	hsa-mir-296	-	3	AGGCTTGTCTGGGGGACTTGGGG	AGGCTTGTCT	GGG	GGACTTGGGG
chr20	56826344	56826366	hsa-mir-296	-	3	AAACATGCACATCCAGCTTGCCT	AAACATGCAC	ATC	CAGCTTGCCT
chr22	36569498	36569520	hsa-mir-658	-	3	AAACAAGCTGGTACGACTTGGCT	AAACAAGCTG	GTA	CGACTTGGCT
chr22	36569498	36569520	hsa-mir-659	-	3	AAACAAGCTGGTACGACTTGGCT	AAACAAGCTG	GTA	CGACTTGGCT
chr22	36571621	36571643	hsa-mir-658	-	3	AGACAAGCCTGGCCAACATGATG	AGACAAGCCT	GGC	CAACATGATG
chr22	36571621	36571643	hsa-mir-659	-	3	AGACAAGCCTGGCCAACATGATG	AGACAAGCCT	GGC	CAACATGATG
chr22	44888996	44889018	hsa-let-7a-3	+	3	GGGCATGTGGGGTGGGCTTGTTC	GGGCATGTGG	GGT	GGGCTTGTTC
chr22	44888996	44889018	hsa-let-7b	+	3	GGGCATGTGGGGTGGGCTTGTTC	GGGCATGTGG	GGT	GGGCTTGTTC
chr3	52300727	52300749	hsa-mir-135a-1	-	3	GGGCTTGTCACCCCTGCTTGCCT	GGGCTTGTCA	CCC	CTGCTTGCCT
chr3	189889173	189889195	hsa-mir-28	+	3	TGGCTTGTCTATTCAGCAAGCAC	TGGCTTGTCT	ATT	CAGCAAGCAC
chr3	196914412	196914434	hsa-mir-570	+	3	CGCCATGTTAGCCAGGCTAGTCT	CGCCATGTTA	GCC	AGGCTAGTCT
chr4	337447	337469	hsa-mir-571	+	3	CACCATGTTGGCCAGGCTAGTCT	CACCATGTTG	GCC	AGGCTAGTCT
chr4	24129687	24129709	hsa-mir-573	-	3	AGACAAGCCTGGCCAACATGGCG	AGACAAGCCT	GGC	CAACATGGCG
chr4	83902149	83902171	hsa-mir-575	-	3	AGACTAGCCTGGGCAACATGGCA	AGACTAGCCT	GGG	CAACATGGCA
chr5	32431884	32431906	hsa-mir-579	-	3	AGACTAGCCCGGCCAACATGGTG	AGACTAGCCC	GGC	CAACATGGTG
chr5	32435698	32435720	hsa-mir-579	-	3	AGGCAGGCTTAAAGGACATGCTT	AGGCAGGCTT	AAA	GGACATGCTT
chr5	148417394	148417416	hsa-mir-584	-	3	ATACATGCTGCCAAAGCAAGCTC	ATACATGCTG	CCA	AAGCAAGCTC
chr6	135598676	135598698	hsa-mir-548a-2	+	3	GGCCTAGCCCAGCAGGCAAGCCC	GGCCTAGCCC	AGC	AGGCAAGCCC
chr7	30299586	30299608	hsa-mir-550-1	+	3	TGCCATGTTGGCCAGGCTAGTCT	TGCCATGTTG	GCC	AGGCTAGTCT
chr7	95692082	95692104	hsa-mir-591	-	3	GGGCTTGCTCCAAAAGCATGAGT	GGGCTTGCTC	CAA	AAGCATGAGT
chr7	129209744	129209766	hsa-mir-183	-	3	CGGCTTGTCCCTCTGGCTTGCTC	CGGCTTGTCC	CTC	TGGCTTGCTC
chr7	129209744	129209766	hsa-mir-96	-	3	CGGCTTGTCCCTCTGGCTTGCTC	CGGCTTGTCC	CTC	TGGCTTGCTC
chr7	157056335	157056357	hsa-mir-153-2	-	3	AAGCAAGGTGGCAGGACATGCTC	AAGCAAGGTG	GCA	GGACATGCTC
chr7	157069136	157069158	hsa-mir-153-2	-	3	AGACTAGCCTCGCCAACATGGCA	AGACTAGCCT	CGC	CAACATGGCA
chr8	14754994	14755016	hsa-mir-383	-	3	AAGCATGCATATAAAACTTGTAC	AAGCATGCAT	ATA	AAACTTGTAC
chr8	22151347	22151369	hsa-mir-320	-	3	AGACTAGCCTGGGCAACATGGTG	AGACTAGCCT	GGG	CAACATGGTG
chr9	20702928	20702950	hsa-mir-491	+	3	CGCCATGTTGGCCAGGCTAGTCT	CGCCATGTTG	GCC	AGGCTAGTCT
chr9	130048197	130048219	hsa-mir-199b	-	3	TGGCTTGTATCCAGGACTTGCCT	TGGCTTGTAT	CCA	GGACTTGCCT
chrX	49663432	49663454	hsa-mir-188	+	3	TCACATGCCTAGTGAGCATGTTT	TCACATGCCT	AGT	GAGCATGTTT
chrX	49663432	49663454	hsa-mir-362	+	3	TCACATGCCTAGTGAGCATGTTT	TCACATGCCT	AGT	GAGCATGTTT
chrX	49663432	49663454	hsa-mir-500	+	3	TCACATGCCTAGTGAGCATGTTT	TCACATGCCT	AGT	GAGCATGTTT
chrX	49663432	49663454	hsa-mir-501	+	3	TCACATGCCTAGTGAGCATGTTT	TCACATGCCT	AGT	GAGCATGTTT
chrX	49663432	49663454	hsa-mir-502	+	3	TCACATGCCTAGTGAGCATGTTT	TCACATGCCT	AGT	GAGCATGTTT

chrX	49663432	49663454	hsa-mir-532	+	3	TCACATGCCTAGTGAGCATGTTT	TCACATGCCT	AGT	GAGCATGTTT
chrX	49663432	49663454	hsa-mir-660	+	3	TCACATGCCTAGTGAGCATGTTT	TCACATGCCT	AGT	GAGCATGTTT
chrX	53606328	53606350	hsa-let-7f-2	-	3	GGGCAAGCAGTAGTAACATGTTA	GGGCAAGCAG	TAG	TAACATGTTA
chrX	53606328	53606350	hsa-mir-98	-	3	GGGCAAGCAGTAGTAACATGTTA	GGGCAAGCAG	TAG	TAACATGTTA
chrX	53606755	53606777	hsa-let-7f-2	-	3	CAGCTAGCTAGGTGAGCTTGGCC	CAGCTAGCTA	GGT	GAGCTTGGCC
chrX	53606755	53606777	hsa-mir-98	-	3	CAGCTAGCTAGGTGAGCTTGGCC	CAGCTAGCTA	GGT	GAGCTTGGCC
chrX	73425753	73425775	hsa-mir-374	-	3	AGACAAGCCTGAGCAACATGGCA	AGACAAGCCT	GAG	CAACATGGCA
chrX	73425753	73425775	hsa-mir-545	-	3	AGACAAGCCTGAGCAACATGGCA	AGACAAGCCT	GAG	CAACATGGCA
chrX	138837226	138837248	hsa-mir-505	-	3	AGACTAGCCTGGCCAACATGGTG	AGACTAGCCT	GGC	CAACATGGTG
chr1	34903235	34903256	hsa-mir-552	-	2	AGGCTAGTGTCCAAGCTAGTTC	AGGCTAGTGT	CC	AAGCTAGTTC
chr1	170376606	170376627	hsa-mir-199a-2	-	2	GAACCTGCCCCTAAACTTGTTA	GAACCTGCCC	CT	AAACTTGTTA
chr1	170376606	170376627	hsa-mir-214	-	2	GAACCTGCCCCTAAACTTGTTA	GAACCTGCCC	CT	AAACTTGTTA
chr1	203683375	203683396	hsa-mir-135b	-	2	AGGCAAGCTGAGTGACAAGCAG	AGGCAAGCTG	AG	TGACAAGCAG
chr10	91337624	91337645	hsa-mir-107	-	2	AAGCAAGCTAATTAACATGTAT	AAGCAAGCTA	AT	TAACATGTAT
chr11	567724	567745	hsa-mir-210	-	2	GGACTTGGCTGCAGGCTTGTTC	GGACTTGGCT	GC	AGGCTTGTTC
chr11	43567965	43567986	hsa-mir-129-2	+	2	CTGCATGCCCTTGAGCAAGTTA	CTGCATGCCC	TT	GAGCAAGTTA
chr11	74727669	74727690	hsa-mir-326	-	2	AAACTTGCCTACAGACATGCAA	AAACTTGCCT	AC	AGACATGCAA
chr12	52723246	52723267	hsa-mir-615	+	2	AGGCATGCTGGGGAGCAAGTGC	AGGCATGCTG	GG	GAGCAAGTGC
chr12	61280132	61280153	hsa-let-7i	+	2	GGGCAAGTTAATTAACCTGTTT	GGGCAAGTTA	AT	TAACCTGTTT
chr12	94235528	94235549	hsa-mir-331	+	2	AGACATGCTCACATACATGCAT	AGACATGCTC	AC	ATACATGCAT
chr12	115066691	115066712	hsa-mir-620	-	2	AATCTTGGCTCAAAGCATGCTC	AATCTTGGCT	CA	AAGCATGCTC
chr12	115071096	115071117	hsa-mir-620	-	2	AAGCATGGCTCTGATCATGTTT	AAGCATGGCT	CT	GATCATGTTT
chr14	30557646	30557667	hsa-mir-624	-	2	GAGCTTGTTGATAAACTTGCAA	GAGCTTGTTG	AT	AAACTTGCAA
chr14	65004520	65004541	hsa-mir-625	+	2	TAACTTGTTTGTTGACAAGTTG	TAACTTGTTT	GT	TGACAAGTTG
chr14	99641218	99641239	hsa-mir-342	+	2	AATCATGCTACTTAGCATGTTT	AATCATGCTA	CT	TAGCATGTTT
chr16	55451015	55451036	hsa-mir-138-2	+	2	CACCATGCCCACTGACTAGTCT	CACCATGCCC	AC	TGACTAGTCT
chr17	53763829	53763850	hsa-mir-142	-	2	CAGCATGGCGCCAGACTTGCCT	CAGCATGGCG	CC	AGACTTGCCT
chr17	62220636	62220657	hsa-mir-634	+	2	TAGCTAGTTCATAGGCAAGCCA	TAGCTAGTTC	AT	AGGCAAGCCA
chr19	13855510	13855531	hsa-mir-181c	+	2	AACCTAGCTCAGAGGCAAGCCC	AACCTAGCTC	AG	AGGCAAGCCC
chr19	13855510	13855531	hsa-mir-181d	+	2	AACCTAGCTCAGAGGCAAGCCC	AACCTAGCTC	AG	AGGCAAGCCC

chr2	176718981	176719002	hsa-mir-10b	+	2	AGACATGCCCACCAACTAGCCT	AGACATGCCC	AC	CAACTAGCCT
chr20	33051584	33051605	hsa-mir-499	+	2	ACTCTTGTCCATGAACTTGCTC	ACTCTTGTCC	AT	GAACTTGCTC
chr21	25872051	25872072	hsa-mir-155	+	2	AAGCTTGTCAAGATGCATGTTC	AAGCTTGTCA	AG	ATGCATGTTC
chr22	19714729	19714750	hsa-mir-649	-	2	GAACAAGGGCATGAGCTTGTCT	GAACAAGGGC	AT	GAGCTTGTCT
chr3	45700803	45700824	hsa-mir-565	-	2	AGACTTGGTCCACGGCAAGCCT	AGACTTGGTC	CA	CGGCAAGCCT
chr3	196914731	196914752	hsa-mir-570	+	2	CCACATGTCCCAGCACATGTCC	CCACATGTCC	CA	GCACATGTCC
chr7	126487449	126487470	hsa-mir-592	-	2	AGGCAAGTTTTCAAACAAGTTT	AGGCAAGTTT	TC	AAACAAGTTT
chr8	105560143	105560164	hsa-mir-548a-3	-	2	TGACTAGCCTATAGACAAGTTC	TGACTAGCCT	AT	AGACAAGTTC
chr8	141812891	141812912	hsa-mir-151	-	2	CAACATGCAATAAAGCATGTCC	CAACATGCAA	TA	AAGCATGTCC
chr9	4834119	4834140	hsa-mir-101-2	+	2	AAACAAGTATTTTGGCTAGTCT	AAACAAGTAT	TT	TGGCTAGTCT
chr9	110838805	110838826	hsa-mir-32	-	2	GAACATGTGTCAAAACTTGTAC	GAACATGTGT	CA	AAACTTGTAC
chrX	133125092	133125113	hsa-mir-106a	-	2	GGACTTGGTTTTTGGCTTGCTA	GGACTTGGTT	TT	TGGCTTGCTA
chrX	133125092	133125113	hsa-mir-18b	-	2	GGACTTGGTTTTTGGCTTGCTA	GGACTTGGTT	TT	TGGCTTGCTA
chrX	133125092	133125113	hsa-mir-19b-2	-	2	GGACTTGGTTTTTGGCTTGCTA	GGACTTGGTT	TT	TGGCTTGCTA
chrX	133125092	133125113	hsa-mir-20b	-	2	GGACTTGGTTTTTGGCTTGCTA	GGACTTGGTT	TT	TGGCTTGCTA
chrX	133125092	133125113	hsa-mir-363	-	2	GGACTTGGTTTTTGGCTTGCTA	GGACTTGGTT	TT	TGGCTTGCTA
chrX	133125092	133125113	hsa-mir-92-2	-	2	GGACTTGGTTTTTGGCTTGCTA	GGACTTGGTT	TT	TGGCTTGCTA
chrX	137580389	137580410	hsa-mir-504	-	2	AAGCAAGTTACAGAACAGGTCA	AAGCAAGTTA	CA	GAACAGGTCA
chrX	146159460	146159481	hsa-mir-509	-	2	AGGCCAGCTCAGAGGCATGTCC	AGGCCAGCTC	AG	AGGCATGTCC
chrX	146159460	146159481	hsa-mir-510	-	2	AGGCCAGCTCAGAGGCATGTCC	AGGCCAGCTC	AG	AGGCATGTCC
chrX	146159460	146159481	hsa-mir-514-1	-	2	AGGCCAGCTCAGAGGCATGTCC	AGGCCAGCTC	AG	AGGCATGTCC
chr1	1084597	1084617	hsa-mir-200a	+	1	GAGCAAGTCTGGGCCATGCTG	GAGCAAGTCT	G	GGCCATGCTG
chr1	1084597	1084617	hsa-mir-200b	+	1	GAGCAAGTCTGGGCCATGCTG	GAGCAAGTCT	G	GGCCATGCTG
chr1	1084597	1084617	hsa-mir-429	+	1	GAGCAAGTCTGGGCCATGCTG	GAGCAAGTCT	G	GGCCATGCTG
chr1	34908417	34908437	hsa-mir-552	-	1	CAACTTGTCTATAGCTTGCTT	CAACTTGTCT	A	TAGCTTGCTT
chr11	74723261	74723281	hsa-mir-326	-	1	GGACAAGCCTGGGGCTAGACC	GGACAAGCCT	G	GGGCTAGACC
chr12	6934052	6934072	hsa-mir-141	+	1	TACCTAGCTCTCAGCATGTTT	TACCTAGCTC	T	CAGCATGTTT
chr12	6934052	6934072	hsa-mir-200c	+	1	TACCTAGCTCTCAGCATGTTT	TACCTAGCTC	T	CAGCATGTTT
chr12	79741130	79741150	hsa-mir-617	-	1	AAACAGGGCTCAAACATGCCC	AAACAGGGCT	C	AAACATGCCC
chr12	79754277	79754297	hsa-mir-617	-	1	AAACTAGCTCCTGGCATGGTG	AAACTAGCTC	C	TGGCATGGTG
chr12	107754837	107754857	hsa-mir-619	-	1	AAACATGTCCAGGTCATGGTC	AAACATGTCC	A	GGTCATGGTC
chr14	65004509	65004529	hsa-mir-625	+	1	AATCTTGCCCTTAACTTGTTT	AATCTTGCCC	T	TAACTTGTTT
chr14	99645616	99645636	hsa-mir-342	+	1	GAACAAGCTCCCAGCTTGCGC	GAACAAGCTC	C	CAGCTTGCGC
chr15	87714562	87714582	hsa-mir-9-3	+	1	GAACTAGTCCTTCACTTGCCA	GAACTAGTCC	T	TCACTTGCCA
chr17	6867195	6867215	hsa-mir-195	-	1	GAGCTTGTGTGGAGCTTGCCA	GAGCTTGTGT	G	GAGCTTGCCA
chr17	6867195	6867215	hsa-mir-497	-	1	GAGCTTGTGTGGAGCTTGCCA	GAGCTTGTGT	G	GAGCTTGCCA
chr17	62894812	62894832	hsa-mir-548d-2	-	1	AGGCATGTTTGAGACATGGTG	AGGCATGTTT	G	AGACATGGTG
chr19	19416092	19416112	hsa-mir-640	+	1	GGACTTGCACATGACTTGTTT	GGACTTGCAC	A	TGACTTGTTT
chr2	47458430	47458450	hsa-mir-559	+	1	GAACTAGTCCTTAACTAGTTA	GAACTAGTCC	T	TAACTAGTTA
chr2	176720035	176720055	hsa-mir-10b	+	1	TGGCAAGTCCCAGCCATGCCT	TGGCAAGTCC	C	AGCCATGCCT
chr20	48626478	48626498	hsa-mir-645	+	1	AAACTTGCATTTGGCATGCTT	AAACTTGCAT	T	TGGCATGCTT
chr20	61287036	61287056	hsa-mir-124a-3	+	1	ACACATGTTCGGTGCATGCCC	ACACATGTTC	G	GTGCATGCCC
chr20	61287565	61287585	hsa-mir-124a-3	+	1	ACACATGTTCGGCACATGCCC	ACACATGTTC	G	GCACATGCCC
chr21	36018783	36018803	hsa-mir-802	+	1	TGGCAAGTCCTCACCATGTCT	TGGCAAGTCC	T	CACCATGTCT
chr22	21502508	21502528	hsa-mir-650	+	1	AAGCAAGCTGGAAGCTTGCAT	AAGCAAGCTG	G	AAGCTTGCAT
chr22	36579444	36579464	hsa-mir-658	-	1	GGGCATGGTGGCAGCATGCCT	GGGCATGGTG	G	CAGCATGCCT

chr22	36579444	36579464	hsa-mir-659	-	1	GGGCATGGTGGCAGCATGCCT	GGGCATGGTG	G	CAGCATGCCT
chr3	44883378	44883398	hsa-mir-564	+	1	CAACATGTTTTGTACTTGCTC	CAACATGTTT	T	GTACTTGCTC
chr3	45701348	45701368	hsa-mir-565	-	1	GTGCAAGCTTCTAGCTTGCTT	GTGCAAGCTT	C	TAGCTTGCTT
chr3	50183988	50184008	hsa-mir-566	+	1	GGGCATGACCGAGCCATGCCC	GGGCATGACC	G	AGCCATGCCC
chr3	169752721	169752741	hsa-mir-551b	+	1	GCCCTTGCTCTGGGCTAGTTC	GCCCTTGCTC	T	GGGCTAGTTC
chr4	24137649	24137669	hsa-mir-573	-	1	AGACAAGTTTTCAACTTGTCT	AGACAAGTTT	T	CAACTTGTCT
chr4	115789173	115789193	hsa-mir-577	+	1	GAACAAGCTGTTGACATGTTT	GAACAAGCTG	T	TGACATGTTT
chr5	53289222	53289242	hsa-mir-581	-	1	TAACATGGCAAAAGCTTGTCT	TAACATGGCA	A	AAGCTTGTCT
chr7	25960235	25960255	hsa-mir-148a	-	1	TAGCAAGTTGTAAACTAGGTG	TAGCAAGTTG	T	AAACTAGGTG
chr7	27178451	27178471	hsa-mir-196b	-	1	TGGCTTGCTGAGAGCAAGCAG	TGGCTTGCTG	A	GAGCAAGCAG
chr7	92950051	92950071	hsa-mir-489	-	1	GAACTTGTTTGAAGCTGGTTC	GAACTTGTTT	G	AAGCTGGTTC
chr7	92950051	92950071	hsa-mir-653	-	1	GAACTTGTTTGAAGCTGGTTC	GAACTTGTTT	G	AAGCTGGTTC
chr7	158027169	158027189	hsa-mir-595	-	1	ACACTTGCTTAAAGCTTGCCG	ACACTTGCTT	A	AAGCTTGCCG
chr7	158027332	158027352	hsa-mir-595	-	1	TGGCTAGCATGAGGCTTGTTA	TGGCTAGCAT	G	AGGCTTGTTA
chr8	1750100	1750120	hsa-mir-596	+	1	AGACATGTTTCAGGCAAGCCA	AGACATGTTT	C	AGGCAAGCCA
chr8	1757482	1757502	hsa-mir-596	+	1	AAGCATGTTCTCAGCAAGTGC	AAGCATGTTC	T	CAGCAAGTGC
chr8	135882325	135882345	hsa-mir-30b	-	1	AGGCTAGTCACAAACAAGTGA	AGGCTAGTCA	C	AAACAAGTGA
chr8	135882325	135882345	hsa-mir-30d	-	1	AGGCTAGTCACAAACAAGTGA	AGGCTAGTCA	C	AAACAAGTGA
chr8	145095240	145095260	hsa-mir-661	-	1	AGGCAAGCCCCTAACAAGTCC	AGGCAAGCCC	C	TAACAAGTCC
chr9	125210769	125210789	hsa-mir-601	-	1	AAGCTAGCGCCAGGCTAGTCA	AAGCTAGCGC	C	AGGCTAGTCA
chr9	130049371	130049391	hsa-mir-199b	-	1	GAGCTGGCCTCAGGCATGTTG	GAGCTGGCCT	C	AGGCATGTTG
chrX	65153815	65153835	hsa-mir-223	+	1	AGACTTGCCCAAGCCATGATT	AGACTTGCCC	A	AGCCATGATT
chrX	133509307	133509327	hsa-mir-424	-	1	CAACAAGTCACATGCATGCCC	CAACAAGTCA	C	ATGCATGCCC
chrX	133509307	133509327	hsa-mir-450-1	-	1	CAACAAGTCACATGCATGCCC	CAACAAGTCA	C	ATGCATGCCC
chrX	133509307	133509327	hsa-mir-450-2	-	1	CAACAAGTCACATGCATGCCC	CAACAAGTCA	C	ATGCATGCCC
chrX	133509307	133509327	hsa-mir-503	-	1	CAACAAGTCACATGCATGCCC	CAACAAGTCA	C	ATGCATGCCC
chrX	133509307	133509327	hsa-mir-542	-	1	CAACAAGTCACATGCATGCCC	CAACAAGTCA	C	ATGCATGCCC
chrX	138841354	138841374	hsa-mir-505	-	1	AGGCAAGGCTGAGTCTTGCCC	AGGCAAGGCT	G	AGTCTTGCCC
chr1	149789108	149789127	hsa-mir-554	+	0	CAACATGCCTAGCCAAGTTT	CAACATGCCT		AGCCAAGTTT
chr10	134908638	134908657	hsa-mir-202	-	0	GGGCATGTCTTGGCAAGCCT	GGGCATGTCT		TGGCAAGCCT
chr12	93759345	93759364	hsa-mir-492	+	0	GAACAAGTCAAGGCTAGCTG	GAACAAGTCA		AGGCTAGCTG
chr13	89675718	89675737	hsa-mir-622	+	0	GGACAAGCTCCACCTTGCTA	GGACAAGCTC		CACCTTGCTA
chr15	77285829	77285848	hsa-mir-184	+	0	GGGCTAGCCTGAGCTTGCAG	GGGCTAGCCT		GAGCTTGCAG
chr15	86965541	86965560	hsa-mir-7-2	+	0	GGGCTTGCCCGGGCATGTGG	GGGCTTGCCC		GGGCATGTGG
chr16	15643104	15643123	hsa-mir-484	+	0	ACACAAGATTAGGCTTGTTC	ACACAAGATT		AGGCTTGTTC
chr16	55451027	55451046	hsa-mir-138-2	+	0	TGACTAGTCTTACCTAGCCC	TGACTAGTCT		TACCTAGCCC
chr17	25462037	25462056	hsa-mir-423	+	0	AGACAAGTTTCACCATGTTG	AGACAAGTTT		CACCATGTTG
chr19	4722866	4722885	hsa-mir-7-3	+	0	GGACAAGTGTGAACTTGTGC	GGACAAGTGT		GAACTTGTGC
chr19	19410794	19410813	hsa-mir-640	+	0	GCACTAGCCCACACTTGCTT	GCACTAGCCC		ACACTTGCTT
chr19	54687819	54687838	hsa-mir-150	-	0	AAGCTTGCTGGAGCAGGCCC	AAGCTTGCTG		GAGCAGGCCC
chr19	58934619	58934638	hsa-mir-516-4	+	0	TGGCATGTCCAACCTAGCCC	TGGCATGTCC		AACCTAGCCC
chr19	58934619	58934638	hsa-mir-517c	+	0	TGGCATGTCCAACCTAGCCC	TGGCATGTCC		AACCTAGCCC
chr19	58934619	58934638	hsa-mir-518a-1	+	0	TGGCATGTCCAACCTAGCCC	TGGCATGTCC		AACCTAGCCC
chr19	58934619	58934638	hsa-mir-518a-2	+	0	TGGCATGTCCAACCTAGCCC	TGGCATGTCC		AACCTAGCCC
chr19	58934619	58934638	hsa-mir-518d	+	0	TGGCATGTCCAACCTAGCCC	TGGCATGTCC		AACCTAGCCC
chr19	58934619	58934638	hsa-mir-518e	+	0	TGGCATGTCCAACCTAGCCC	TGGCATGTCC		AACCTAGCCC

chr19	58934619	58934638	hsa-mir-520h	+	0	TGGCATGTCCAACCTAGCCC	TGGCATGTCC		AACCTAGCCC
chr19	58934619	58934638	hsa-mir-521-1	+	0	TGGCATGTCCAACCTAGCCC	TGGCATGTCC		AACCTAGCCC
chr3	35763045	35763064	hsa-mir-128b	+	0	AGTCATGTTTTAACTTGTTC	AGTCATGTTT		TAACTTGTTC
chr3	121595431	121595450	hsa-mir-198	-	0	AGGCAAGCTTCAACAAGCCG	AGGCAAGCTT		CAACAAGCCG
chr3	189893453	189893472	hsa-mir-28	+	0	AAGCCAGTTATAACTTGTTT	AAGCCAGTTA		TAACTTGTTT
chr4	8056822	8056841	hsa-mir-95	-	0	AGACAAGCAGAGACAAGCAC	AGACAAGCAG		AGACAAGCAC
chr4	115792084	115792103	hsa-mir-577	+	0	AAGCAAGAACAAACTAGTTT	AAGCAAGAAC		AAACTAGTTT
chr6	72134348	72134367	hsa-mir-30c-2	-	0	GGGCTTGTCTGGGCATGCCC	GGGCTTGTCT		GGGCATGCCC
chr7	129191509	129191528	hsa-mir-182	-	0	GAACAAGGTCAAACAAGGTC	GAACAAGGTC		AAACAAGGTC
chr8	22151406	22151425	hsa-mir-320	-	0	AGGCATGGTGCGGCATGCCT	AGGCATGGTG		CGGCATGCCT
chr8	141803956	141803975	hsa-mir-151	-	0	TGGCTTGTTTTGGCAAGTTC	TGGCTTGTTT		TGGCAAGTTC
chrX	45484890	45484909	hsa-mir-221	-	0	GAACATGCATGCACATGTTT	GAACATGCAT		GCACATGTTT
chrX	45484890	45484909	hsa-mir-222	-	0	GAACATGCATGCACATGTTT	GAACATGCAT		GCACATGTTT
chrX	49647439	49647458	hsa-mir-188	+	0	AATCATGCCCAAGCTTGATT	AATCATGCCC		AAGCTTGATT
chrX	49647439	49647458	hsa-mir-532	+	0	AATCATGCCCAAGCTTGATT	AATCATGCCC		AAGCTTGATT
chrX	73422709	73422728	hsa-mir-374	-	0	AAACATGTCTTAGCTGGCTT	AAACATGTCT		TAGCTGGCTT
chrX	73422709	73422728	hsa-mir-545	-	0	AAACATGTCTTAGCTGGCTT	AAACATGTCT		TAGCTGGCTT
chrX	85045610	85045629	hsa-mir-361	-	0	AGACATGTTTAAGCAAGTGA	AGACATGTTT		AAGCAAGTGA
