# Supplementary material for: Dissecting microregulation of a master regulatory network
Source: BMC Genomics. 2008 Feb 23;9:88. doi: 10.1186/1471-2164-9-88 (PMC2289817; doi:10.1186/1471-2164-9-88)
Supplement: Additional File 3 — Putative miRNAs regulating known 23 upstream regulators and 48 downstream TFs of p53. For the known 23 upstream regulators and 48 downstream TFs of p53 (based on p53 Knowledgebase; ), putative miRs regulating them were extracted using MAMI server and the database . [file 1471-2164-9-88-S3.pdf]

**Additional File 3**: For the known 23 upstream regulators and 48 downstream TFs of p53 (based on p53 Knowledgebase; http://p53.bii.a-star.edu.sg), putative miRs regulating them were extracted using MAMI server and the database (http://mami.med.harvard.edu/) which has compilation of microRNA targets from five target prediction tools namely, DIANA-microT, miRanda, TargetScanS, miRtarget and PicTar. The last two columns indicate the role of the transcription factor in the p53 network and whether it is upstream or downstream to the p53 network.

| *p53-miR* | TargetGene | MAMI_score | TargetScanS | miRanda | microT | miRtarget | picTar | Role in p53 network | Upstream/Downstream to p53 |
|---|---|---|---|---|---|---|---|---|---|
| hsa-miR-124a | EGR1 | 0.11277 | 0.87 | -1 | -2 | -1 | -2 | Activator | Upstream |
| hsa-miR-181c | EGR1 | 0.11277 | 0.932 | -1 | -2 | -1 | -1 | Activator | Upstream |
| hsa-miR-183 | EGR1 | 0.35292 | 0.81 | 0.99798 | -2 | 0.0073 | -2 | Activator | Upstream |
| hsa-miR-192 | EGR1 | 0.14584 | -1 | -1 | -2 | 0.00365 | -1 | Activator | Upstream |
| hsa-miR-203 | EGR1 | 0.11277 | 0.53 | -1 | -2 | -1 | -2 | Activator | Upstream |
| hsa-miR-23a | EGR1 | 0.11277 | 0.56 | -1 | -2 | -1 | -1 | Activator | Upstream |
| hsa-miR-518b | EGR1 | 0.09432 | -2 | 0.9272 | -2 | -1 | -2 | Activator | Upstream |
| hsa-miR-518c | EGR1 | 0.09432 | -2 | 0.9272 | -2 | -1 | -2 | Activator | Upstream |
| hsa-miR-518d | EGR1 | 0.09432 | -2 | 0.9272 | -2 | -1 | -2 | Activator | Upstream |
| hsa-miR-124a | SP1 | 0.11277 | 0.962 | -2 | -2 | -2 | -2 | Activator | Upstream |
| hsa-miR-125b | SP1 | 0.11277 | 0.71 | -2 | -2 | -2 | -2 | Activator | Upstream |
| hsa-miR-128b | SP1 | 0.11277 | 0.939 | -2 | -2 | -2 | -2 | Activator | Upstream |
| hsa-miR-135a | SP1 | 0.11277 | 0.74 | -2 | -2 | -2 | -2 | Activator | Upstream |
| hsa-miR-135b | SP1 | 0.11277 | 0.74 | -2 | -2 | -2 | -2 | Activator | Upstream |
| hsa-miR-24 | SP1 | 0.11277 | 0.6 | -2 | -2 | -2 | -2 | Activator | Upstream |
| hsa-miR-33 | SP1 | 0.11277 | 0.55 | -2 | -2 | -2 | -2 | Activator | Upstream |
| hsa-miR-196a | HOXA5 | 0.11277 | 0.82 | -1 | -1 | -1 | -2 | Activator | Upstream |
| hsa-miR-196b | HOXA5 | 0.11277 | 0.82 | -1 | -1 | -1 | -2 | Activator | Upstream |
| hsa-miR-26b | HOXA5 | 0.2586 | 0.82 | -1 | -1 | 0.0219 | -2 | Activator | Upstream |
| hsa-miR-27a | HOXA5 | 0.11277 | 0.64 | -1 | -1 | -1 | -2 | Activator | Upstream |
| hsa-miR-376a | HOXA5 | 0.50875 | -2 | 0.99778 | 0.22562 | -1 | -2 | Activator | Upstream |
| hsa-miR-376b | HOXA5 | 0.50875 | -2 | 0.99778 | 0.18138 | -1 | -2 | Activator | Upstream |
| hsa-miR-484 | HOXA5 | 0.14584 | -2 | -2 | -1 | 0.01095 | -2 | Activator | Upstream |
| hsa-miR-96 | HOXA5 | 0.2586 | 0.77 | -1 | -1 | 0.0146 | -2 | Activator | Upstream |
| hsa-miR-101 | YY1 | 0.09432 | -1 | 0.70774 | -2 | -2 | -1 | Activator | Upstream |
| hsa-miR-181c | YY1 | 0.11277 | 0.88 | -1 | -1 | -2 | -1 | Activator | Upstream |
| hsa-miR-200a | YY1 | 0.11277 | 0.48 | -1 | -1 | -2 | -2 | Activator | Upstream |
| hsa-miR-29a | YY1 | 0.41443 | -2 | -1 | 0.37604 | -2 | -1 | Activator | Upstream |
| hsa-miR-34a | YY1 | 0.11277 | 0.65 | -1 | -1 | -2 | -2 | Activator | Upstream |
| hsa-miR-384 | YY1 | 0.09432 | -2 | 0.51585 | -1 | -2 | -2 | Activator | Upstream |
| hsa-miR-511 | YY1 | 0.09432 | -2 | 0.64718 | -2 | -2 | -2 | Activator | Upstream |
| hsa-miR-346 | NFIC | 0.09432 | -2 | 0.85331 | -2 | -2 | -2 | Activator | Upstream |
| hsa-miR-30a-3p | NFKB1 | 0.09432 | -2 | 0.53885 | -2 | -2 | -1 | Activator | Upstream |
| hsa-miR-508 | NFKB1 | 0.09432 | -2 | 0.79507 | -2 | -2 | -2 | Activator | Upstream |
| hsa-miR-9 | NFKB1 | 0.11277 | 0.81 | -2 | -2 | -2 | -2 | Activator | Upstream |
| hsa-miR-125b | USF1 | 0.09432 | -2 | 0.10515 | -2 | -2 | -2 | Activator | Upstream |
| hsa-miR-296 | USF1 | 0.09432 | -2 | 0.73209 | -2 | -2 | -2 | Activator | Upstream |
| hsa-let-7a | MYC | 0.09432 | -2 | 0.98208 | -2 | -2 | -2 | Activator | Upstream |
| hsa-let-7b | MYC | 0.09432 | -2 | 0.98208 | -2 | -2 | -2 | Activator | Upstream |
| hsa-let-7f | MYC | 0.09432 | -2 | 0.98208 | -2 | -2 | -2 | Activator | Upstream |
| hsa-let-7i | MYC | 0.09432 | -2 | 0.98208 | -2 | -2 | -2 | Activator | Upstream |
| hsa-miR-155 | MYC | 0.09432 | -2 | 0.21942 | -2 | -2 | -2 | Activator | Upstream |
| hsa-miR-196a | MYC | 0.09432 | -2 | 0.98528 | -2 | -2 | -2 | Activator | Upstream |
| hsa-miR-196b | MYC | 0.09432 | -2 | 0.98528 | -2 | -2 | -2 | Activator | Upstream |
| hsa-miR-200b | MYC | 0.09432 | -2 | 0.37261 | -2 | -2 | -2 | Activator | Upstream |
| hsa-miR-200c | MYC | 0.09432 | -2 | 0.37261 | -2 | -2 | -2 | Activator | Upstream |
| hsa-miR-429 | MYC | 0.09432 | -2 | 0.02147 | -2 | -2 | -2 | Activator | Upstream |
| hsa-miR-98 | MYC | 0.09432 | -2 | 0.9952 | -2 | -2 | -2 | Activator | Upstream |
| hsa-miR-33 | MAX | 0.11277 | 0.51 | -2 | -1 | -2 | -1 | Activator | Upstream |
| hsa-miR-200a | JUN | 0.11277 | 0.53 | -1 | -1 | -2 | -2 | Cell-type specific activator/ repressor | Upstream |
| hsa-miR-200b | JUN | 0.41443 | -1 | -1 | 0.17254 | -2 | -1 | Cell-type specific activator/ repressor | Upstream |
| hsa-miR-32 | JUN | 0.11277 | 0.85 | -1 | -1 | -2 | -2 | Cell-type specific activator/ repressor | Upstream |
| hsa-miR-495 | JUN | 0.09432 | -2 | 0.93878 | -2 | -2 | -2 | Cell-type specific activator/ repressor | Upstream |
| hsa-miR-92 | JUN | 0.11277 | 0.85 | -1 | -1 | -2 | -2 | Cell-type specific activator/ repressor | Upstream |
| hsa-miR-101 | FOS | 0.35292 | 0.86 | 0.99887 | -2 | 0.01095 | -2 | Cell-type specific activator/ repressor | Upstream |
| hsa-miR-181c | FOS | 0.35292 | 0.63 | 0.99588 | -1 | 0.01095 | -2 | Cell-type specific activator/ repressor | Upstream |
| hsa-miR-221 | FOS | 0.11277 | 0.8 | -1 | -1 | -1 | -2 | Cell-type specific activator/ repressor | Upstream |
| hsa-miR-222 | FOS | 0.20708 | 0.8 | 0.8804 | -1 | -1 | -2 | Cell-type specific activator/ repressor | Upstream |
| hsa-miR-29a | FOS | 0.09432 | -2 | 0.97906 | -1 | -1 | -1 | Cell-type specific activator/ repressor | Upstream |
| hsa-miR-29b | FOS | 0.09432 | -2 | 0.97906 | -1 | -1 | -2 | Cell-type specific activator/ repressor | Upstream |
| hsa-miR-29c | FOS | 0.09432 | -2 | 0.97906 | -1 | -1 | -2 | Cell-type specific activator/ repressor | Upstream |
| hsa-miR-509 | FOS | 0.09432 | -2 | 0.13106 | -2 | -1 | -2 | Cell-type specific activator/ repressor | Upstream |
| hsa-miR-7 | FOS | 0.09432 | -1 | 0.95869 | -1 | -1 | -1 | Cell-type specific activator/ repressor | Upstream |
| hsa-miR-106a | BCL6 | 0.11277 | 0.49 | -1 | -2 | -2 | -2 | Repressor | Upstream |
| hsa-miR-10a | BCL6 | 0.20708 | 0.66 | 0.62379 | -2 | -2 | -2 | Repressor | Upstream |
| hsa-miR-10b | BCL6 | 0.11277 | 0.66 | -1 | -2 | -2 | -2 | Repressor | Upstream |
| hsa-miR-124a | BCL6 | 0.11277 | 0.85 | -1 | -2 | -2 | -1 | Repressor | Upstream |
| hsa-miR-129 | BCL6 | 0.09432 | -2 | 0.93663 | -2 | -2 | -2 | Repressor | Upstream |
| hsa-miR-181c | BCL6 | 0.11277 | 0.907 | -1 | -2 | -2 | -2 | Repressor | Upstream |
| hsa-miR-183 | BCL6 | 0.11277 | 0.6 | -1 | -2 | -2 | -2 | Repressor | Upstream |
| hsa-miR-20b | BCL6 | 0.11277 | 0.49 | -1 | -2 | -2 | -2 | Repressor | Upstream |
| hsa-miR-26b | BCL6 | 0.11277 | 0.64 | -1 | -2 | -2 | -2 | Repressor | Upstream |
| hsa-miR-30b | BCL6 | 0.11277 | 0.901 | -1 | -2 | -2 | -2 | Repressor | Upstream |
| hsa-miR-30c | BCL6 | 0.11277 | 0.901 | -1 | -2 | -2 | -2 | Repressor | Upstream |
| hsa-miR-30d | BCL6 | 0.11277 | 0.901 | -1 | -2 | -2 | -1 | Repressor | Upstream |
| hsa-miR-346 | BCL6 | 0.09432 | -2 | 0.72746 | -2 | -2 | -2 | Repressor | Upstream |
| hsa-miR-34a | BCL6 | 0.11277 | 0.67 | -1 | -2 | -2 | -2 | Repressor | Upstream |
| hsa-miR-372 | BCL6 | 0.11277 | 0.68 | -1 | -2 | -2 | -1 | Repressor | Upstream |
| hsa-miR-373 | BCL6 | 0.11277 | 0.68 | -1 | -2 | -2 | -2 | Repressor | Upstream |
| hsa-miR-520b | BCL6 | 0.09432 | -2 | 0.5253 | -2 | -2 | -2 | Repressor | Upstream |
| hsa-miR-520c | BCL6 | 0.09432 | -2 | 0.5253 | -2 | -2 | -2 | Repressor | Upstream |
| hsa-miR-520d | BCL6 | 0.09432 | -2 | 0.5253 | -2 | -2 | -2 | Repressor | Upstream |
| hsa-miR-520f | BCL6 | 0.09432 | -2 | 0.5253 | -2 | -2 | -2 | Repressor | Upstream |
| hsa-miR-9 | BCL6 | 0.11277 | 0.79 | -2 | -2 | -2 | -2 | Repressor | Upstream |
| hsa-miR-135a | ATF3 | 0.11277 | 0.78 | -1 | -2 | -1 | -2 | Repressor | Upstream |

| | | | | | | | | | |
|---|---|---|---|---|---|---|---|---|---|
| hsa-miR-135b | ATF3 | 0.11277 | 0.78 | -1 | -2 | -1 | -2 | Repressor | Upstream |
| hsa-miR-192 | ATF3 | 0.09432 | -1 | 0.69078 | -2 | -1 | -1 | Repressor | Upstream |
| hsa-miR-30d | ATF3 | 0.09432 | -1 | 0.89288 | -2 | -1 | -1 | Repressor | Upstream |
| hsa-miR-107 | KLF4 | 0.11277 | 0.57 | -1 | -1 | -1 | -1 | Repressor | Upstream |
| hsa-miR-10a | KLF4 | 0.11277 | 0.52 | -1 | -1 | -1 | -2 | Repressor | Upstream |
| hsa-miR-10b | KLF4 | 0.11277 | 0.52 | -1 | -1 | -1 | -2 | Repressor | Upstream |
| hsa-miR-124a | KLF4 | 0.2586 | 0.78 | -1 | -1 | 0.00365 | -2 | Repressor | Upstream |
| hsa-miR-128b | KLF4 | 0.11277 | 0.77 | -1 | -1 | -1 | -2 | Repressor | Upstream |
| hsa-miR-135a | KLF4 | 0.11277 | 0.71 | -1 | -1 | -1 | -2 | Repressor | Upstream |
| hsa-miR-135b | KLF4 | 0.11277 | 0.71 | -1 | -1 | -1 | -2 | Repressor | Upstream |
| hsa-miR-148a | KLF4 | 0.76735 | 0.76 | 0.80599 | 0.33622 | 0.0073 | -2 | Repressor | Upstream |
| hsa-miR-200b | KLF4 | 0.11277 | 0.68 | -1 | -1 | -1 | -1 | Repressor | Upstream |
| hsa-miR-200c | KLF4 | 0.11277 | 0.68 | -1 | -1 | -1 | -2 | Repressor | Upstream |
| hsa-miR-26b | KLF4 | 0.11277 | 0.69 | -1 | -1 | -1 | -2 | Repressor | Upstream |
| hsa-miR-32 | KLF4 | 0.35292 | 0.982 | 0.92963 | -1 | 0.01095 | -2 | Repressor | Upstream |
| hsa-miR-34a | KLF4 | 0.2586 | 0.68 | -1 | -1 | 0.00365 | -2 | Repressor | Upstream |
| hsa-miR-449 | KLF4 | 0.14584 | -2 | -1 | -1 | 0.01825 | -2 | Repressor | Upstream |
| hsa-miR-508 | KLF4 | 0.24015 | -2 | 0.48935 | -2 | 0.00001 | -2 | Repressor | Upstream |
| hsa-miR-7 | KLF4 | 0.2586 | 0.87 | -1 | -1 | 0.0876 | -2 | Repressor | Upstream |
| hsa-miR-92 | KLF4 | 0.11277 | 0.982 | -1 | -1 | -1 | -2 | Repressor | Upstream |
| hsa-miR-153 | PAX2 | 0.11277 | 0.85 | -1 | -2 | -2 | -2 | Repressor | Upstream |
| hsa-miR-138 | PAX8 | 0.09432 | -1 | 0.15986 | -2 | -1 | -1 | Repressor | Upstream |
| hsa-miR-326 | PAX8 | 0.14584 | -2 | -1 | -2 | 0.0292 | -2 | Repressor | Upstream |
| hsa-miR-124a | ETS1 | 0.11277 | 0.83 | -1 | -2 | -1 | -2 | Uncharacterized | Upstream |
| hsa-miR-125b | ETS1 | 0.11277 | 0.84 | -1 | -2 | -1 | -2 | Uncharacterized | Upstream |
| hsa-miR-155 | ETS1 | 0.11277 | 0.7 | -1 | -2 | -1 | -2 | Uncharacterized | Upstream |
| hsa-miR-181c | ETS1 | 0.11277 | 0.79 | -1 | -2 | -1 | -2 | Uncharacterized | Upstream |
| hsa-miR-199a | ETS1 | 0.11277 | 0.89 | -2 | -2 | -1 | -2 | Uncharacterized | Upstream |
| hsa-miR-199b | ETS1 | 0.11277 | 0.89 | -1 | -2 | -1 | -2 | Uncharacterized | Upstream |
| hsa-miR-200b | ETS1 | 0.11277 | 0.78 | -1 | -2 | -1 | -1 | Uncharacterized | Upstream |
| hsa-miR-200c | ETS1 | 0.11277 | 0.78 | -1 | -2 | -1 | -1 | Uncharacterized | Upstream |
| hsa-miR-214 | ETS1 | 0.14584 | -2 | -1 | -2 | 0.0073 | -1 | Uncharacterized | Upstream |
| hsa-miR-221 | ETS1 | 0.11277 | 0.74 | -1 | -2 | -1 | -2 | Uncharacterized | Upstream |
| hsa-miR-222 | ETS1 | 0.11277 | 0.74 | -1 | -2 | -1 | -2 | Uncharacterized | Upstream |
| hsa-miR-9 | ETS1 | 0.11277 | 0.85 | -2 | -2 | -1 | -2 | Uncharacterized | Upstream |
| hsa-miR-199a | ETS2 | 0.11277 | 0.78 | -2 | -2 | -2 | -1 | Uncharacterized | Upstream |
| hsa-miR-199b | ETS2 | 0.11277 | 0.78 | -1 | -2 | -2 | -1 | Uncharacterized | Upstream |
| hsa-miR-203 | ETS2 | 0.11277 | 0.68 | -1 | -2 | -2 | -2 | Uncharacterized | Upstream |
| hsa-miR-221 | ETS2 | 0.11277 | 0.906 | -1 | -2 | -2 | -2 | Uncharacterized | Upstream |
| hsa-miR-222 | ETS2 | 0.11277 | 0.906 | -1 | -2 | -2 | -2 | Uncharacterized | Upstream |
| hsa-miR-155 | CEBPB | 0.62151 | 0.78 | 0.71117 | 0.24774 | -1 | -2 | Uncharacterized | Upstream |
| hsa-miR-342 | CEBPB | 0.09432 | -2 | 0.80788 | -1 | -1 | -1 | Uncharacterized | Upstream |
| hsa-miR-510 | CEBPB | 0.14584 | -2 | -1 | -2 | 0.01825 | -2 | Uncharacterized | Upstream |
| hsa-miR-29b | BAX | 0.09432 | -2 | 0.16613 | -2 | -2 | -2 | activator | Downstream |
| hsa-miR-29c | BAX | 0.09432 | -2 | 0.16613 | -2 | -2 | -2 | activator | Downstream |
| hsa-miR-148a | BBC3 | 0.09432 | -2 | 0.72848 | -2 | -2 | -2 | activator | Downstream |
| hsa-miR-296 | BBC3 | 0.09432 | -2 | 0.8104 | -2 | -2 | -2 | activator | Downstream |
| hsa-miR-210 | BDKRB2 | 0.09432 | -2 | 0.37001 | -2 | -2 | -2 | activator | Downstream |
| hsa-let-7a | BTG2 | 0.11277 | 0.84 | -1 | -1 | -2 | -2 | activator | Downstream |
| hsa-let-7b | BTG2 | 0.11277 | 0.84 | -1 | -1 | -2 | -2 | activator | Downstream |
| hsa-let-7f | BTG2 | 0.11277 | 0.84 | -1 | -1 | -2 | -2 | activator | Downstream |
| hsa-let-7i | BTG2 | 0.11277 | 0.84 | -1 | -1 | -2 | -2 | activator | Downstream |
| hsa-miR-106a | BTG2 | 0.11277 | 0.56 | -1 | -1 | -2 | -2 | activator | Downstream |
| hsa-miR-107 | BTG2 | 0.11277 | 0.72 | -1 | -1 | -2 | -2 | activator | Downstream |
| hsa-miR-124a | BTG2 | 0.11277 | 0.84 | -1 | -1 | -2 | -1 | activator | Downstream |
| hsa-miR-128b | BTG2 | 0.11277 | 0.72 | -1 | -1 | -2 | -2 | activator | Downstream |
| hsa-miR-195 | BTG2 | 0.11277 | 0.92 | -1 | -1 | -2 | -2 | activator | Downstream |
| hsa-miR-20b | BTG2 | 0.11277 | 0.56 | -1 | -2 | -2 | -2 | activator | Downstream |
| hsa-miR-27a | BTG2 | 0.62151 | 0.963 | 0.86604 | 0.4424 | -2 | -2 | activator | Downstream |
| hsa-miR-32 | BTG2 | 0.20708 | 0.988 | 0.987 | -1 | -2 | -2 | activator | Downstream |
| hsa-miR-9 | BTG2 | 0.11277 | 0.77 | -2 | -1 | -2 | -2 | activator | Downstream |
| hsa-miR-92 | BTG2 | 0.11277 | 0.988 | -1 | -1 | -2 | -2 | activator | Downstream |
| hsa-miR-98 | BTG2 | 0.11277 | 0.84 | -1 | -1 | -2 | -2 | activator | Downstream |
| hsa-miR-122a | CCNG1 | 0.35292 | 0.7 | 0.96174 | -2 | 0.00365 | -2 | activator | Downstream |
| hsa-miR-181d | CCNG1 | 0.14584 | -2 | -1 | -2 | 0.0073 | -2 | activator | Downstream |
| hsa-miR-203 | CCNG1 | 0.11277 | 0.63 | -1 | -2 | -1 | -2 | activator | Downstream |
| hsa-miR-23a | CCNG1 | 0.11277 | 0.75 | -1 | -2 | -1 | -2 | activator | Downstream |
| hsa-miR-27a | CCNG1 | 0.2586 | 0.82 | -1 | -2 | 0.00001 | -2 | activator | Downstream |
| hsa-miR-510 | CCNG1 | 0.14584 | -2 | -1 | -2 | 0.0073 | -2 | activator | Downstream |
| hsa-miR-9 | CCNG1 | 0.11277 | 0.86 | -2 | -2 | -1 | -2 | activator | Downstream |
| hsa-miR-106a | CDKN1A | 0.11277 | 0.82 | -1 | -2 | -2 | -2 | activator | Downstream |
| hsa-miR-124a | CDKN1A | 0.09432 | -1 | 0.88139 | -2 | -2 | -2 | activator | Downstream |
| hsa-miR-20b | CDKN1A | 0.11277 | 0.82 | -1 | -2 | -2 | -2 | activator | Downstream |
| hsa-miR-296 | CDKN1A | 0.09432 | -2 | 0.80043 | -2 | -2 | -2 | activator | Downstream |
| hsa-miR-24 | CTSD | 0.09432 | -2 | 0.86131 | -2 | -2 | -2 | activator | Downstream |
| hsa-miR-326 | CTSD | 0.09432 | -2 | 0.57473 | -2 | -2 | -2 | activator | Downstream |
| hsa-miR-331 | CTSD | 0.09432 | -2 | 0.45511 | -2 | -2 | -2 | activator | Downstream |
| hsa-miR-96 | CTSD | 0.09432 | -2 | 0.00085 | -2 | -2 | -2 | activator | Downstream |
| hsa-miR-195 | CX3CL1 | 0.11277 | 0.74 | -2 | -2 | -1 | -2 | activator | Downstream |
| hsa-miR-296 | CX3CL1 | 0.14584 | -2 | -2 | -2 | 0.04015 | -2 | activator | Downstream |
| hsa-miR-326 | CX3CL1 | 0.14584 | -2 | -2 | -2 | 0.05475 | -1 | activator | Downstream |
| hsa-miR-331 | CX3CL1 | 0.14584 | -2 | -2 | -2 | 0.03285 | -1 | activator | Downstream |
| hsa-miR-424 | CX3CL1 | 0.14584 | -2 | -2 | -2 | 0.00365 | -2 | activator | Downstream |
| hsa-miR-497 | CX3CL1 | 0.14584 | -2 | -2 | -2 | 0.00001 | -2 | activator | Downstream |
| hsa-miR-504 | CX3CL1 | 0.14584 | -2 | -2 | -2 | 0.0073 | -2 | activator | Downstream |
| hsa-miR-516-3p | CX3CL1 | 0.14584 | -2 | -2 | -2 | 0.1314 | -2 | activator | Downstream |
| hsa-miR-101 | DKK1 | 0.09432 | -1 | 0.7591 | -2 | -2 | -1 | activator | Downstream |
| hsa-miR-148a | DKK1 | 0.09432 | -1 | 0.63777 | -2 | -2 | -1 | activator | Downstream |
| hsa-miR-372 | DKK1 | 0.11277 | 0.69 | -1 | -2 | -2 | -1 | activator | Downstream |
| hsa-miR-373 | DKK1 | 0.11277 | 0.69 | -1 | -2 | -2 | -2 | activator | Downstream |
| hsa-miR-502 | DKK1 | 0.09432 | -2 | 0.13733 | -2 | -2 | -2 | activator | Downstream |

| | | | | | | | | | |
|---|---|---|---|---|---|---|---|---|---|
| hsa-miR-106a | EEF1A1 | 0.09432 | -1 | 0.90109 | -2 | -2 | -2 | activator | Downstream |
| hsa-miR-20b | EEF1A1 | 0.09432 | -1 | 0.91682 | -2 | -2 | -1 | activator | Downstream |
| hsa-miR-33 | EEF1A1 | 0.20708 | 0.59 | 0.99984 | -2 | -2 | -2 | activator | Downstream |
| hsa-miR-373 | EEF1A1 | 0.09432 | -1 | 0.04632 | -2 | -2 | -2 | activator | Downstream |
| hsa-miR-450 | EEF1A1 | 0.09432 | -2 | 1 | -2 | -2 | -2 | activator | Downstream |
| hsa-miR-519d | EEF1A1 | 0.09432 | -2 | 0.93204 | -2 | -2 | -2 | activator | Downstream |
| hsa-miR-520a | EEF1A1 | 0.09432 | -2 | 0.92994 | -2 | -2 | -2 | activator | Downstream |
| hsa-miR-520b | EEF1A1 | 0.09432 | -2 | 0.92994 | -2 | -2 | -2 | activator | Downstream |
| hsa-miR-520c | EEF1A1 | 0.09432 | -2 | 0.92994 | -2 | -2 | -2 | activator | Downstream |
| hsa-miR-520d | EEF1A1 | 0.09432 | -2 | 0.92994 | -2 | -2 | -2 | activator | Downstream |
| hsa-miR-520g | EEF1A1 | 0.09432 | -2 | 0.92994 | -2 | -2 | -2 | activator | Downstream |
| hsa-miR-520h | EEF1A1 | 0.09432 | -2 | 0.92994 | -2 | -2 | -2 | activator | Downstream |
| hsa-miR-128b | EGFR | 0.11277 | 0.78 | -1 | -2 | -2 | -2 | activator | Downstream |
| hsa-miR-7 | EGFR | 0.09432 | -1 | 0.33018 | -2 | -2 | -1 | activator | Downstream |
| hsa-let-7a | FAS | 0.14584 | -1 | -2 | -2 | 0.00001 | -2 | activator | Downstream |
| hsa-let-7b | FAS | 0.14584 | -1 | -2 | -2 | 0.00001 | -2 | activator | Downstream |
| hsa-let-7f | FAS | 0.14584 | -1 | -2 | -2 | 0.00001 | -2 | activator | Downstream |
| hsa-let-7i | FAS | 0.14584 | -1 | -2 | -2 | 0.00365 | -2 | activator | Downstream |
| hsa-miR-196a | FAS | 0.14584 | -1 | -2 | -2 | 0.01095 | -2 | activator | Downstream |
| hsa-miR-196b | FAS | 0.14584 | -1 | -2 | -2 | 0.01095 | -2 | activator | Downstream |
| hsa-miR-23a | FAS | 0.11277 | 0.73 | -2 | -2 | -1 | -2 | activator | Downstream |
| hsa-miR-98 | FAS | 0.14584 | -1 | -2 | -2 | 0.00001 | -2 | activator | Downstream |
| hsa-miR-148a | GADD45A | 0.11277 | 0.74 | -1 | -2 | -1 | -2 | activator | Downstream |
| hsa-miR-326 | GADD45A | 0.09432 | -2 | 0.3844 | -2 | -1 | -1 | activator | Downstream |
| hsa-miR-362 | GADD45A | 0.14584 | -2 | -1 | -2 | 0.00001 | -2 | activator | Downstream |
| hsa-miR-374 | GADD45A | 0.09432 | -2 | 0.90809 | -2 | -1 | -2 | activator | Downstream |
| hsa-miR-502 | GADD45A | 0.24015 | -2 | 0.85312 | -2 | 0.00365 | -2 | activator | Downstream |
| hsa-miR-19b | IGFBP3 | 0.20708 | 0.82 | 0.65905 | -1 | -1 | -2 | activator | Downstream |
| hsa-miR-34a | IGFBP3 | 0.11277 | 0.75 | -1 | -1 | -1 | -2 | activator | Downstream |
| hsa-miR-449 | IGFBP3 | 0.41443 | -2 | -1 | 0.34507 | -1 | -2 | activator | Downstream |
| hsa-miR-495 | IGFBP3 | 0.14584 | -2 | -1 | -2 | 0.00365 | -2 | activator | Downstream |
| hsa-miR-153 | MDM2 | 0.09432 | -2 | 0.89457 | -2 | -2 | -2 | activator | Downstream |
| hsa-miR-23a | MET | 0.11277 | 0.86 | -1 | -2 | -2 | -2 | activator | Downstream |
| hsa-miR-34a | MET | 0.11277 | 0.94 | -1 | -2 | -2 | -2 | activator | Downstream |
| hsa-miR-29a | MMP2 | 0.24015 | -2 | 0.96138 | -2 | 0.04745 | -1 | activator | Downstream |
| hsa-miR-29b | MMP2 | 0.24015 | -2 | 0.96138 | -2 | 0.04745 | -2 | activator | Downstream |
| hsa-miR-29c | MMP2 | 0.24015 | -2 | 0.96138 | -2 | 0.04745 | -2 | activator | Downstream |
| hsa-miR-519b | MMP2 | 0.14584 | -2 | -1 | -2 | 0.00365 | -2 | activator | Downstream |
| hsa-miR-519c | MMP2 | 0.09432 | -2 | 0.9005 | -2 | -1 | -2 | activator | Downstream |
| hsa-miR-520g | MMP2 | 0.14584 | -2 | -1 | -2 | 0.00001 | -2 | activator | Downstream |
| hsa-miR-520h | MMP2 | 0.14584 | -2 | -1 | -2 | 0.00001 | -2 | activator | Downstream |
| hsa-let-7i | PCBP4 | 0.09432 | -1 | 0.64792 | -2 | -2 | -2 | activator | Downstream |
| hsa-miR-195 | PCBP4 | 0.11277 | 0.72 | -1 | -2 | -2 | -2 | activator | Downstream |
| hsa-miR-296 | PCBP4 | 0.09432 | -2 | 0.82996 | -2 | -2 | -2 | activator | Downstream |
| hsa-miR-331 | PCBP4 | 0.09432 | -2 | 0.82314 | -2 | -2 | -2 | activator | Downstream |
| hsa-miR-128b | PLK2 | 0.58557 | 0.88 | 0.99318 | -1 | 0.01825 | 0.52516 | activator | Downstream |
| hsa-miR-200b | PLK2 | 0.35292 | 0.66 | 0.90382 | -1 | 0.01095 | -1 | activator | Downstream |
| hsa-miR-200c | PLK2 | 0.35292 | 0.66 | 0.90382 | -1 | 0.0146 | -1 | activator | Downstream |
| hsa-miR-214 | PLK2 | 0.14584 | -2 | -1 | -1 | 0.0365 | -1 | activator | Downstream |
| hsa-miR-27a | PLK2 | 0.58557 | 0.979 | 0.9921 | -1 | 0.2993 | 0.52516 | activator | Downstream |
| hsa-miR-320 | PLK2 | 0.14584 | -2 | -1 | -1 | 0.01825 | -1 | activator | Downstream |
| hsa-miR-500 | PLK2 | 0.09432 | -2 | 0.1563 | -2 | -1 | -2 | activator | Downstream |
| hsa-miR-516-3p | PLK2 | 0.14584 | -2 | -1 | -2 | 0.0073 | -2 | activator | Downstream |
| hsa-miR-520c | PLK2 | 0.09432 | -2 | 0.97028 | -2 | -1 | -2 | activator | Downstream |
| hsa-let-7a | RB1 | 0.11277 | 0.83 | -1 | -2 | -1 | -2 | activator | Downstream |
| hsa-let-7b | RB1 | 0.11277 | 0.83 | -1 | -2 | -1 | -2 | activator | Downstream |
| hsa-let-7f | RB1 | 0.11277 | 0.83 | -1 | -2 | -1 | -2 | activator | Downstream |
| hsa-let-7i | RB1 | 0.11277 | 0.83 | -1 | -2 | -1 | -2 | activator | Downstream |
| hsa-miR-106a | RB1 | 0.11277 | 0.8 | -1 | -2 | -1 | -2 | activator | Downstream |
| hsa-miR-192 | RB1 | 0.20708 | 0.8 | 0.85928 | -2 | -1 | -2 | activator | Downstream |
| hsa-miR-20b | RB1 | 0.11277 | 0.8 | -1 | -2 | -1 | -2 | activator | Downstream |
| hsa-miR-33 | RB1 | 0.11277 | 0.57 | -1 | -2 | -1 | -1 | activator | Downstream |
| hsa-miR-520g | RB1 | 0.09432 | -2 | 0.8472 | -2 | -1 | -2 | activator | Downstream |
| hsa-miR-520h | RB1 | 0.09432 | -2 | 0.8472 | -2 | -1 | -2 | activator | Downstream |
| hsa-miR-525 | RB1 | 0.14584 | -2 | -2 | -2 | 0.1533 | -2 | activator | Downstream |
| hsa-miR-7 | RB1 | 0.11277 | 0.51 | -1 | -2 | -1 | -2 | activator | Downstream |
| hsa-miR-98 | RB1 | 0.11277 | 0.83 | -1 | -2 | -1 | -2 | activator | Downstream |
| hsa-miR-95 | S100A2 | 0.09432 | -2 | 0.0489 | -2 | -2 | -2 | activator | Downstream |
| hsa-miR-181c | SERPINE1 | 0.09432 | -1 | 0.39391 | -1 | -1 | -1 | activator | Downstream |
| hsa-miR-181d | SERPINE1 | 0.09432 | -2 | 0.39391 | -2 | -1 | -2 | activator | Downstream |
| hsa-miR-30b | SERPINE1 | 0.76735 | 0.82 | 0.72721 | 0.43798 | 0.0292 | -2 | activator | Downstream |
| hsa-miR-30c | SERPINE1 | 0.76735 | 0.82 | 0.72721 | 0.5353 | 0.0292 | -2 | activator | Downstream |
| hsa-miR-30d | SERPINE1 | 0.11277 | 0.82 | -1 | -1 | -1 | -2 | activator | Downstream |
| hsa-miR-34a | SERPINE1 | 0.2586 | 0.75 | -1 | -1 | 0.04015 | -2 | activator | Downstream |
| hsa-miR-449 | SERPINE1 | 0.14584 | -2 | -1 | -1 | 0.04015 | -2 | activator | Downstream |
| hsa-let-7a | SFN | 0.09432 | -2 | 0.16363 | -2 | -2 | -2 | activator | Downstream |
| hsa-let-7b | SFN | 0.09432 | -2 | 0.16363 | -2 | -2 | -2 | activator | Downstream |
| hsa-miR-122a | SFN | 0.09432 | -2 | 0.65 | -2 | -2 | -2 | activator | Downstream |
| hsa-let-7a | TAP1 | 0.09432 | -2 | 0.10899 | -2 | -2 | -2 | activator | Downstream |
| hsa-let-7b | TAP1 | 0.09432 | -2 | 0.10899 | -2 | -2 | -2 | activator | Downstream |
| hsa-let-7f | TAP1 | 0.09432 | -2 | 0.10899 | -2 | -2 | -2 | activator | Downstream |
| hsa-let-7i | TAP1 | 0.09432 | -2 | 0.10899 | -2 | -2 | -2 | activator | Downstream |
| hsa-miR-222 | TAP1 | 0.09432 | -2 | 0.88949 | -2 | -2 | -2 | activator | Downstream |
| hsa-miR-30a-3p | TAP1 | 0.09432 | -2 | 0.15384 | -2 | -2 | -2 | activator | Downstream |
| hsa-miR-98 | TAP1 | 0.09432 | -2 | 0.34493 | -2 | -2 | -2 | activator | Downstream |
| hsa-miR-148a | TGFA | 0.11277 | 0.81 | -2 | -2 | -2 | -2 | activator | Downstream |
| hsa-miR-23a | TGFA | 0.11277 | 0.7 | -2 | -2 | -2 | -2 | activator | Downstream |
| hsa-miR-200b | ANLN | 0.20708 | 0.84 | 0.58397 | -2 | -2 | -1 | repressor | Downstream |
| hsa-miR-200c | ANLN | 0.20708 | 0.84 | 0.58397 | -2 | -2 | -2 | repressor | Downstream |
| hsa-miR-504 | ANLN | 0.09432 | -2 | 0.31281 | -2 | -2 | -2 | repressor | Downstream |
| hsa-miR-129 | CDC25C | 0.09432 | -2 | 0.79298 | -2 | -2 | -2 | repressor | Downstream |

| | | | | | | | | | |
|---|---|---|---|---|---|---|---|---|---|
| hsa-miR-142-3p | CDC25C | 0.09432 | -2 | 0.99572 | -2 | -2 | -2 | repressor | Downstream |
| hsa-miR-198 | CDC25C | 0.09432 | -2 | 0.3585 | -2 | -2 | -2 | repressor | Downstream |
| hsa-miR-450 | CDC25C | 0.09432 | -2 | 0.16525 | -2 | -2 | -2 | repressor | Downstream |
| hsa-miR-511 | CDC25C | 0.09432 | -2 | 0.99646 | -2 | -2 | -2 | repressor | Downstream |
| hsa-miR-526a | CDC25C | 0.09432 | -2 | 0.69517 | -2 | -2 | -2 | repressor | Downstream |
| hsa-miR-101 | HSPA8 | 0.09432 | -1 | 0.97467 | -2 | -2 | -2 | repressor | Downstream |
| hsa-miR-194 | HSPA8 | 0.09432 | -1 | 0.09274 | -2 | -2 | -2 | repressor | Downstream |
| hsa-miR-33 | HSPA8 | 0.11277 | 0.58 | -1 | -2 | -2 | -2 | repressor | Downstream |
| hsa-miR-519b | HSPA8 | 0.09432 | -2 | 0.86802 | -2 | -2 | -2 | repressor | Downstream |
| hsa-miR-519c | HSPA8 | 0.09432 | -2 | 0.86802 | -2 | -2 | -2 | repressor | Downstream |
| hsa-miR-519d | HSPA8 | 0.09432 | -2 | 0.86802 | -2 | -2 | -2 | repressor | Downstream |
| hsa-miR-142-3p | IER3 | 0.24015 | -2 | 0.71372 | -2 | 0.01825 | -2 | repressor | Downstream |
| hsa-miR-151 | IER3 | 0.09432 | -2 | 0.83899 | -2 | -1 | -2 | repressor | Downstream |
| hsa-miR-502 | NOS3 | 0.09432 | -2 | 0.82979 | -2 | -2 | -2 | repressor | Downstream |
| hsa-miR-192 | ODC1 | 0.09432 | -2 | 0.91022 | -2 | -1 | -2 | repressor | Downstream |
| hsa-miR-26b | ODC1 | 0.09432 | -2 | 0.38156 | -2 | -1 | -2 | repressor | Downstream |
| hsa-let-7a | SCD | 0.14584 | -1 | -1 | -2 | 0.00001 | -1 | repressor | Downstream |
| hsa-let-7b | SCD | 0.14584 | -1 | -1 | -2 | 0.00001 | -1 | repressor | Downstream |
| hsa-let-7f | SCD | 0.14584 | -1 | -1 | -2 | 0.00001 | -1 | repressor | Downstream |
| hsa-let-7i | SCD | 0.14584 | -1 | -1 | -2 | 0.00001 | -1 | repressor | Downstream |
| hsa-miR-124a | SCD | 0.2586 | 0.903 | -1 | -2 | 0.01095 | -2 | repressor | Downstream |
| hsa-miR-200b | SCD | 0.14584 | -1 | -1 | -2 | 0.0073 | -1 | repressor | Downstream |
| hsa-miR-331 | SCD | 0.14584 | -2 | -1 | -2 | 0.00365 | -1 | repressor | Downstream |
| hsa-miR-383 | SCD | 0.14584 | -2 | -1 | -2 | 0.00001 | -2 | repressor | Downstream |
| hsa-miR-429 | SCD | 0.14584 | -2 | -1 | -2 | 0.01095 | -2 | repressor | Downstream |
| hsa-miR-98 | SCD | 0.14584 | -1 | -1 | -2 | 0.00365 | -1 | repressor | Downstream |
| hsa-miR-10a | SLC38A2 | 0.11277 | 0.75 | -1 | -1 | -2 | -2 | repressor | Downstream |
| hsa-miR-10b | SLC38A2 | 0.11277 | 0.75 | -1 | -1 | -2 | -2 | repressor | Downstream |
| hsa-miR-181c | SLC38A2 | 0.11277 | 0.72 | -1 | -1 | -2 | -2 | repressor | Downstream |
| hsa-miR-26b | SLC38A2 | 0.11277 | 0.968 | -1 | -1 | -2 | -2 | repressor | Downstream |
| hsa-miR-30b | SLC38A2 | 0.11277 | 0.75 | -1 | -1 | -2 | -1 | repressor | Downstream |
| hsa-miR-30c | SLC38A2 | 0.11277 | 0.75 | -1 | -1 | -2 | -1 | repressor | Downstream |
| hsa-miR-30d | SLC38A2 | 0.11277 | 0.75 | -1 | -1 | -2 | -2 | repressor | Downstream |
| hsa-miR-32 | SLC38A2 | 0.11277 | 0.75 | -1 | -1 | -2 | -2 | repressor | Downstream |
| hsa-miR-363 | SLC38A2 | 0.41443 | -2 | -1 | 0.41143 | -2 | -2 | repressor | Downstream |
| hsa-miR-92 | SLC38A2 | 0.11277 | 0.75 | -1 | -1 | -2 | -2 | repressor | Downstream |
| hsa-miR-500 | TRPM2 | 0.09432 | -2 | 0.45445 | -2 | -2 | -2 | repressor | Downstream |
| hsa-miR-183 | SCARA3 | 0.09432 | -2 | 0.82208 | -2 | -2 | -2 | unknown | Downstream |
| hsa-miR-182 | THBS2 | 0.11277 | 0.87 | -2 | -2 | -2 | -1 | unknown | Downstream |
| hsa-miR-222 | THBS2 | 0.09432 | -1 | 0.19469 | -2 | -2 | -1 | unknown | Downstream |
| hsa-miR-30b | THBS2 | 0.11277 | 0.936 | -1 | -2 | -2 | -2 | unknown | Downstream |
| hsa-miR-30c | THBS2 | 0.11277 | 0.936 | -1 | -2 | -2 | -1 | unknown | Downstream |
| hsa-miR-30d | THBS2 | 0.11277 | 0.936 | -1 | -2 | -2 | -1 | unknown | Downstream |
| hsa-miR-503 | THBS2 | 0.09432 | -2 | 0.34125 | -2 | -2 | -2 | unknown | Downstream |
| hsa-miR-518a | THBS2 | 0.09432 | -2 | 0.95857 | -2 | -2 | -2 | unknown | Downstream |
| hsa-miR-518b | THBS2 | 0.09432 | -2 | 0.95857 | -2 | -2 | -2 | unknown | Downstream |
| hsa-miR-518c | THBS2 | 0.09432 | -2 | 0.95857 | -2 | -2 | -2 | unknown | Downstream |
| hsa-miR-518d | THBS2 | 0.09432 | -2 | 0.95857 | -2 | -2 | -2 | unknown | Downstream |
| hsa-miR-524 | THBS2 | 0.09432 | -2 | 0.64874 | -2 | -2 | -2 | unknown | Downstream |
| hsa-miR-9 | THBS2 | 0.11277 | 0.88 | -2 | -2 | -2 | -1 | unknown | Downstream |
