# Supplementary material for: Dissecting microregulation of a master regulatory network
Source: BMC Genomics. 2008 Feb 23;9:88. doi: 10.1186/1471-2164-9-88 (PMC2289817; doi:10.1186/1471-2164-9-88)
Supplement: Additional File 4 — Functional enrichment (based on GO and pathways) in target genes of 143 p53-miRs. Results of functional over-representation analysis, performed to objectively identify biological processes potentially affected by p53-miR target genes. [file 1471-2164-9-88-S4.pdf]

Additional File 4: Functional enrichment (based on GO Biological Process) in 12497 target genes of 143 *p53-miRs* using DAVID. The back ground set is genome wide targets of miRNAs.

GO Category Level	GO Term (Biological Process)	No. of *p53-miR* target genes	% of *p53-miR* target genes	p-value	Total No. of miRNA-target genes
GOTERM_BP_5	biopolymer modification	1253	10.26%	4.55E-08	1401
GOTERM_BP_5	phosphate metabolism	648	5.31%	9.47E-06	718
GOTERM_BP_5	regulation of nucleobase, nucleoside, nucleotide and nucleic acid metabolism	1493	12.22%	4.86E-05	1699
GOTERM_BP_5	transcription	1542	12.63%	4.87E-05	1756
GOTERM_BP_5	regulation of progression through cell cycle	367	3.00%	8.41E-04	406
GOTERM_BP_5	protein kinase cascade	228	1.87%	0.001834249	249
GOTERM_BP_5	**vesicle-mediated transport**	297	2.43%	0.00353876	329
GOTERM_BP_5	enzyme linked receptor protein signaling pathway	159	1.30%	0.00395578	172
GOTERM_BP_5	cellular protein metabolism	1966	16.10%	0.006823061	2274
GOTERM_BP_5	regulation of protein kinase activity	114	0.93%	0.006998868	122
GOTERM_BP_5	negative regulation of cellular metabolism	158	1.29%	0.008051397	172
GOTERM_BP_5	protein transport	428	3.50%	0.011965203	483
GOTERM_BP_5	regulation of cell size	123	1.01%	0.023028155	134
GOTERM_BP_5	cell growth	123	1.01%	0.023028155	134
GOTERM_BP_5	regulation of cellular biosynthesis	106	0.87%	0.028106856	115
GOTERM_BP_5	intracellular receptor-mediated signaling pathway	41	0.34%	0.035436058	42
GOTERM_BP_5	negative regulation of progression through cell cycle	123	1.01%	0.03961758	135
GOTERM_BP_5	regulation of protein biosynthesis	101	0.83%	0.042152221	110
GOTERM_BP_5	secretory pathway	151	1.24%	0.059276637	168
GOTERM_BP_5	nuclear transport	88	0.72%	0.06703705	96
GOTERM_BP_5	M phase of mitotic cell cycle	122	1.00%	0.068456762	135
GOTERM_BP_5	nucleocytoplasmic transport	94	0.77%	0.072104423	103
GOTERM_BP_5	apoptosis	411	3.37%	0.072928653	470
GOTERM_BP_5	regulation of programmed cell death	262	2.15%	0.07545553	297
GOTERM_BP_5	regulation of apoptosis	261	2.14%	0.078924672	296
GOTERM_BP_5	negative regulation of programmed cell death	112	0.92%	0.085042646	124
GOTERM_BP_5	exocytosis	56	0.46%	0.089638826	60
GOTERM_BP_5	regulation of protein metabolism	157	1.29%	0.08966786	176
GOTERM_BP_5	polysaccharide metabolism	41	0.34%	0.090523214	43
GOTERM_BP_4	biopolymer metabolism	1970	16.13%	8.18E-06	2246
GOTERM_BP_4	phosphorus metabolism	648	5.31%	1.17E-05	718
GOTERM_BP_4	regulation of cellular metabolism	1593	13.04%	1.65E-05	1810
GOTERM_BP_4	intracellular signaling cascade	839	6.87%	1.57E-04	944
GOTERM_BP_4	negative regulation of cellular physiological process	486	3.98%	3.46E-04	540
GOTERM_BP_4	regulation of cell cycle	368	3.01%	8.72E-04	407
GOTERM_BP_4	**vesicle-mediated transport**	297	2.43%	0.003829613	329
GOTERM_BP_4	mitotic cell cycle	173	1.42%	0.004569708	188
GOTERM_BP_4	cellular macromolecule metabolism	2002	16.39%	0.005410352	2313
GOTERM_BP_4	regulation of kinase activity	114	0.93%	0.007285339	122
GOTERM_BP_4	protein metabolism	2130	17.44%	0.007596265	2465
GOTERM_BP_4	establishment of protein localization	439	3.59%	0.010438917	495
GOTERM_BP_4	protein transport	428	3.50%	0.013034605	483
GOTERM_BP_4	nucleobase, nucleoside, nucleotide and nucleic acid metabolism	2228	18.24%	0.021342394	2587
GOTERM_BP_4	regulation of cell size	123	1.01%	0.023875198	134
GOTERM_BP_4	cellular morphogenesis	208	1.70%	0.03300811	232
GOTERM_BP_4	negative regulation of metabolism	180	1.47%	0.035476541	200
GOTERM_BP_4	regulation of biosynthesis	108	0.88%	0.043823577	118
GOTERM_BP_4	secretory pathway	151	1.24%	0.061353105	168
GOTERM_BP_4	regulation of cell proliferation	226	1.85%	0.072106238	255
GOTERM_BP_4	programmed cell death	412	3.37%	0.07459477	471
GOTERM_BP_4	organelle organization and biogenesis	575	4.71%	0.076533216	661
GOTERM_BP_4	regulation of programmed cell death	262	2.15%	0.078916377	297
GOTERM_BP_4	M phase	153	1.25%	0.080504194	171
GOTERM_BP_4	interphase	56	0.46%	0.091239468	60
GOTERM_BP_4	regulation of protein metabolism	157	1.29%	0.092580271	176
GOTERM_BP_3	regulation of cellular physiological process	2249	18.41%	1.98E-08	2551
GOTERM_BP_3	regulation of metabolism	1637	13.40%	4.42E-05	1865
GOTERM_BP_3	cell cycle	554	4.54%	2.16E-04	617
GOTERM_BP_3	negative regulation of cellular process	540	4.42%	5.04E-04	603
GOTERM_BP_3	negative regulation of physiological process	499	4.09%	0.001156982	558
GOTERM_BP_3	primary metabolism	4671	38.25%	0.001298836	5443
GOTERM_BP_3	cellular metabolism	4744	38.84%	0.005806365	5539
GOTERM_BP_3	regulation of transferase activity	114	0.93%	0.007134428	122
GOTERM_BP_3	cell organization and biogenesis	1080	8.84%	0.007340459	1238
GOTERM_BP_3	cell division	144	1.18%	0.014037848	157
GOTERM_BP_3	protein localization	451	3.69%	0.014186347	510
GOTERM_BP_3	cell proliferation	403	3.30%	0.016550654	455
GOTERM_BP_3	cell growth	123	1.01%	0.023421184	134
GOTERM_BP_3	nervous system development	360	2.95%	0.0279716	407
GOTERM_BP_3	cellular morphogenesis	208	1.70%	0.032219305	232

GOTERM_BP_3	positive regulation of physiological process	358	2.93%	0.068854395	408
GOTERM_BP_3	cell death	424	3.47%	0.090489084	486
GOTERM_BP_3	macromolecule metabolism	2888	23.65%	0.09697427	3377
