# Supplementary material for: Dissecting microregulation of a master regulatory network
Source: BMC Genomics. 2008 Feb 23;9:88. doi: 10.1186/1471-2164-9-88 (PMC2289817; doi:10.1186/1471-2164-9-88)
Supplement: Additional File 5 — Details of Fisher's exact test used to test whether p53-miRs tend to target p53-interactants. Fisher's exact test was used to calculate the probability of p53-miRs regulating the p53 interactants (downloaded from the BioGRID database). [file 1471-2164-9-88-S5.pdf]

Additional File 5: Fisher's exact test was used to calculate the probability of p53-miRs targeting known p53-interactants. Fisher's exact test was performed online at the MATFORSK (http://www.matforsk.no/ola/fisher.htm).

p53-miRs tend to target p53-interactants

We calculated the probability of p53-miRs regulating the p53 interactants. We mined the BioGRID database (Stark et al., 2006) and currently there are 141 known interactants of p53. Surprisingly, 114 of these p53-interactants were predicted to be regulated by p53-miRs and were shown to be statistically significant (p = 1.41e-21).

2x2 Contingency table for validated p53 miRNA target genes & Known p53 interactants

	**Known p53 interactants**			
**Validated p53 miR target genes**		YES	NO	
	YES	114	12383	12497
	NO	27	17476	17503
		141	29859	30000

p-value = 1.41e-21

TABLE = [114, 12383, 27, 17476]
Left : p-value = 1
Right : p-value = **1.4119768198028186e-21**
2-Tail : p-value = 1.6095343985747658e-21

References

Stark, C., Breitkreutz, B.J., Reguly, T., Boucher, L., Breitkreutz, A. and Tyers, M. (2006) BioGRID: a general repository for interaction datasets. *Nucleic Acids Res*, **34**, D535-539.
