# Supplementary material for: Dissecting microregulation of a master regulatory network
Source: BMC Genomics. 2008 Feb 23;9:88. doi: 10.1186/1471-2164-9-88 (PMC2289817; doi:10.1186/1471-2164-9-88)
Supplement: Additional File 7 — Details of the 474 human miRNAs analyzed. Additional details of genomic locations of the 474 human miRNAs analyzed (whether part of a cluster, i.e., 2 or more miRNAs occurring together on the genome; or located in the intergenic or intragenic/intronic regions of known genes). [file 1471-2164-9-88-S7.pdf]

Additional File 7: Out of a total 474 human miRNAs analyzed, 100 microRNAs were part of a cluster (2 or more miRNAs occurring together on the genome). In all, there were 100 miRNAs organized into 37 clusters (see Supplementary Figure 1A also). The cluster definitions were based on those defined by Yu et al [1]. The nearest neighboring gene information (gene name, distance from the nearest miRNA, orientation with respect to the host or neighboring gene) for each of the miRNA was extracted from the public database RefFlat provided by the UCSC Golden Path (http://genome.ucsc.edu). The position type refers to whether the microRNA is present within a gene and the orientation with respect to the host gene ("genic, "same strand" or "diff strand") or in an intergenic space. The comments column indicates the host gene (in case of intragenic or genic miRNAs) or the nearest neighboring genes (in case of intergenic miRNAs). The last column shows miRNA that are clustered together in the genome

1. Yu J, Wang F, Yang GH, Wang FL, Ma YN, Du ZW, Zhang JW: Human microRNA clusters: genomic organization and expression profile in leukemia cell lines. Biochem Biophys Res Commun 2006, 349(1):59-68.

miRNA	position type	comments	miRNA cluster
hsa-mir-324	genic, diff strand	ACADVL	
hsa-mir-486	genic, diff strand	ANK1	
hsa-mir-199b	genic, diff strand	DNM1	
hsa-mir-199a-1	genic, diff strand	DNM2	
hsa-mir-214	genic, diff strand	DNM3	
hsa-mir-199a-2	genic, diff strand	DNM3	
hsa-mir-328	genic, diff strand	ELMO3	
hsa-mir-549	genic, diff strand	KIAA1199	
hsa-mir-610	genic, diff strand	KIF18A	
hsa-mir-133a-1	genic, diff strand	MIB1	
hsa-mir-1-2	genic, diff strand	MIB1	
hsa-mir-662	genic, diff strand	MPFL	
hsa-mir-631	genic, diff strand	NEIL1	
hsa-mir-548d-2	genic, diff strand	PITPNC1	
hsa-mir-599	genic, diff strand	VPS13B	
hsa-mir-632	genic, different genes	C17orf75-ZNF207	
hsa-mir-639	genic, different genes	DNAJB1-GPSN2	
hsa-mir-564	genic, different genes	KIF15-TMEM42	
hsa-mir-657	genic, same strand	AATK	
hsa-mir-338	genic, same strand	AATK	
hsa-mir-95	genic, same strand	ABLIM2	
hsa-mir-641	genic, same strand	AKT2	
hsa-mir-768	genic, same strand	AP1G1	
hsa-mir-765	genic, same strand	ARHGEF11	
hsa-mir-581	genic, same strand	ARL15	
hsa-mir-326	genic, same strand	ARRB1	
hsa-mir-555	genic, same strand	ASH1L	
hsa-mir-488	genic, same strand	ASTN1	
hsa-mir-548d-1	genic, same strand	ATAD2	
hsa-mir-558	genic, same strand	BIRC6	
hsa-mir-609	genic, same strand	C10orf79	
hsa-mir-611	genic, same strand	C11orf10	
hsa-mir-7-3	genic, same strand	C19orf30	
hsa-mir-9-1	genic, same strand	C1orf61	
hsa-mir-1-1	genic, same strand	C20orf166	
hsa-mir-133a-2	genic, same strand	C20orf166	
hsa-mir-99a	genic, same strand	C21orf34	
hsa-let-7c	genic, same strand	C21orf34	
hsa-mir-125b-2	genic, same strand	C21orf34	
hsa-mir-185	genic, same strand	C22orf25	
hsa-mir-567	genic, same strand	C3orf52	
hsa-mir-548b	genic, same strand	C6orf60	
hsa-mir-23b	genic, same strand	C9orf3	
hsa-mir-27b	genic, same strand	C9orf3	
hsa-mir-24-1	genic, same strand	C9orf3	
hsa-mir-32	genic, same strand	C9orf5	
hsa-mir-653	genic, same strand	CALCR	
hsa-mir-489	genic, same strand	CALCR	
hsa-mir-423	genic, same strand	CCDC55	
hsa-mir-628	genic, same strand	CCPG1	
hsa-mir-449	genic, same strand	CDC20B	
hsa-mir-449b	genic, same strand	CDC20B	
hsa-mir-361	genic, same strand	CHM	
hsa-mir-490	genic, same strand	CHRM2	
hsa-mir-455	genic, same strand	COL27A1	
hsa-mir-148b	genic, same strand	COPZ1	
hsa-mir-152	genic, same strand	COPZ2	
hsa-mir-578	genic, same strand	CPE	
hsa-mir-671	genic, same strand	CSGlcA-T	
hsa-mir-26b	genic, same strand	CTDSP1	
hsa-mir-26a-2	genic, same strand	CTDSP2	

hsa-mir-26a-1	genic, same strand	CTDSPL	
hsa-mir-637	genic, same strand	DAPK3	
hsa-mir-616	genic, same strand	DDIT3	
hsa-mir-601	genic, same strand	DENND1A	
hsa-mir-562	genic, same strand	DIS3L2	
hsa-mir-638	genic, same strand	DNM2	
hsa-mir-126	genic, same strand	EGFL7	
hsa-mir-590	genic, same strand	EIF4H	
hsa-mir-330	genic, same strand	EML2	
hsa-mir-342	genic, same strand	EVL	
hsa-mir-574	genic, same strand	FAM114A1	
hsa-mir-454	genic, same strand	FAM33A	
hsa-mir-301	genic, same strand	FAM33A	
hsa-mir-589	genic, same strand	FBXL18	
hsa-mir-504	genic, same strand	FGF13	
hsa-mir-198	genic, same strand	FSTL1	
hsa-mir-625	genic, same strand	FUT8	
hsa-mir-642	genic, same strand	GIPR	
hsa-mir-149	genic, same strand	GPC1	
hsa-mir-346	genic, same strand	GRID1	
hsa-mir-592	genic, same strand	GRM8	
hsa-mir-561	genic, same strand	GULP1	
hsa-mir-7-1	genic, same strand	HNRPK	
hsa-mir-615	genic, same strand	HOXC5	
hsa-mir-448	genic, same strand	HTR2C	
hsa-mir-483	genic, same strand	IGF2	
hsa-mir-644	genic, same strand	ITCH	
hsa-mir-603	genic, same strand	KIAA1217	
hsa-mir-491	genic, same strand	KIAA1797	
hsa-mir-617	genic, same strand	LIN7A	
hsa-mir-618	genic, same strand	LIN7A	
hsa-mir-580	genic, same strand	LMBRD2	
hsa-mir-28	genic, same strand	LPP	
hsa-mir-25	genic, same strand	MCM7	
hsa-mir-93	genic, same strand	MCM7	
hsa-mir-106b	genic, same strand	MCM7	
hsa-mir-551a	genic, same strand	MEGF6	
hsa-mir-335	genic, same strand	MEST	
hsa-mir-626	genic, same strand	MGA	
hsa-mir-339	genic, same strand	MGC11257	
hsa-mir-22	genic, same strand	MGC14376	
hsa-mir-511-1	genic, same strand	MRC1	
hsa-mir-511-2	genic, same strand	MRC1L1	
hsa-mir-208	genic, same strand	MYH6	
hsa-mir-499	genic, same strand	MYH7B	
hsa-mir-30e	genic, same strand	NFYC	
hsa-mir-30c-1	genic, same strand	NFYC	
hsa-mir-556	genic, same strand	NOS1AP	
hsa-mir-107	genic, same strand	PANK1	
hsa-mir-103-2	genic, same strand	PANK2	
hsa-mir-103-1	genic, same strand	PANK3	
hsa-mir-139	genic, same strand	PDE2A	
hsa-mir-661	genic, same strand	PLEC1	
hsa-mir-378	genic, same strand	PPARGC1B	
hsa-mir-634	genic, same strand	PRKCA	
hsa-mir-605	genic, same strand	PRKG1	
hsa-mir-151	genic, same strand	PTK2	
hsa-mir-153-1	genic, same strand	PTPRN	
hsa-mir-153-2	genic, same strand	PTPRN2	
hsa-mir-595	genic, same strand	PTPRN2	
hsa-mir-128a	genic, same strand	R3HDM1	
hsa-mir-548c	genic, same strand	RASSF3	
hsa-mir-101-2	genic, same strand	RCL1	
hsa-mir-340	genic, same strand	RNF130	
hsa-mir-553	genic, same strand	RTCD1	
hsa-mir-575	genic, same strand	SCD5	
hsa-mir-576	genic, same strand	SEC24B	
hsa-mir-566	genic, same strand	SEMA3F	
hsa-mir-608	genic, same strand	SEMA4G	
hsa-mir-766	genic, same strand	SEPT6	
hsa-mir-636	genic, same strand	SFRS2	
hsa-mir-383	genic, same strand	SGCZ	
hsa-mir-584	genic, same strand	SH3TC2	

hsa-mir-591	genic, same strand	SLC25A13	
hsa-mir-218-1	genic, same strand	SLIT2	
hsa-mir-218-2	genic, same strand	SLIT3	
hsa-mir-585	genic, same strand	SLIT3	
hsa-mir-15b	genic, same strand	SMC4	
hsa-mir-16-2	genic, same strand	SMC4	
hsa-mir-593	genic, same strand	SND1	
hsa-mir-33b	genic, same strand	SREBF1	
hsa-mir-33	genic, same strand	SREBF2	
hsa-mir-619	genic, same strand	SSH1	
hsa-mir-658	genic, same strand	starts in ANKRD54, ends in intergenic	
hsa-mir-624	genic, same strand	STRN3	
hsa-mir-586	genic, same strand	SUPT3H	
hsa-mir-604	genic, same strand	SVIL	
hsa-mir-559	genic, same strand	TACSTD1	
hsa-mir-620	genic, same strand	THRAP2	
hsa-mir-629	genic, same strand	TLE3	
hsa-mir-190	genic, same strand	TLN2	
hsa-let-7g	genic, same strand	TMEM113	
hsa-mir-652	genic, same strand	TMEM164	
hsa-mir-569	genic, same strand	TNIK	
hsa-mir-597	genic, same strand	TNKS	
hsa-mir-211	genic, same strand	TRPM1	
hsa-mir-204	genic, same strand	TRPM3	
hsa-mir-554	genic, same strand	TUFT1	
hsa-mir-647	genic, same strand	UCKL1	
hsa-mir-577	genic, same strand	UGT8	
hsa-mir-627	genic, same strand	VPS39	
hsa-mir-635	genic, same strand	WIPI1	
hsa-mir-140	genic, same strand	WWP2	
hsa-mir-598	genic, same strand	XKR6	
hsa-mir-579	genic, same strand	ZFR	
hsa-mir-571	genic, same strand	ZNF141	
hsa-mir-643	genic, same strand	ZNF766	
hsa-mir-550-1	genic, same strand	ZNRF2	
hsa-mir-186	genic, same strand	ZRANB2	
hsa-mir-219-1	intergenic, < 10kb	1004 bp downstream of HSD17B8, 564 bp upstream of RING1	
hsa-mir-607	intergenic, < 10kb	108146 bp downstream of PIK3AP1, 4215 bp upstream of LCOR	
hsa-mir-150	intergenic, < 10kb	1097 bp downstream of RPS11, 12366 bp upstream of FCGRT	
hsa-mir-645	intergenic, < 10kb	1238 bp downstream of PTPN1, 229 bp upstream of C20orf175	
hsa-mir-320	intergenic, < 10kb	12870 bp downstream of PHYHIP, 62 bp upstream of POLR3D	
hsa-mir-345	intergenic, < 10kb	1335 bp downstream of SLC25A29, 15385 bp upstream of C14orf68	
hsa-mir-138-2	intergenic, < 10kb	13753 bp downstream of NUP93, 6628 bp upstream of SLC12A3	
hsa-mir-484	intergenic, < 10kb	141 bp downstream of KIAA0430, 6874 bp upstream of NDE1	
hsa-mir-10a	intergenic, < 10kb	1456 bp downstream of HOXB4, 11309 bp upstream of HOXB5	
hsa-mir-196a-2	intergenic, < 10kb	1461 bp downstream of HOXC10, 8245 bp upstream of HOXC9	
hsa-mir-594	intergenic, < 10kb	150910 bp downstream of TTC26, 529 bp upstream of HSPC268	
hsa-mir-649	intergenic, < 10kb	1617 bp downstream of SLC7A4, 11708 bp upstream of LOC400891	
hsa-mir-21	intergenic, < 10kb	173 bp downstream of TMEM49, 18152 bp upstream of TUBD1	
hsa-mir-548a-3	intergenic, < 10kb	17319 bp downstream of DPYS, 4773 bp upstream of LRP12	
hsa-mir-10b	intergenic, < 10kb	18304 bp downstream of HOXD8, 972 bp upstream of HOXD4	
hsa-mir-219-2	intergenic, < 10kb	1884 bp downstream of URM1, 28123 bp upstream of CEECAM1	
hsa-mir-614	intergenic, < 10kb	2164 bp downstream of GPRC5A, 24858 bp upstream of GPRC5D	
hsa-mir-92b	intergenic, < 10kb	2267 bp downstream of MUC1, 317 bp upstream of THBS3	
hsa-let-7i	intergenic, < 10kb	251 bp downstream of C12orf61, 542666 bp upstream of AVPR1A	
hsa-mir-130a	intergenic, < 10kb	26344 bp downstream of SERPING1, 3801 bp upstream of YPEL4	
hsa-mir-223	intergenic, < 10kb	276919 bp downstream of MSN, 2758 bp upstream of VSIG4	
hsa-mir-596	intergenic, < 10kb	30661 bp downstream of C8orf61, 6675 bp upstream of ARHGEF10	
hsa-mir-142	intergenic, < 10kb	3114 bp downstream of BZRAP1, 13859 bp upstream of SUPT4H1	
hsa-mir-451	intergenic, < 10kb	314 bp downstream of ERAL1, 17899 bp upstream of FLOT2	
hsa-mir-769	intergenic, < 10kb	315 bp downstream of LOC729440, 133 bp upstream of PGLYRP1	
hsa-mir-659	intergenic, < 10kb	3381 bp downstream of ANKRD54, 1588 bp upstream of EIF3S6IP	
hsa-mir-630	intergenic, < 10kb	3734 bp downstream of ARIH1, 67383 bp upstream of GOLGA	
hsa-mir-146b	intergenic, < 10kb	3845 bp downstream of CUEDC2, 13252 bp upstream of C10orf95	
hsa-mir-497	intergenic, < 10kb	391 bp downstream of C17orf49, 5027 bp upstream of BCL6B	
hsa-mir-196b	intergenic, < 10kb	3949 bp downstream of HOXA9, 1028 bp upstream of HOXA10	
hsa-mir-210	intergenic, < 10kb	4070 bp downstream of RASSF7, 8287 bp upstream of KIAA1542	
hsa-mir-197	intergenic, < 10kb	4539 bp downstream of GNAI3, 4300 bp upstream of GNAT2	
hsa-mir-144	intergenic, < 10kb	478 bp downstream of ERAL1, 17721 bp upstream of FLOT2	
hsa-mir-203	intergenic, < 10kb	4816 bp downstream of LOC374569, 463496 bp upstream of LOC400258	
hsa-mir-801	intergenic, < 10kb	5525 bp downstream of TAF12, 20037 bp upstream of GMEB1	
hsa-mir-202	intergenic, < 10kb	5582 bp downstream of VENTX, 14796 bp upstream of ADAM8	
hsa-mir-196a-1	intergenic, < 10kb	6016 bp downstream of HOXB9, 89170 bp upstream of PRAC	

hsa-mir-573	intergenic, < 10kb	630114 bp downstream of PPARGC1A, 7176 bp upstream of DHX15	
hsa-mir-7-2	intergenic, < 10kb	65171 bp downstream of DET1, 9423 bp upstream of ISG20L1	
hsa-mir-621	intergenic, < 10kb	655 bp downstream of SLC25A15, 121167 bp upstream of ELF1	
hsa-mir-568	intergenic, < 10kb	6973 bp downstream of VSIG9, 22106 bp upstream of ZBTB20	
hsa-mir-331	intergenic, < 10kb	7249 bp downstream of VEZT, 165532 bp upstream of METAP2	
hsa-mir-135a-1	intergenic, < 10kb	742 bp downstream of GLYCTK, 22195 bp upstream of DNAH1	
hsa-mir-565	intergenic, < 10kb	7709 bp downstream of LIMD1, 382 bp upstream of SACM1L	
hsa-mir-375	intergenic, < 10kb	8239 bp downstream of CRYBA2, 1145 bp upstream of CCDC108	
hsa-mir-130b	intergenic, < 10kb	9005 bp downstream of SDF2L1, 12598 bp upstream of PPIL2	
hsa-mir-195	intergenic, < 10kb	95 bp downstream of C17orf49, 5348 bp upstream of BCL6B	
hsa-mir-30a	intergenic, > 10kb	101282 bp downstream of OGFRL1, 483325 bp upstream of RIMS1	
hsa-mir-296	intergenic, > 10kb	101758 bp downstream of NPEPL1, 22045 bp upstream of GNAS	
hsa-mir-518a-2	intergenic, > 10kb	102323 bp downstream of DPRX, 54183 bp upstream of NLRP12	
hsa-mir-548a-1	intergenic, > 10kb	103166 bp downstream of IBRDC2, 1265505 bp upstream of ID4	
hsa-mir-30b	intergenic, > 10kb	103968 bp downstream of ZFAT1, 656865 bp upstream of KHDRBS3	
hsa-mir-517c	intergenic, > 10kb	104303 bp downstream of DPRX, 52195 bp upstream of NLRP12	
hsa-mir-520h	intergenic, > 10kb	105502 bp downstream of DPRX, 51003 bp upstream of NLRP12	
hsa-mir-30d	intergenic, > 10kb	108324 bp downstream of ZFAT1, 652527 bp upstream of KHDRBS3	
hsa-mir-521-1	intergenic, > 10kb	111626 bp downstream of DPRX, 44880 bp upstream of NLRP12	
hsa-mir-522	intergenic, > 10kb	114201 bp downstream of DPRX, 42305 bp upstream of NLRP12	
hsa-mir-519a-1	intergenic, > 10kb	115387 bp downstream of DPRX, 41121 bp upstream of NLRP12	
hsa-mir-587	intergenic, > 10kb	116589 bp downstream of QRSL1, 117321 bp upstream of C6orf203	
hsa-mir-124a-2	intergenic, > 10kb	1166360 bp downstream of YTHDF3, 200999 bp upstream of BHLHB5	
hsa-mir-527	intergenic, > 10kb	117008 bp downstream of DPRX, 39500 bp upstream of NLRP12	
hsa-mir-583	intergenic, > 10kb	117066 bp downstream of ELL2, 311202 bp upstream of PCSK1	
hsa-mir-582	intergenic, > 10kb	117156 bp downstream of PDE4D, 893210 bp upstream of DEPDC1B	
hsa-mir-770	intergenic, > 10kb	117269 bp downstream of DLK1, 152820 bp upstream of FLJ41170	
hsa-mir-205	intergenic, > 10kb	1187812 bp downstream of PLXNA2, 151457 bp upstream of CAMK1G	
hsa-mir-570	intergenic, > 10kb	119173 bp downstream of APOD, 21971 bp upstream of MUC20	
hsa-mir-184	intergenic, > 10kb	119289 bp downstream of RASGRF1, 101277 bp upstream of TMED3	
hsa-mir-516-1	intergenic, > 10kb	119731 bp downstream of DPRX, 36772 bp upstream of NLRP12	
hsa-mir-124a-1	intergenic, > 10kb	121042 bp downstream of TNKS, 150843 bp upstream of MSRA	
hsa-mir-137	intergenic, > 10kb	125010 bp downstream of DPYD, 615485 bp upstream of SNX7	
hsa-mir-182	intergenic, > 10kb	13302 bp downstream of NRF1, 62662 bp upstream of UBE2H	
hsa-mir-493	intergenic, > 10kb	133939 bp downstream of DLK1, 136159 bp upstream of FLJ41170	
hsa-mir-633	intergenic, > 10kb	135880 bp downstream of RNF190, 487992 bp upstream of CYB561	
hsa-mir-588	intergenic, > 10kb	136028 bp downstream of C6orf173, 634188 bp upstream of RSPO3	
hsa-mir-513-1	intergenic, > 10kb	1383612 bp downstream of CXorf1, 698399 bp upstream of FMR1	
hsa-mir-337	intergenic, > 10kb	139372 bp downstream of DLK1, 130722 bp upstream of FLJ41170	
hsa-mir-513-2	intergenic, > 10kb	1395975 bp downstream of CXorf1, 686038 bp upstream of FMR1	
hsa-mir-508	intergenic, > 10kb	1407062 bp downstream of CXorf1, 674963 bp upstream of FMR1	
hsa-mir-563	intergenic, > 10kb	14224 bp downstream of ANKRD28, 300799 bp upstream of GALNTL2	
hsa-mir-509	intergenic, > 10kb	1430681 bp downstream of CXorf1, 651365 bp upstream of FMR1	
hsa-mir-510	intergenic, > 10kb	1442484 bp downstream of CXorf1, 639582 bp upstream of FMR1	
hsa-mir-431	intergenic, > 10kb	145886 bp downstream of DLK1, 124187 bp upstream of FLJ41170	
hsa-mir-433	intergenic, > 10kb	146765 bp downstream of DLK1, 123329 bp upstream of FLJ41170	
hsa-mir-550-2	intergenic, > 10kb	148813 bp downstream of KIAA0241, 135096 bp upstream of KBTBD2	
hsa-mir-606	intergenic, > 10kb	150790 bp downstream of ZNF503, 230207 bp upstream of C10orf11	
hsa-mir-532	intergenic, > 10kb	169186 bp downstream of PAGE4, 64370 bp upstream of CLCN5	
hsa-mir-188	intergenic, > 10kb	169540 bp downstream of PAGE4, 64020 bp upstream of CLCN5	
hsa-mir-124a-3	intergenic, > 10kb	171509 bp downstream of BHLHB4, 16845 bp upstream of YTHDF1	
hsa-mir-650	intergenic, > 10kb	174901 bp downstream of GGTL4, 236228 bp upstream of RTDR1	
hsa-mir-370	intergenic, > 10kb	176018 bp downstream of DLK1, 94094 bp upstream of FLJ41170	
hsa-mir-129-1	intergenic, > 10kb	176922 bp downstream of LRRC4, 33334 bp upstream of LEP	
hsa-mir-660	intergenic, > 10kb	179280 bp downstream of PAGE4, 54269 bp upstream of CLCN5	
hsa-mir-502	intergenic, > 10kb	180637 bp downstream of PAGE4, 52923 bp upstream of CLCN5	
hsa-mir-492	intergenic, > 10kb	183849 bp downstream of TMCC3, 136821 bp upstream of NDUFA12	
hsa-mir-758	intergenic, > 10kb	18987 bp downstream of FLJ41170, 535243 bp upstream of DIO3	
hsa-mir-187	intergenic, > 10kb	192986 bp downstream of GALNT1, 67698 bp upstream of C18orf21	
hsa-mir-329-1	intergenic, > 10kb	19752 bp downstream of FLJ41170, 534486 bp upstream of DIO3	
hsa-mir-651	intergenic, > 10kb	199562 bp downstream of PNPLA4, 42883 bp upstream of VCX2	
hsa-mir-548a-2	intergenic, > 10kb	19988 bp downstream of MYB, 44730 bp upstream of AHI1	
hsa-mir-329-2	intergenic, > 10kb	20067 bp downstream of FLJ41170, 534167 bp upstream of DIO3	
hsa-mir-648	intergenic, > 10kb	206375 bp downstream of BID, 96977 bp upstream of PEX26	
hsa-mir-193a	intergenic, > 10kb	21775 bp downstream of RAB11FIP4, 291796 bp upstream of C17orf79	
hsa-mir-34a	intergenic, > 10kb	22497 bp downstream of GPR157, 83026 bp upstream of H6PD	
hsa-mir-494	intergenic, > 10kb	22601 bp downstream of FLJ41170, 531636 bp upstream of DIO3	
hsa-mir-646	intergenic, > 10kb	235523 bp downstream of C20orf197, 943933 bp upstream of CDH4	
hsa-mir-622	intergenic, > 10kb	2551567 bp downstream of SLITRK5, 1167355 bp upstream of GPC5	
hsa-mir-135b	intergenic, > 10kb	26248 bp downstream of LEMD1, 56200 bp upstream of PCTK3	
hsa-mir-495	intergenic, > 10kb	26722 bp downstream of FLJ41170, 527514 bp upstream of DIO3	
hsa-mir-16-1	intergenic, > 10kb	28051 bp downstream of KCNRG, 662839 bp upstream of DLEU7	
hsa-mir-15a	intergenic, > 10kb	28197 bp downstream of KCNRG, 662699 bp upstream of DLEU7	
hsa-mir-512-1	intergenic, > 10kb	29669 bp downstream of DPRX, 126840 bp upstream of NLRP12	

hsa-mir-31	intergenic, > 10kb	29801 bp downstream of IFNE1, 290450 bp upstream of MTAP	
hsa-let-7a-2	intergenic, > 10kb	30306 bp downstream of BRCC2, 509128 bp upstream of STS-1	
hsa-mir-612	intergenic, > 10kb	30935 bp downstream of FKSG44, 80519 bp upstream of SCYL1	
hsa-mir-512-2	intergenic, > 10kb	32147 bp downstream of DPRX, 124348 bp upstream of NLRP12	
hsa-mir-9-3	intergenic, > 10kb	33221 bp downstream of POLG, 103304 bp upstream of RHCG	
hsa-mir-100	intergenic, > 10kb	36013 bp downstream of BRCC2, 503413 bp upstream of STS-1	
hsa-mir-17	intergenic, > 10kb	3670990 bp downstream of SLITRK5, 47944 bp upstream of GPC5	
hsa-mir-18a	intergenic, > 10kb	3671136 bp downstream of SLITRK5, 47811 bp upstream of GPC5	
hsa-mir-19a	intergenic, > 10kb	3671276 bp downstream of SLITRK5, 47660 bp upstream of GPC5	
hsa-mir-20a	intergenic, > 10kb	3671450 bp downstream of SLITRK5, 47497 bp upstream of GPC5	
hsa-mir-19b-1	intergenic, > 10kb	3671577 bp downstream of SLITRK5, 47354 bp upstream of GPC5	
hsa-mir-92-1	intergenic, > 10kb	3671699 bp downstream of SLITRK5, 47241 bp upstream of GPC5	
hsa-mir-422a	intergenic, > 10kb	36981 bp downstream of HERC1, 36016 bp upstream of DAPK2	
hsa-mir-498	intergenic, > 10kb	37187 bp downstream of DPRX, 119282 bp upstream of NLRP12	
hsa-mir-365-2	intergenic, > 10kb	37190 bp downstream of RAB11FIP4, 276358 bp upstream of C17orf79	
hsa-mir-193b	intergenic, > 10kb	37194 bp downstream of MKL2, 131652 bp upstream of PARN	
hsa-mir-520e	intergenic, > 10kb	38701 bp downstream of DPRX, 117805 bp upstream of NLRP12	
hsa-mir-381	intergenic, > 10kb	38887 bp downstream of FLJ41170, 515356 bp upstream of DIO3	
hsa-mir-487b	intergenic, > 10kb	39422 bp downstream of FLJ41170, 514812 bp upstream of DIO3	
hsa-mir-138-1	intergenic, > 10kb	395412 bp downstream of ABHD5, 223830 bp upstream of C3orf23	
hsa-mir-9-2	intergenic, > 10kb	398005 bp downstream of TMEM161B, 53408 bp upstream of MEF2C	
hsa-mir-539	intergenic, > 10kb	40288 bp downstream of FLJ41170, 513952 bp upstream of DIO3	
hsa-mir-155	intergenic, > 10kb	4035077 bp downstream of NCAM2, 11613 bp upstream of MRPL39	
hsa-mir-542	intergenic, > 10kb	40674 bp downstream of HPRT1, 24405 bp upstream of PLAC1	
hsa-mir-544	intergenic, > 10kb	41625 bp downstream of FLJ41170, 512602 bp upstream of DIO3	
hsa-mir-515-1	intergenic, > 10kb	41993 bp downstream of DPRX, 114517 bp upstream of NLRP12	
hsa-mir-613	intergenic, > 10kb	42280 bp downstream of CDKN1B, 20937 bp upstream of APOLD1	
hsa-mir-365-1	intergenic, > 10kb	42512 bp downstream of MKL2, 126330 bp upstream of PARN	
hsa-mir-655	intergenic, > 10kb	42517 bp downstream of FLJ41170, 511704 bp upstream of DIO3	
hsa-mir-519e	intergenic, > 10kb	42930 bp downstream of DPRX, 113579 bp upstream of NLRP12	
hsa-mir-552	intergenic, > 10kb	450470 bp downstream of C1orf94, 85425 bp upstream of GJB5	
hsa-mir-520f	intergenic, > 10kb	45149 bp downstream of DPRX, 111357 bp upstream of NLRP12	
hsa-mir-487a	intergenic, > 10kb	45413 bp downstream of FLJ41170, 508825 bp upstream of DIO3	
hsa-mir-551b	intergenic, > 10kb	455972 bp downstream of GOLPH4, 532812 bp upstream of EVI1	
hsa-mir-560	intergenic, > 10kb	456266 bp downstream of MGC50273, 158551 bp upstream of GPR39	
hsa-mir-125b-1	intergenic, > 10kb	470077 bp downstream of SORL1, 15509 bp upstream of BRCC2	
hsa-mir-515-2	intergenic, > 10kb	47999 bp downstream of DPRX, 108511 bp upstream of NLRP12	
hsa-mir-668	intergenic, > 10kb	48225 bp downstream of FLJ41170, 506027 bp upstream of DIO3	
hsa-mir-485	intergenic, > 10kb	48386 bp downstream of FLJ41170, 505859 bp upstream of DIO3	
hsa-mir-623	intergenic, > 10kb	48690 bp downstream of EBI2, 145245 bp upstream of TM9SF2	
hsa-mir-384	intergenic, > 10kb	487943 bp downstream of MAGEE1, 540123 bp upstream of FGF16	
hsa-mir-453	intergenic, > 10kb	49157 bp downstream of FLJ41170, 505081 bp upstream of DIO3	
hsa-mir-519c	intergenic, > 10kb	49459 bp downstream of DPRX, 107047 bp upstream of NLRP12	
hsa-mir-122a	intergenic, > 10kb	52916 bp downstream of NEDD4L, 30091 bp upstream of ALPK2	
hsa-mir-421	intergenic, > 10kb	533967 bp downstream of CHIC1, 85728 bp upstream of ZCCHC13	
hsa-mir-520a	intergenic, > 10kb	53871 bp downstream of DPRX, 102637 bp upstream of NLRP12	
hsa-mir-146a	intergenic, > 10kb	56613 bp downstream of PTTG1, 802978 bp upstream of GABRB2	
hsa-mir-206	intergenic, > 10kb	56723 bp downstream of PKHD1, 41952 bp upstream of IL17A	
hsa-mir-526b	intergenic, > 10kb	57383 bp downstream of DPRX, 99127 bp upstream of NLRP12	
hsa-mir-325	intergenic, > 10kb	574071 bp downstream of MAGEE1, 453985 bp upstream of FGF16	
hsa-mir-519b	intergenic, > 10kb	58203 bp downstream of DPRX, 98309 bp upstream of NLRP12	
hsa-mir-409	intergenic, > 10kb	58267 bp downstream of FLJ41170, 495972 bp upstream of DIO3	
hsa-mir-412	intergenic, > 10kb	58414 bp downstream of FLJ41170, 495813 bp upstream of DIO3	
hsa-mir-369	intergenic, > 10kb	58565 bp downstream of FLJ41170, 495683 bp upstream of DIO3	
hsa-mir-128b	intergenic, > 10kb	58590 bp downstream of ARPP-21, 636045 bp upstream of STAC	
hsa-mir-410	intergenic, > 10kb	58879 bp downstream of FLJ41170, 495359 bp upstream of DIO3	
hsa-mir-217	intergenic, > 10kb	59169 bp downstream of EFEMP1, 201046 bp upstream of CCDC85A	
hsa-mir-656	intergenic, > 10kb	59691 bp downstream of FLJ41170, 494549 bp upstream of DIO3	
hsa-mir-545	intergenic, > 10kb	602694 bp downstream of CHIC1, 16980 bp upstream of ZCCHC13	
hsa-mir-374	intergenic, > 10kb	602876 bp downstream of CHIC1, 16832 bp upstream of ZCCHC13	
hsa-mir-525	intergenic, > 10kb	60523 bp downstream of DPRX, 95985 bp upstream of NLRP12	
hsa-mir-557	intergenic, > 10kb	61099 bp downstream of TBX19, 165144 bp upstream of XCL2	
hsa-mir-135a-2	intergenic, > 10kb	611550 bp downstream of NEDD1, 951719 bp upstream of TMPO	
hsa-mir-133b	intergenic, > 10kb	61297 bp downstream of PKHD1, 37345 bp upstream of IL17A	
hsa-mir-523	intergenic, > 10kb	61375 bp downstream of DPRX, 95131 bp upstream of NLRP12	
hsa-mir-518f	intergenic, > 10kb	63005 bp downstream of DPRX, 93501 bp upstream of NLRP12	
hsa-mir-520b	intergenic, > 10kb	64217 bp downstream of DPRX, 92315 bp upstream of NLRP12	
hsa-mir-216	intergenic, > 10kb	65152 bp downstream of EFEMP1, 195063 bp upstream of CCDC85A	
hsa-mir-518b	intergenic, > 10kb	65727 bp downstream of DPRX, 90783 bp upstream of NLRP12	
hsa-mir-802	intergenic, > 10kb	671417 bp downstream of RUNX1, 313733 bp upstream of SETD4	
hsa-mir-572	intergenic, > 10kb	683961 bp downstream of MIST, 29443 bp upstream of HS3ST1	
hsa-let-7d	intergenic, > 10kb	68979 bp downstream of PTPDC1, 80375 bp upstream of ZNF169	
hsa-mir-220	intergenic, > 10kb	71179 bp downstream of GRIA3, 38356 bp upstream of THOC2	

hsa-mir-148a	intergenic, > 10kb	721433 bp downstream of NPVF, 202253 bp upstream of NFE2L3	
hsa-mir-602	intergenic, > 10kb	73648 bp downstream of FLJ40292, 39272 bp upstream of CACNA1B	
hsa-mir-30c-2	intergenic, > 10kb	74691 bp downstream of OGFRL1, 509915 bp upstream of RIMS1	
hsa-mir-600	intergenic, > 10kb	75930 bp downstream of GPR21, 13397 bp upstream of STRBP	
hsa-mir-640	intergenic, > 10kb	76308 bp downstream of KIAA0892, 23429 bp upstream of GATAD2A	
hsa-mir-521-2	intergenic, > 10kb	79584 bp downstream of DPRX, 76922 bp upstream of NLRP12	
hsa-mir-520d	intergenic, > 10kb	83086 bp downstream of DPRX, 73420 bp upstream of NLRP12	
hsa-mir-517b	intergenic, > 10kb	84066 bp downstream of DPRX, 72460 bp upstream of NLRP12	
hsa-mir-520g	intergenic, > 10kb	85156 bp downstream of DPRX, 71347 bp upstream of NLRP12	
hsa-mir-129-2	intergenic, > 10kb	87448 bp downstream of TTC17, 99271 bp upstream of HSD17B12	
hsa-mir-147	intergenic, > 10kb	875517 bp downstream of DBC1, 143819 bp upstream of CDK5RAP2	
hsa-mir-516-3	intergenic, > 10kb	88432 bp downstream of DPRX, 68076 bp upstream of NLRP12	
hsa-mir-526a-2	intergenic, > 10kb	89912 bp downstream of DPRX, 66616 bp upstream of NLRP12	
hsa-mir-505	intergenic, > 10kb	91859 bp downstream of ATP11C, 31494 bp upstream of LOC347487	
hsa-mir-101-1	intergenic, > 10kb	91929 bp downstream of JAK1, 89040 bp upstream of AK3L1	
hsa-mir-518e	intergenic, > 10kb	92828 bp downstream of DPRX, 63677 bp upstream of NLRP12	
hsa-mir-518a-1	intergenic, > 10kb	93996 bp downstream of DPRX, 62512 bp upstream of NLRP12	
hsa-mir-663	intergenic, > 10kb	94123 bp downstream of C20orf191, 3172213 bp upstream of DEFB115	
hsa-mir-518d	intergenic, > 10kb	97867 bp downstream of DPRX, 58639 bp upstream of NLRP12	
hsa-mir-516-4	intergenic, > 10kb	99835 bp downstream of DPRX, 56668 bp upstream of NLRP12	
hsa-mir-191	genic, diff strand	C3orf60 (DALRD3, same strand)	clus-191
hsa-mir-194-1	genic, diff strand	IARS2	clus-215
hsa-mir-215	genic, diff strand	IARS2	clus-215
hsa-mir-302b	genic, diff strand	LARP7	clus-302
hsa-mir-302c	genic, diff strand	LARP7	clus-302
hsa-mir-302a	genic, diff strand	LARP7	clus-302
hsa-mir-302d	genic, diff strand	LARP7	clus-302
hsa-mir-367	genic, diff strand	LARP7	clus-302
hsa-mir-181a-2	genic, diff strand	NR6A1	clus-181a-2
hsa-mir-181b-2	genic, diff strand	NR6A1	clus-181a-2
hsa-mir-425	genic, same strand	DALRD3	clus-191
hsa-mir-767	genic, same strand	GABRA3	clus-105
hsa-mir-105-2	genic, same strand	GABRA3	clus-105
hsa-mir-105-1	genic, same strand	GABRA3	clus-105
hsa-mir-452	genic, same strand	GABRE	clus-452
hsa-mir-224	genic, same strand	GABRE	clus-452
hsa-let-7f-2	genic, same strand	HUWE1	clus-let-7f-2
hsa-mir-98	genic, same strand	HUWE1	clus-let-7f-2
hsa-mir-34c	intergenic, < 10kb	1099 bp downstream of BTG4, 1269 bp upstream of FLJ46266	clus-34b
hsa-mir-192	intergenic, < 10kb	12417 bp downstream of EHD1, 3300 bp upstream of KIAA0404	clus-192
hsa-mir-194-2	intergenic, < 10kb	12635 bp downstream of EHD1, 3107 bp upstream of KIAA0404	clus-192
hsa-mir-371	intergenic, < 10kb	150665 bp downstream of DPRX, 5861 bp upstream of NLRP12	clus-371
hsa-mir-372	intergenic, < 10kb	150880 bp downstream of DPRX, 5646 bp upstream of NLRP12	clus-371
hsa-mir-373	intergenic, < 10kb	151695 bp downstream of DPRX, 4829 bp upstream of NLRP12	clus-371
hsa-mir-200c	intergenic, < 10kb	2382 bp downstream of PTPN6, 1587 bp upstream of PHB2	clus-200c
hsa-mir-141	intergenic, < 10kb	2780 bp downstream of PTPN6, 1162 bp upstream of PHB2	clus-200c
hsa-mir-24-2	intergenic, < 10kb	4056 bp downstream of ZSWIM4, 45994 bp upstream of C19orf57	clus-23a
hsa-mir-27a	intergenic, < 10kb	4209 bp downstream of ZSWIM4, 45836 bp upstream of C19orf57	clus-23a
hsa-mir-181c	intergenic, < 10kb	42468 bp downstream of ZSWIM4, 7545 bp upstream of C19orf57	clus-181c
hsa-mir-181d	intergenic, < 10kb	42644 bp downstream of ZSWIM4, 7342 bp upstream of C19orf57	clus-181c
hsa-mir-23a	intergenic, < 10kb	4356 bp downstream of ZSWIM4, 45694 bp upstream of C19orf57	clus-23a
hsa-mir-34b	intergenic, < 10kb	598 bp downstream of BTG4, 1763 bp upstream of FLJ46266	clus-34b
hsa-mir-29c	intergenic, < 10kb	6339 bp downstream of CD46, 84599 bp upstream of CD34	clus-29c
hsa-mir-132	intergenic, < 10kb	6477 bp downstream of OVCA2, 5090 bp upstream of HIC1	clus-212
hsa-mir-212	intergenic, < 10kb	6840 bp downstream of OVCA2, 4718 bp upstream of HIC1	clus-212
hsa-mir-29b-2	intergenic, < 10kb	6930 bp downstream of CD46, 84015 bp upstream of CD34	clus-29c
hsa-mir-181b-1	intergenic, > 10kb	101456 bp downstream of PTPRC, 1168658 bp upstream of NR5A2	clus-181b-1
hsa-mir-181a-1	intergenic, > 10kb	101627 bp downstream of PTPRC, 1168487 bp upstream of NR5A2	clus-181b-1
hsa-mir-516-2	intergenic, > 10kb	124123 bp downstream of DPRX, 32380 bp upstream of NLRP12	clus-516-2
hsa-mir-519a-2	intergenic, > 10kb	125334 bp downstream of DPRX, 31172 bp upstream of NLRP12	clus-516-2
hsa-mir-506	intergenic, > 10kb	1400869 bp downstream of CXorf1, 681147 bp upstream of FMR1	clus-507
hsa-mir-507	intergenic, > 10kb	1401133 bp downstream of CXorf1, 680913 bp upstream of FMR1	clus-507
hsa-mir-29a	intergenic, > 10kb	142645 bp downstream of KLF14, 67351 bp upstream of FLJ43663	clus-29a
hsa-mir-29b-1	intergenic, > 10kb	143357 bp downstream of KLF14, 66622 bp upstream of FLJ43663	clus-29a
hsa-mir-514-1	intergenic, > 10kb	1449396 bp downstream of CXorf1, 632646 bp upstream of FMR1	clus-514
hsa-mir-514-2	intergenic, > 10kb	1452092 bp downstream of CXorf1, 629960 bp upstream of FMR1	clus-514
hsa-mir-514-3	intergenic, > 10kb	1454790 bp downstream of CXorf1, 627262 bp upstream of FMR1	clus-514
hsa-mir-127	intergenic, > 10kb	147858 bp downstream of DLK1, 122232 bp upstream of FLJ41170	clus-127
hsa-mir-432	intergenic, > 10kb	149362 bp downstream of DLK1, 120731 bp upstream of FLJ41170	clus-127
hsa-mir-136	intergenic, > 10kb	149581 bp downstream of DLK1, 120524 bp upstream of FLJ41170	clus-127
hsa-mir-379	intergenic, > 10kb	15033 bp downstream of FLJ41170, 539218 bp upstream of DIO3	clus-379
hsa-mir-411	intergenic, > 10kb	16292 bp downstream of FLJ41170, 537930 bp upstream of DIO3	clus-379
hsa-mir-299	intergenic, > 10kb	16761 bp downstream of FLJ41170, 537494 bp upstream of DIO3	clus-379

hsa-mir-500	intergenic, > 10kb	174470 bp downstream of PAGE4, 59092 bp upstream of CLCN5	clus-500
hsa-mir-362	intergenic, > 10kb	175003 bp downstream of PAGE4, 58578 bp upstream of CLCN5	clus-500
hsa-mir-501	intergenic, > 10kb	175761 bp downstream of PAGE4, 57801 bp upstream of CLCN5	clus-500
hsa-mir-96	intergenic, > 10kb	17611 bp downstream of NRF1, 58385 bp upstream of UBE2H	clus-183
hsa-mir-183	intergenic, > 10kb	17824 bp downstream of NRF1, 58140 bp upstream of UBE2H	clus-183
hsa-mir-380	intergenic, > 10kb	17984 bp downstream of FLJ41170, 536273 bp upstream of DIO3	clus-379
hsa-mir-363	intergenic, > 10kb	183741 bp downstream of GPC3, 203859 bp upstream of PHF6	clus-106a
hsa-mir-92-2	intergenic, > 10kb	183901 bp downstream of GPC3, 203699 bp upstream of PHF6	clus-106a
hsa-mir-19b-2	intergenic, > 10kb	184034 bp downstream of GPC3, 203545 bp upstream of PHF6	clus-106a
hsa-mir-20b	intergenic, > 10kb	184172 bp downstream of GPC3, 203434 bp upstream of PHF6	clus-106a
hsa-mir-18b	intergenic, > 10kb	184404 bp downstream of GPC3, 203200 bp upstream of PHF6	clus-106a
hsa-mir-106a	intergenic, > 10kb	184561 bp downstream of GPC3, 203033 bp upstream of PHF6	clus-106a
hsa-mir-323	intergenic, > 10kb	18699 bp downstream of FLJ41170, 535533 bp upstream of DIO3	clus-379
hsa-mir-368	intergenic, > 10kb	32657 bp downstream of FLJ41170, 521595 bp upstream of DIO3	clus-368
hsa-mir-376a-2	intergenic, > 10kb	33036 bp downstream of FLJ41170, 521202 bp upstream of DIO3	clus-368
hsa-mir-654	intergenic, > 10kb	33186 bp downstream of FLJ41170, 521051 bp upstream of DIO3	clus-368
hsa-mir-376b	intergenic, > 10kb	33403 bp downstream of FLJ41170, 520815 bp upstream of DIO3	clus-368
hsa-mir-376a-1	intergenic, > 10kb	33749 bp downstream of FLJ41170, 520501 bp upstream of DIO3	clus-368
hsa-mir-450-1	intergenic, > 10kb	39674 bp downstream of HPRT1, 25411 bp upstream of PLAC1	clus-450
hsa-mir-450-2	intergenic, > 10kb	39841 bp downstream of HPRT1, 25235 bp upstream of PLAC1	clus-450
hsa-mir-503	intergenic, > 10kb	45661 bp downstream of HPRT1, 19444 bp upstream of PLAC1	clus-424
hsa-mir-424	intergenic, > 10kb	45947 bp downstream of HPRT1, 19131 bp upstream of PLAC1	clus-424
hsa-mir-382	intergenic, > 10kb	47273 bp downstream of FLJ41170, 506969 bp upstream of DIO3	clus-382
hsa-mir-134	intergenic, > 10kb	47654 bp downstream of FLJ41170, 506591 bp upstream of DIO3	clus-382
hsa-mir-143	intergenic, > 10kb	49642 bp downstream of IL17B, 66258 bp upstream of CSNK1A1	clus-143
hsa-mir-200b	intergenic, > 10kb	51014 bp downstream of C1orf159, 12498 bp upstream of TTLL10	clus-200b
hsa-mir-145	intergenic, > 10kb	51370 bp downstream of IL17B, 64548 bp upstream of CSNK1A1	clus-143
hsa-mir-200a	intergenic, > 10kb	51773 bp downstream of C1orf159, 11744 bp upstream of TTLL10	clus-200b
hsa-mir-154	intergenic, > 10kb	52722 bp downstream of FLJ41170, 501512 bp upstream of DIO3	clus-154
hsa-mir-429	intergenic, > 10kb	52915 bp downstream of C1orf159, 10609 bp upstream of TTLL10	clus-200b
hsa-mir-496	intergenic, > 10kb	53540 bp downstream of FLJ41170, 500676 bp upstream of DIO3	clus-154
hsa-mir-221	intergenic, > 10kb	545438 bp downstream of CXorf36, 701066 bp upstream of ZNF673	clus-221
hsa-mir-222	intergenic, > 10kb	546274 bp downstream of CXorf36, 700230 bp upstream of ZNF673	clus-221
hsa-mir-377	intergenic, > 10kb	55017 bp downstream of FLJ41170, 499232 bp upstream of DIO3	clus-154
hsa-let-7a-3	intergenic, > 10kb	58604 bp downstream of C22orf26, 37796 bp upstream of PPARA	clus-let-7a-3
hsa-let-7b	intergenic, > 10kb	59541 bp downstream of C22orf26, 36850 bp upstream of PPARA	clus-let-7a-3
hsa-mir-99b	intergenic, > 10kb	62146 bp downstream of SIGLEC5, 20430 bp upstream of HAS1	clus-99b
hsa-let-7e	intergenic, > 10kb	62320 bp downstream of SIGLEC5, 20247 bp upstream of HAS1	clus-99b
hsa-mir-125a	intergenic, > 10kb	62788 bp downstream of SIGLEC5, 19772 bp upstream of HAS1	clus-99b
hsa-let-7a-1	intergenic, > 10kb	66102 bp downstream of PTPDC1, 83259 bp upstream of ZNF169	clus-let-7a-1
hsa-let-7f-1	intergenic, > 10kb	66492 bp downstream of PTPDC1, 82862 bp upstream of ZNF169	clus-let-7a-1
hsa-mir-526a-1	intergenic, > 10kb	69242 bp downstream of DPRX, 87266 bp upstream of NLRP12	clus-526a-1
hsa-mir-520c	intergenic, > 10kb	70443 bp downstream of DPRX, 86063 bp upstream of NLRP12	clus-526a-1
hsa-mir-518c	intergenic, > 10kb	71725 bp downstream of DPRX, 84767 bp upstream of NLRP12	clus-526a-1
hsa-mir-524	intergenic, > 10kb	73992 bp downstream of DPRX, 82514 bp upstream of NLRP12	clus-526a-1
hsa-mir-517a	intergenic, > 10kb	75258 bp downstream of DPRX, 81248 bp upstream of NLRP12	clus-526a-1
hsa-mir-519d	intergenic, > 10kb	76337 bp downstream of DPRX, 80168 bp upstream of NLRP12	clus-526a-1
