# Supplementary material for: Dissecting microregulation of a master regulatory network
Source: BMC Genomics. 2008 Feb 23;9:88. doi: 10.1186/1471-2164-9-88 (PMC2289817; doi:10.1186/1471-2164-9-88)
Supplement: Additional File 8 — Manually compiled list of miRNAs that are reported in the literature as either up or down regulated in various human cancers or cancer cell lines. This file has a list of miRNAs that are reported in the literature as either up or down regulated in various human cancers or cancer cell lines. These "cancer-associated" miRNAs were used to prioritize putative p53-miR targets. [file 1471-2164-9-88-S8.pdf]

Additional File 8: List of miRNAs that are reported in the literature as either up or down regulated in various human cancers or cancer cell lines. The target genes indicate only those that have been experimentally supported (from Tarbase http://www.diana.pcbi.upenn.edu/tarbase.html).

Cancer	miRNA	Up/Down	TargetGenes	Reference
Colorectal neoplasia	hsa-miR-133b	Down		Bandres et al., 2006
Colorectal neoplasia	hsa-miR-145	Down		Bandres et al., 2006
Chronic lymphocytic leukemia	hsa-miR-15	Down	BCL2	Calin et al., 2004; Cimmino et al., 2005
Chronic lymphocytic leukemia	hsa-miR-16	Down	BCL2	Calin et al., 2004; Cimmino et al., 2005
Glioblastoma	hsa-miR-181a	Down		Ciafre et al., 2005
Glioblastoma	hsa-miR-30c	Down		Ciafre et al., 2005
Brain cancer	hsa-miR-181	Down		Ciafre et al., 2005; Chan et al., 2005
B-cell lymphoma	hsa-miR-16-1	Down		Eis et al., 2005
Cervix cancer	hsa-miR-145	Down		Esquela-Kerscher and Slack, 2006
Cervix cancer	hsa-miR-143	Down	MAPK7	Esquela-Kerscher and Slack, 2006; Lui et al., 2007
CNS Tumor-derived cell line	hsa-let-7d	Down		Gaur et al, 2007
CNS Tumor-derived cell line	hsa-let-7g	Down		Gaur et al, 2007
CNS Tumor-derived cell line	hsa-let-7i	Down		Gaur et al, 2007
CNS Tumor-derived cell line	hsa-miR-103	Down		Gaur et al, 2007
CNS Tumor-derived cell line	hsa-miR-107	Down		Gaur et al, 2007
Hematologic Tumor-derived cell line	hsa-miR-10a	Down		Gaur et al, 2007
CNS Tumor-derived cell line	hsa-miR-124a	Down		Gaur et al, 2007
CNS Tumor-derived cell line	hsa-miR-128a	Down		Gaur et al, 2007
CNS Tumor-derived cell line	hsa-miR-128b	Down		Gaur et al, 2007
CNS Tumor-derived cell line	hsa-miR-129	Down		Gaur et al, 2007
ColonTumor-derived cell line	hsa-miR-130a	Down		Gaur et al, 2007
CNS Tumor-derived cell line	hsa-miR-134	Down		Gaur et al, 2007
CNS Tumor-derived cell line	hsa-miR-135a	Down		Gaur et al, 2007
CNS Tumor-derived cell line	hsa-miR-137	Down		Gaur et al, 2007
CNS Tumor-derived cell line	hsa-miR-138	Down		Gaur et al, 2007
ColonTumor-derived cell line	hsa-miR-148a	Down		Gaur et al, 2007
CNS Tumor-derived cell line	hsa-miR-148b	Down		Gaur et al, 2007
CNS Tumor-derived cell line	hsa-miR-149	Down		Gaur et al, 2007
CNS Tumor-derived cell line	hsa-miR-153	Down		Gaur et al, 2007
ColonTumor-derived cell line	hsa-miR-15a	Down		Gaur et al, 2007
CNS Tumor-derived cell line	hsa-miR-17-3p	Down		Gaur et al, 2007
CNS Tumor-derived cell line	hsa-miR-181a	Down		Gaur et al, 2007
CNS Tumor-derived cell line	hsa-miR-181a	Down		Gaur et al, 2007
CNS Tumor-derived cell line	hsa-miR-181b	Down		Gaur et al, 2007
CNS Tumor-derived cell line	hsa-miR-181c	Down		Gaur et al, 2007
CNS Tumor-derived cell line	hsa-miR-187	Down		Gaur et al, 2007
CNS Tumor-derived cell line	hsa-miR-191	Down		Gaur et al, 2007

CNS Tumor-derived cell line	hsa-miR-192	Down		Gaur et al, 2007
CNS Tumor-derived cell line	hsa-miR-194	Down		Gaur et al, 2007
Hematologic Tumor-derived cell line	hsa-miR-196b	Down		Gaur et al, 2007
CNS Tumor-derived cell line	hsa-miR-197	Down		Gaur et al, 2007
CNS Tumor-derived cell line	hsa-miR-203	Down		Gaur et al, 2007
CNS Tumor-derived cell line	hsa-miR-20a	Down		Gaur et al, 2007
CNS Tumor-derived cell line	hsa-miR-212	Down		Gaur et al, 2007
ColonTumor-derived cell line	hsa-miR-214	Down		Gaur et al, 2007
CNS Tumor-derived cell line	hsa-miR-219	Down		Gaur et al, 2007
CNS Tumor-derived cell line	hsa-miR-26b	Down		Gaur et al, 2007
Hematologic Tumor-derived cell line	hsa-miR-27b	Down		Gaur et al, 2007
Hematologic Tumor-derived cell line	hsa-miR-28	Down		Gaur et al, 2007
CNS Tumor-derived cell line	hsa-miR-30b	Down		Gaur et al, 2007
CNS Tumor-derived cell line	hsa-miR-30b	Down		Gaur et al, 2007
CNS Tumor-derived cell line	hsa-miR-30c	Down		Gaur et al, 2007
CNS Tumor-derived cell line	hsa-miR-30d	Down		Gaur et al, 2007
CNS Tumor-derived cell line	hsa-miR-32	Down		Gaur et al, 2007
CNS Tumor-derived cell line	hsa-miR-323	Down		Gaur et al, 2007
CNS Tumor-derived cell line	hsa-miR-324-3p	Down		Gaur et al, 2007
CNS Tumor-derived cell line	hsa-miR-324-5p	Down		Gaur et al, 2007
CNS Tumor-derived cell line	hsa-miR-328	Down		Gaur et al, 2007
CNS Tumor-derived cell line	hsa-miR-330	Down		Gaur et al, 2007
CNS Tumor-derived cell line	hsa-miR-331	Down		Gaur et al, 2007
CNS Tumor-derived cell line	hsa-miR-338	Down		Gaur et al, 2007
CNS Tumor-derived cell line	hsa-miR-340	Down		Gaur et al, 2007
CNS Tumor-derived cell line	hsa-miR-345	Down		Gaur et al, 2007
CNS Tumor-derived cell line	hsa-miR-346	Down		Gaur et al, 2007
CNS Tumor-derived cell line	hsa-miR-34a	Down		Gaur et al, 2007
CNS Tumor-derived cell line	hsa-miR-34a	Down		Gaur et al, 2007
CNS Tumor-derived cell line	hsa-miR-361	Down		Gaur et al, 2007
CNS Tumor-derived cell line	hsa-miR-370	Down		Gaur et al, 2007
ColonTumor-derived cell line	hsa-miR-378	Down		Gaur et al, 2007
CNS Tumor-derived cell line	hsa-miR-382	Down		Gaur et al, 2007
CNS Tumor-derived cell line	hsa-miR-383	Down		Gaur et al, 2007
ColonTumor-derived cell line	hsa-miR-422a	Down		Gaur et al, 2007
ColonTumor-derived cell line	hsa-miR-424	Down		Gaur et al, 2007
CNS Tumor-derived cell line	hsa-miR-425	Down		Gaur et al, 2007
CNS Tumor-derived cell line	hsa-miR-7	Down		Gaur et al, 2007

CNS Tumor-derived cell line	hsa-miR-7	Down		Gaur et al, 2007
CNS Tumor-derived cell line	hsa-miR-98	Down		Gaur et al, 2007
B-cell lymphoma	hsa-miR-143	Down		He et al., 2005
B-cell lymphoma	hsa-miR-17-3p	Down		He et al., 2005
Breast cancer	hsa-miR-10b	Down		Iorio et al., 2005
Breast cancer	hsa-miR-125b	Down		Iorio et al., 2005
Breast cancer	hsa-miR-145	Down		Iorio et al., 2005
Colon cancer	hsa-miR-130a	Down		Lu et al, 2005
Colon cancer	hsa-miR-181a	Down		Lu et al, 2005
Colorectal neoplasia	hsa-miR-143	Down		Michael et al., 2003
Colorectal neoplasia	hsa-miR-145	Down		Michael et al., 2003
Hepatocellular carcinoma	hsa-miR-125	Down		Murakami et al., 2006
Hepatocellular carcinoma	hsa-miR-18	Down		Murakami et al., 2006
Hepatocellular carcinoma	hsa-miR-195	Down		Murakami et al., 2006
Hepatocellular carcinoma	hsa-miR-199	Down		Murakami et al., 2006
Hepatocellular carcinoma	hsa-miR-200	Down		Murakami et al., 2006
Hepatocellular carcinoma	hsa-miR-224	Down		Murakami et al., 2006
B-cell lymphoma	hsa-miR-145	Down		O'Donnell et al., 2005
Renal cancer	hsa-miR-23b	Down		O'Rourke et al., 2006
Renal cancer	hsa-miR-24-1	Down		O'Rourke et al., 2006
Prostate cancer	hsa-let-7a	Down		Porkka et al., 2007
Prostate cancer	hsa-let-7b	Down		Porkka et al., 2007
Prostate cancer	hsa-let-7c	Down		Porkka et al., 2007
Prostate cancer	hsa-let-7d	Down		Porkka et al., 2007
Prostate cancer	hsa-let-7f	Down		Porkka et al., 2007
Prostate cancer	hsa-let-7g	Down		Porkka et al., 2007
Prostate cancer	hsa-miR-100	Down		Porkka et al., 2007
Prostate cancer	hsa-miR-103	Down		Porkka et al., 2007
Prostate cancer	hsa-miR-125a	Down		Porkka et al., 2007
Prostate cancer	hsa-miR-125b	Down		Porkka et al., 2007
Prostate cancer	hsa-miR-141	Down		Porkka et al., 2007
Prostate cancer	hsa-miR-143	Down		Porkka et al., 2007
Prostate cancer	hsa-miR-145	Down		Porkka et al., 2007
Prostate cancer	hsa-miR-148a	Down		Porkka et al., 2007
Prostate cancer	hsa-miR-16	Down		Porkka et al., 2007
Prostate cancer	hsa-miR-195	Down		Porkka et al., 2007
Prostate cancer	hsa-miR-199a	Down		Porkka et al., 2007
Prostate cancer	hsa-miR-199a*	Down		Porkka et al., 2007
Prostate cancer	hsa-miR-19b	Down		Porkka et al., 2007
Prostate cancer	hsa-miR-205	Down		Porkka et al., 2007

Prostate cancer	hsa-miR-22	Down		Porkka et al., 2007
Prostate cancer	hsa-miR-221	Down		Porkka et al., 2007
Prostate cancer	hsa-miR-222	Down		Porkka et al., 2007
Prostate cancer	hsa-miR-23a	Down		Porkka et al., 2007
Prostate cancer	hsa-miR-23b	Down		Porkka et al., 2007
Prostate cancer	hsa-miR-26a	Down		Porkka et al., 2007
Prostate cancer	hsa-miR-26b	Down		Porkka et al., 2007
Prostate cancer	hsa-miR-27a	Down		Porkka et al., 2007
Prostate cancer	hsa-miR-27b	Down		Porkka et al., 2007
Prostate cancer	hsa-miR-29a	Down		Porkka et al., 2007
Prostate cancer	hsa-miR-29b	Down		Porkka et al., 2007
Prostate cancer	hsa-miR-30a-5p	Down		Porkka et al., 2007
Prostate cancer	hsa-miR-30b	Down		Porkka et al., 2007
Prostate cancer	hsa-miR-30c	Down		Porkka et al., 2007
Prostate cancer	hsa-miR-497	Down		Porkka et al., 2007
Prostate cancer	hsa-miR-92	Down		Porkka et al., 2007
Prostate cancer	hsa-miR-99a	Down		Porkka et al., 2007
Lung cancer	hsa-let-7	Down	RAS, MYC	Takamizawa et al., 2004; Johnson et al., 2005; Hayashita et al., 2005; O'Donnell et al., 2005
Head and neck cancer cell lines	hsa-miR-127	Down		Tran et al., 2007
Head and neck cancer cell lines	hsa-miR-133a	Down		Tran et al., 2007
Head and neck cancer cell lines	hsa-miR-133b	Down		Tran et al., 2007
Head and neck cancer cell lines	hsa-miR-154	Down		Tran et al., 2007
Head and neck cancer cell lines	hsa-miR-200c	Down		Tran et al., 2007
Head and neck cancer cell lines	hsa-miR-212	Down		Tran et al., 2007
Head and neck cancer cell lines	hsa-miR-302b	Down		Tran et al., 2007
Head and neck cancer cell lines	hsa-miR-302c	Down		Tran et al., 2007
Head and neck cancer cell lines	hsa-miR-302d	Down		Tran et al., 2007
Head and neck cancer cell lines	hsa-miR-328	Down		Tran et al., 2007
Head and neck cancer cell lines	hsa-miR-340	Down		Tran et al., 2007
Head and neck cancer cell lines	hsa-miR-342	Down		Tran et al., 2007
Head and neck cancer cell lines	hsa-miR-345	Down		Tran et al., 2007
Head and neck cancer cell lines	hsa-miR-346	Down		Tran et al., 2007
Head and neck cancer cell lines	hsa-miR-371	Down		Tran et al., 2007
Head and neck cancer cell lines	hsa-miR-373	Down		Tran et al., 2007
Head and neck cancer cell lines	hsa-miR-375	Down		Tran et al., 2007
Head and neck cancer cell lines	hsa-miR-378	Down		Tran et al., 2007
Head and neck cancer cell lines	hsa-miR-382	Down		Tran et al., 2007
Head and neck cancer cell lines	hsa-miR-449	Down		Tran et al., 2007
Breast, colon, lung, pancreas, prostate, and stom	hsa-let-7a-3	Down		Volinia et al., 2006

Breast, colon, lung, pancreas, prostate, and stom	hsa-let-7g	Down		Volinia et al., 2006
Breast, colon, lung, pancreas, prostate, and stom	hsa-miR-125a	Down		Volinia et al., 2006
Breast, colon, lung, pancreas, prostate, and stom	hsa-miR-128b	Down		Volinia et al., 2006
Breast, colon, lung, pancreas, prostate, and stom	hsa-miR-132	Down		Volinia et al., 2006
Breast, colon, lung, pancreas, prostate, and stom	hsa-miR-141	Down		Volinia et al., 2006
Breast, colon, lung, pancreas, prostate, and stom	hsa-miR-16-1	Down		Volinia et al., 2006
Breast, colon, lung, pancreas, prostate, and stom	hsa-miR-16-2	Down		Volinia et al., 2006
Breast, colon, lung, pancreas, prostate, and stom	hsa-miR-17-5p	Down		Volinia et al., 2006
Breast, colon, lung, pancreas, prostate, and stom	hsa-miR-191	Down		Volinia et al., 2006
Breast, colon, lung, pancreas, prostate, and stom	hsa-miR-195	Down		Volinia et al., 2006
Breast, colon, lung, pancreas, prostate, and stom	hsa-miR-199a-1	Down		Volinia et al., 2006
Breast, colon, lung, pancreas, prostate, and stom	hsa-miR-199a-2	Down		Volinia et al., 2006
Breast, colon, lung, pancreas, prostate, and stom	hsa-miR-199b	Down		Volinia et al., 2006
Breast, colon, lung, pancreas, prostate, and stom	hsa-miR-200b	Down		Volinia et al., 2006
Breast, colon, lung, pancreas, prostate, and stom	hsa-miR-20a	Down		Volinia et al., 2006
Breast, colon, lung, pancreas, prostate, and stom	hsa-miR-24-2	Down		Volinia et al., 2006
Breast, colon, lung, pancreas, prostate, and stom	hsa-miR-27a	Down		Volinia et al., 2006
Breast, colon, lung, pancreas, prostate, and stom	hsa-miR-29a	Down		Volinia et al., 2006
Breast, colon, lung, pancreas, prostate, and stom	hsa-miR-29b-2	Down		Volinia et al., 2006
Breast, colon, lung, pancreas, prostate, and stom	hsa-miR-29c	Down		Volinia et al., 2006
Breast, colon, lung, pancreas, prostate, and stom	hsa-miR-34a	Down		Volinia et al., 2006
Breast, colon, lung, pancreas, prostate, and stom	hsa-miR-9-1	Down		Volinia et al., 2006
Breast, colon, lung, pancreas, prostate, and stom	hsa-miR-95	Down		Volinia et al., 2006
Colorectal neoplasia	hsa-miR-135b	Up		Bandres et al., 2006
Colorectal neoplasia	hsa-miR-183	Up		Bandres et al., 2006
Colorectal neoplasia	hsa-miR-31	Up		Bandres et al., 2006
Colorectal neoplasia	hsa-miR-96	Up		Bandres et al., 2006

Glioblastoma	hsa-miR-10b	Up		Chan et al., 2005
Breast cancer	hsa-miR-21	Up		Chan et al., 2005
Colon cancer	hsa-miR-21	Up		Chan et al., 2005
Glioblastoma	hsa-miR-21	Up		Chan et al., 2005
Lung cancer	hsa-miR-21	Up		Chan et al., 2005
Pancreatic Cancer	hsa-miR-21	Up		Chan et al., 2005
Prostate cancer	hsa-miR-21	Up		Chan et al., 2005
Stomach cancer	hsa-miR-21	Up		Chan et al., 2005
Brain cancer	hsa-miR-21	Up		Ciafre et al., 2005; Chan et al., 2005
Brain cancer	hsa-miR-221	Up		Ciafre et al., 2005; Chan et al., 2005
B-cell lymphoma	hsa-miR-19a	Up		Eis et al., 2005
Lymphomas	hsa-miR-155	Up	BIC	Eis et al., 2005; Metzler et al., 2004; He et al., 2005
Lymphomas	hsa-miR-17	Up	BIC	Eis et al., 2005; Metzler et al., 2004; He et al., 2005
Lymphomas	hsa-miR-18a	Up	BIC	Eis et al., 2005; Metzler et al., 2004; He et al., 2005
Lymphomas	hsa-miR-19a	Up	BIC	Eis et al., 2005; Metzler et al., 2004; He et al., 2005
Lymphomas	hsa-miR-19b-1	Up	BIC	Eis et al., 2005; Metzler et al., 2004; He et al., 2005
Lymphomas	hsa-miR-20a	Up	BIC	Eis et al., 2005; Metzler et al., 2004; He et al., 2005
Lymphomas	hsa-miR-92-1	Up	BIC	Eis et al., 2005; Metzler et al., 2004; He et al., 2005
B-cell lymphoma	hsa-miR-155	Up		Esquela-Kerscher and Slack, 2006
B-cell lymphoma	hsa-miR-221	Up		Esquela-Kerscher and Slack, 2006
B-cell lymphoma	hsa-miR-222	Up		Esquela-Kerscher and Slack, 2006
CNS Tumor-derived cell line	hsa-miR-10a	Up		Gaur et al, 2007
CNS Tumor-derived cell line	hsa-miR-196a	Up		Gaur et al, 2007
CNS Tumor-derived cell line	hsa-miR-196b	Up		Gaur et al, 2007
Thyroid cancer	hsa-miR-146	Up		He et al., 2005
Thyroid cancer	hsa-miR-221	Up		He et al., 2005
B-cell lymphoma	hsa-miR-92	Up		He et al., 2005
Papillary thyroid carcinoma	hsa-miR-146	Up	KIT	He et al., 2005b; Pallante et al., 2006
Papillary thyroid carcinoma	hsa-miR-181	Up	KIT	He et al., 2005b; Pallante et al., 2006
Papillary thyroid carcinoma	hsa-miR-221	Up	KIT	He et al., 2005b; Pallante et al., 2006
Papillary thyroid carcinoma	hsa-miR-222	Up	KIT	He et al., 2005b; Pallante et al., 2006
Breast cancer	hsa-miR-155	Up		Iorio et al., 2005
Breast cancer	hsa-miR-21	Up		Iorio et al., 2005
Cervix cancer	hsa-miR-21	Up		Lui et al., 2007
Cholangiocarcinoma cell line	hsa-miR-141	Up		Meng et al., 2006
Cholangiocarcinoma cell line	hsa-miR-200b	Up		Meng et al., 2006
Cholangiocarcinoma cell line	hsa-miR-21	Up		Meng et al., 2006
Lung cancer	hsa-miR-205	Up		Michael et al., 2003
B-cell lymphoma	hsa-miR-142	Up		O'Donnell et al., 2005
Prostate cancer	hsa-miR-184	Up		Porkka et al., 2007

Prostate cancer	hsa-miR-198	Up		Porkka et al., 2007
Prostate cancer	hsa-miR-202	Up		Porkka et al., 2007
Prostate cancer	hsa-miR-210	Up		Porkka et al., 2007
Prostate cancer	hsa-miR-296	Up		Porkka et al., 2007
Prostate cancer	hsa-miR-302c*	Up		Porkka et al., 2007
Prostate cancer	hsa-miR-320	Up		Porkka et al., 2007
Prostate cancer	hsa-miR-345	Up		Porkka et al., 2007
Prostate cancer	hsa-miR-370	Up		Porkka et al., 2007
Prostate cancer	hsa-miR-373*	Up		Porkka et al., 2007
Prostate cancer	hsa-miR-491	Up		Porkka et al., 2007
Prostate cancer	hsa-miR-498	Up		Porkka et al., 2007
Prostate cancer	hsa-miR-503	Up		Porkka et al., 2007
Prostate cancer	hsa-miR-513	Up		Porkka et al., 2007
Lung cancer	hsa-miR-17	Up	RAS, MYC	Takamizawa et al., 2004; Johnson et al., 2005; Hayashita et al., 2005; O'Donnell et al., 2005
Lung cancer	hsa-miR-18a	Up	RAS, MYC	Takamizawa et al., 2004; Johnson et al., 2005; Hayashita et al., 2005; O'Donnell et al., 2005
Lung cancer	hsa-miR-19a	Up	RAS, MYC	Takamizawa et al., 2004; Johnson et al., 2005; Hayashita et al., 2005; O'Donnell et al., 2005
Lung cancer	hsa-miR-19b-1	Up	RAS, MYC	Takamizawa et al., 2004; Johnson et al., 2005; Hayashita et al., 2005; O'Donnell et al., 2005
Lung cancer	hsa-miR-20a	Up	RAS, MYC	Takamizawa et al., 2004; Johnson et al., 2005; Hayashita et al., 2005; O'Donnell et al., 2005
Lung cancer	hsa-miR-92-1	Up	RAS, MYC	Takamizawa et al., 2004; Johnson et al., 2005; Hayashita et al., 2005; O'Donnell et al., 2005
Head and neck cancer cell lines	hsa-let-7a	Up		Tran et al., 2007
Head and neck cancer cell lines	hsa-let-7b	Up		Tran et al., 2007
Head and neck cancer cell lines	hsa-let-7c	Up		Tran et al., 2007
Head and neck cancer cell lines	hsa-let-7d	Up		Tran et al., 2007
Head and neck cancer cell lines	hsa-let-7f	Up		Tran et al., 2007
Head and neck cancer cell lines	hsa-miR-100	Up		Tran et al., 2007
Head and neck cancer cell lines	hsa-miR-103	Up		Tran et al., 2007
Head and neck cancer cell lines	hsa-miR-107	Up		Tran et al., 2007
Head and neck cancer cell lines	hsa-miR-125b	Up		Tran et al., 2007
Head and neck cancer cell lines	hsa-miR-15a	Up		Tran et al., 2007
Head and neck cancer cell lines	hsa-miR-15b	Up		Tran et al., 2007
Head and neck cancer cell lines	hsa-miR-16	Up		Tran et al., 2007
Head and neck cancer cell lines	hsa-miR-18	Up		Tran et al., 2007
Head and neck cancer cell lines	hsa-miR-19a	Up		Tran et al., 2007
Head and neck cancer cell lines	hsa-miR-200a	Up		Tran et al., 2007

Head and neck cancer cell lines	hsa-miR-200b	Up		Tran et al., 2007
Head and neck cancer cell lines	hsa-miR-205	Up		Tran et al., 2007
Head and neck cancer cell lines	hsa-miR-21	Up		Tran et al., 2007
Head and neck cancer cell lines	hsa-miR-22	Up		Tran et al., 2007
Head and neck cancer cell lines	hsa-miR-221	Up		Tran et al., 2007
Head and neck cancer cell lines	hsa-miR-23a	Up		Tran et al., 2007
Head and neck cancer cell lines	hsa-miR-23b	Up		Tran et al., 2007
Head and neck cancer cell lines	hsa-miR-24	Up		Tran et al., 2007
Head and neck cancer cell lines	hsa-miR-27a	Up		Tran et al., 2007
Head and neck cancer cell lines	hsa-miR-28	Up		Tran et al., 2007
Head and neck cancer cell lines	hsa-miR-29b	Up		Tran et al., 2007
Head and neck cancer cell lines	hsa-miR-30b	Up		Tran et al., 2007
Head and neck cancer cell lines	hsa-miR-31	Up		Tran et al., 2007
Head and neck cancer cell lines	hsa-miR-320	Up		Tran et al., 2007
Head and neck cancer cell lines	hsa-miR-361	Up		Tran et al., 2007
Head and neck cancer cell lines	hsa-miR-98	Up		Tran et al., 2007
Breast, colon, lung, pancreas, prostate, and stom	hsa-let-7a	Up		Volinia et al., 2006
Breast, colon, lung, pancreas, prostate, and stom	hsa-let-7b	Up		Volinia et al., 2006
Breast, colon, lung, pancreas, prostate, and stom	hsa-let-7e	Up		Volinia et al., 2006
Colon, pancreas, prostate	hsa-miR-106a	Up	RB1	Volinia et al., 2006
Colon, pancreas, stomach	hsa-miR-107	Up		Volinia et al., 2006
Breast, colon, lung, pancreas, prostate, and stom	hsa-miR-128a	Up		Volinia et al., 2006
Colon, lung, pancreas	hsa-miR-128b	Up		Volinia et al., 2006
Breast, colon, lung, pancreas, prostate, and stom	hsa-miR-138-1	Up		Volinia et al., 2006
Breast, colon, lung, pancreas, prostate, and stom	hsa-miR-138-2	Up		Volinia et al., 2006
Breast, pancreas, prostate	hsa-miR-146	Up		Volinia et al., 2006
Breast, colon, lung	hsa-miR-155	Up		Volinia et al., 2006
Breast, colon, lung, pancreas, prostate, and stom	hsa-miR-155	Up		Volinia et al., 2006
Breast, colon, lung, pancreas, prostate	hsa-miR-17-5p	Up		Volinia et al., 2006
Breast, pancreas, prostate	hsa-miR-181b-1	Up		Volinia et al., 2006
Colon, lung, pancreas, prostate, stomach	hsa-miR-191	Up		Volinia et al., 2006
Lung, pancreas, prostate	hsa-miR-199a-1	Up		Volinia et al., 2006
Colon, pancreas, prostate	hsa-miR-20a	Up	TGFBR2	Volinia et al., 2006
Breast, colon, lung, pancreas, prostate, and stom	hsa-miR-21	Up		Volinia et al., 2006
Breast, colon, lung, pancreas, prostate, stomach	hsa-miR-21	Up		Volinia et al., 2006
Breast, colon, lung, pancreas, prostate, and stom	hsa-miR-212	Up		Volinia et al., 2006
Pancreas, prostate, stomach	hsa-miR-214	Up		Volinia et al., 2006
Breast, colon, lung, pancreas, prostate, and stom	hsa-miR-218-2	Up		Volinia et al., 2006
Colon, pancreas, stomach	hsa-miR-221	Up		Volinia et al., 2006
Colon, pancreas, prostate, stomach	hsa-miR-223	Up		Volinia et al., 2006

Breast, colon, lung, pancreas, prostate, and stom	hsa-miR-23b	Up		Volinia et al., 2006
Colon, pancreas, stomach	hsa-miR-24-1	Up		Volinia et al., 2006
Colon, pancreas, stomach	hsa-miR-24-2	Up		Volinia et al., 2006
Pancreas, prostate, stomach	hsa-miR-25	Up		Volinia et al., 2006
Breast, colon, pancreas, prostate	hsa-miR-29b-2	Up		Volinia et al., 2006
Colon, pancreas, prostate	hsa-miR-30c	Up		Volinia et al., 2006
Breast, colon, lung, pancreas, prostate, and stom	hsa-miR-30d	Up		Volinia et al., 2006
Colon, pancreas, prostate	hsa-miR-32	Up		Volinia et al., 2006
Breast, colon, lung, pancreas, prostate, and stom	hsa-miR-9-3	Up		Volinia et al., 2006
Breast, colon, lung, pancreas, prostate, and stom	hsa-miR-92-1	Up		Volinia et al., 2006
Pancreas, prostate, stomach	hsa-miR-92-2	Up		Volinia et al., 2006
Breast, colon, lung, pancreas, prostate, and stom	hsa-miR-96	Up		Volinia et al., 2006
Testicular germ cell tumors	hsa-miR-372	Up	LATS2	Voorhoeve et al., 2006
Testicular germ cell tumors	hsa-miR-373	Up	LATS2	Voorhoeve et al., 2006
Ovarian cancer	hsa-miR-210	Up		Yanaihara et al., 2006

References

1. Bandres E, Cubedo E, Agirre X, Malumbres R, Zarate R, Ramirez N, Abajo A, Navarro A, Moreno I, Monzo M *et al* : **Identification by Real-time PCR of 13 mature microRNAs differentially expressed in colorectal cancer and non-tumoral tissues**. *Mol Cancer* 2006, **5**:29.

2. Calin GA, Liu CG, Sevignani C, Ferracin M, Felli N, Dumitru CD, Shimizu M, Cimmino A, Zupo S, Dono M *et al* : **MicroRNA profiling reveals distinct signatures in B cell chronic lymphocytic leukemias**. *Proc Natl Acad Sci U S A* 2004, **101**(32):11755-11760.

3. Chan JA, Krichevsky AM, Kosik KS: **MicroRNA-21 is an antiapoptotic factor in human glioblastoma cells**. *Cancer Res* 2005, **65**(14):6029-6033.

4. Ciafre SA, Galardi S, Mangiola A, Ferracin M, Liu CG, Sabatino G, Negrini M, Maira G, Croce CM, Farace MG: **Extensive modulation of a set of microRNAs in primary glioblastoma**. *Biochem Biophys Res Commun* 2005, **334**(4):1351-1358.

5. Cimmino A, Calin GA, Fabbri M, Iorio MV, Ferracin M, Shimizu M, Wojcik SE, Aqeilan RI, Zupo S, Dono M *et al* : **miR-15 and miR-16 induce apoptosis by targeting BCL2**. *Proc Natl Acad Sci U S A* 2005, **102**(39):13944-13949.

6. Eis PS, Tam W, Sun L, Chadburn A, Li Z, Gomez MF, Lund E, Dahlberg JE: **Accumulation of miR-155 and BIC RNA in human B cell lymphomas**. *Proc Natl Acad Sci U S A* 2005, **102**(10):3627-3632.

7. Esquela-Kerscher A, Slack FJ: **Oncomirs - microRNAs with a role in cancer**. *Nat Rev Cancer* 2006, **6**(4):259-269.

8. Gaur A, Jewell DA, Liang Y, Ridzon D, Moore JH, Chen C, Ambros VR, Israel MA: **Characterization of microRNA expression levels and their biological correlates in human cancer cell lines**. *Cancer Res* 2007, **67**(6):2456-2468.

9. Hayashita Y, Osada H, Tatematsu Y, Yamada H, Yanagisawa K, Tomida S, Yatabe Y, Kawahara K, Sekido Y, Takahashi T: **A polycistronic microRNA cluster, miR-17-92, is overexpressed in human lung cancers and enhances cell proliferation**. *Cancer Res* 2005, **65**(21):9628-9632.

10. He H, Jazdzewski K, Li W, Liyanarachchi S, Nagy R, Volinia S, Calin GA, Liu CG, Franssila K, Suster S *et al* : **The role of microRNA genes in papillary thyroid carcinoma**. *Proc Natl Acad Sci U S A* 2005, **102**(52):19075-19080.

11. He L, Thomson JM, Hemann MT, Hernando-Monge E, Mu D, Goodson S, Powers S, Cordon-Cardo C, Lowe SW, Hannon GJ *et al* : **A microRNA polycistron as a potential human oncogene**. *Nature* 2005, **435**(7043):828-833.

12. Iorio MV, Ferracin M, Liu CG, Veronese A, Spizzo R, Sabbioni S, Magri E, Pedriali M, Fabbri M, Campiglio M *et al* : **MicroRNA gene expression deregulation in human breast cancer**. *Cancer Res* 2005, **65**(16):7065-7070.

13. Johnson SM, Grosshans H, Shingara J, Byrom M, Jarvis R, Cheng A, Labourier E, Reinert KL, Brown D, Slack FJ: **RAS is regulated by the let-7 microRNA family**. *Cell* 2005, **120**(5):635-647.

14. Lu J, Getz G, Miska EA, Alvarez-Saavedra E, Lamb J, Peck D, Sweet-Cordero A, Ebert BL, Mak RH, Ferrando AA *et al* : **MicroRNA expression profiles classify human cancers**. *Nature* 2005, **435**(7043):834-838.

15. Lui WO, Pourmand N, Patterson BK, Fire A: **Patterns of known and novel small RNAs in human cervical cancer**. *Cancer Res* 2007, **67**(13):6031-6043.

16. Meng F, Henson R, Lang M, Wehbe H, Maheshwari S, Mendell JT, Jiang J, Schmittgen TD, Patel T: **Involvement of human micro-RNA in growth and response to chemotherapy in human cholangiocarcinoma cell lines**. *Gastroenterology* 2006, **130**(7):2113-2129.

17. Metzler M, Wilda M, Busch K, Viehmann S, Borkhardt A: **High expression of precursor microRNA-155/BIC RNA in children with Burkitt lymphoma**. *Genes Chromosomes Cancer* 2004, **39**(2):167-169.

18. Michael MZ, SM OC, van Holst Pellekaan NG, Young GP, James RJ: **Reduced accumulation of specific microRNAs in colorectal neoplasia**. *Mol Cancer Res* 2003, **1**(12):882-891.

19. Murakami Y, Yasuda T, Saigo K, Urashima T, Toyoda H, Okanoue T, Shimotohno K: **Comprehensive analysis of microRNA expression patterns in hepatocellular carcinoma and non-tumorous tissues**. *Oncogene* 2006, **25**(17):2537-2545.

20. O'Donnell KA, Wentzel EA, Zeller KI, Dang CV, Mendell JT: **c-Myc-regulated microRNAs modulate E2F1 expression**. *Nature* 2005, **435**(7043):839-843.

21. O'Rourke JR, Swanson MS, Harfe BD: **MicroRNAs in mammalian development and tumorigenesis**. *Birth Defects Res C Embryo Today* 2006, **78**(2):172-179.

22. Pallante P, Visone R, Ferracin M, Ferraro A, Berlingieri MT, Troncone G, Chiappetta G, Liu CG, Santoro M, Negrini M *et al* : **MicroRNA deregulation in human thyroid papillary carcinomas**. *Endocr Relat Cancer* 2006, **13**(2):497-508.

23. Porkka KP, Pfeiffer MJ, Waltering KK, Vessella RL, Tammela TL, Visakorpi T: **MicroRNA expression profiling in prostate cancer**. *Cancer Res* 2007, **67**(13):6130-6135.

24. Takamizawa J, Konishi H, Yanagisawa K, Tomida S, Osada H, Endoh H, Harano T, Yatabe Y, Nagino M, Nimura Y *et al* : **Reduced expression of the let-7 microRNAs in human lung cancers in association with shortened postoperative survival**. *Cancer Res* 2004, **64**(11):3753-3756.

25. Tran N, McLean T, Zhang X, Zhao CJ, Thomson JM, O'Brien C, Rose B: **MicroRNA expression profiles in head and neck cancer cell lines**. *Biochem Biophys Res Commun* 2007, **358**(1):12-17.

26. Volinia S, Calin GA, Liu CG, Ambs S, Cimmino A, Petrocca F, Visone R, Iorio M, Roldo C, Ferracin M *et al* : **A microRNA expression signature of human solid tumors defines cancer gene targets**. *Proc Natl Acad Sci U S A* 2006, **103**(7):2257-2261.

27. Voorhoeve PM, le Sage C, Schrier M, Gillis AJ, Stoop H, Nagel R, Liu YP, van Duijse J, Drost J, Griekspoor A *et al* : **A genetic screen implicates miRNA-372 and miRNA-373 as oncogenes in testicular germ cell tumors**. *Cell* 2006, **124**(6):1169-1181.

28. Yanaihara N, Caplen N, Bowman E, Seike M, Kumamoto K, Yi M, Stephens RM, Okamoto A, Yokota J, Tanaka T *et al* : **Unique microRNA molecular profiles in lung cancer diagnosis and prognosis**. *Cancer Cell* 2006, **9**(3):189-198.
